# Comparative proteomic network signatures in seminal plasma of infertile men as a function of reactive oxygen species

**DOI:** 10.1186/s12014-015-9094-5

**Published:** 2015-08-28

**Authors:** Ashok Agarwal, Ahmet Ayaz, Luna Samanta, Rakesh Sharma, Mourad Assidi, Adel M. Abuzenadah, Edmund Sabanegh

**Affiliations:** American Center for Reproductive Medicine, Department of Urology, Cleveland Clinic, Cleveland, OH 44195 USA; Department of Zoology, School of Life Sciences, Ravenshaw University, Cuttack, Odisha 751003 India; Center of Excellence in Genomic Medicine Research, King AbdulAziz University, Jeddah, Saudi Arabia; KACST Technology Innovation Center in Personalized Medicine at King AbdulAziz University, Jeddah, Saudi Arabia; Department of Urology, Cleveland Clinic, Cleveland, OH 44195 USA

**Keywords:** Seminal plasma, Proteome, Reactive oxygen species, Sperm function, Infertility

## Abstract

**Background:**

Reactive oxygen species (ROS) plays a major role in the pathology of male infertility. It is an independent biomarker of sperm function. Seminal plasma is a natural reservoir of antioxidants responsible for the nourishment, protection, capacitation, and motility of sperm within the female reproductive tract resulting in successful fertilization and implantation of the embryo. A comparative proteomic analysis of seminal plasma proteins from fertile men and infertile men with varying levels of ROS was carried out to identify signature proteins involved in ROS-mediated reproductive dysfunction.

**Methods:**

A total of 42 infertile men presenting with infertility and 17 proven fertile donors were enrolled in the study. ROS levels were measured in the seminal ejaculates by chemiluminescence assay. Infertile men were subdivided into Low ROS (0–<93 RLU/s/10^6^ sperm; n = 11), Medium ROS (>93–500 RLU/s/10^6^ sperm; n = 17) and High ROS (>500 RLU/s/10^6^ sperm; n = 14) groups and compared with fertile men (4–50 RLU/s/10^6^ sperm). 4 subjects from fertile group and 4 each from the Low, Medium and High ROS were pooled. 1D gel electrophoresis followed by in-gel digestion and LC/MS–MS in a LTQ-Orbitrap Elite hybrid mass spectrometer system was used for proteome analysis. Identification of differentially expressed proteins (DEPs), their cellular localization and involvement in different pathways were examined utilizing bioinformatics tools.

**Results:**

The results indicate that proteins involved in biomolecule metabolism, protein folding and protein degradation are differentially modulated in all three infertile patient groups in comparison to fertile controls. Membrane metallo-endopeptidase (MME) was uniformly overexpressed (>2 fold) in all infertile groups. Pathway involving 35 focus proteins in post-translational modification of proteins, protein folding (heat shock proteins, molecular chaperones) and developmental disorder was overexpressed in the High ROS group compared with fertile control group. MME was one of the key proteins in the pathway. FAM3D was uniquely expressed in fertile group.

**Conclusion:**

We have for the first time demonstrated the presence of 35 DEPs of a single pathway that may lead to impairment of sperm function in men with Low, Medium or High ROS levels by altering protein turn over. MME and FAM3D along with ROS levels in the seminal plasma may serve as good markers for diagnosis of male infertility.

**Electronic supplementary material:**

The online version of this article (doi:10.1186/s12014-015-9094-5) contains supplementary material, which is available to authorized users.

## Background

Seminal plasma contains secretions that are derived from the testis, epididymis and male accessory glands, including the prostate, seminal vesicles and Cowper’s gland. A considerable volume of research has focused on the mechanism(s) involved in production, morphology and function of spermatozoa, however, the critical role that seminal fluid plays in imparting motility and fertilization capacity to sperm is often neglected. Seminal fluid proteins are involved in the nourishment, protection, capacitation, and motility of sperm within the female reproductive tract and therefore, expected to contribute to the success of fertilization [1]. For example, the fertilization-promoting peptide (FPP) in semen stimulates penetration abilities of spermatozoa promoting fertilization [2]. Seminal fluid interacts with female reproductive tract, enhances implantation rates and early embryo development [3], increases the levels of uterine granulocyte macrophage colony stimulating factor in mice (GM-CSF) [4] and upregulates the transcription factor forkhead box P3 (FOXP3) in uterine T-cells [5], thereby improving tolerance towards paternal antigens [6]. In addition, an increasing number of seminal plasma proteins, such as insulin-like growth factor-I, alpha2-macroglobulin and the encephalin degrading enzymes, have been shown to be associated with sperm motility [7–9].

A common end to numerous pathways that lead to defective sperm function is attributed to reactive oxygen species (ROS), a group of molecules with incompletely reduced oxygen atom [10, 11] that are capable of reacting with almost all biomolecules leading to their altered function such as inhibition/activation of enzymes. Nature has bestowed aerobic organisms with an array of antioxidant defence mechanism(s) to fight off the noxious effects of ROS. However, when their rate of generation exceeds the cell’s antioxidant defence, it leads to oxidative stress. ROS are highly reactive, non-specific and autocatalytic which qualify them to be good signalling molecules. Physiological levels of ROS is necessarily maintained in all aerobic cells [12] as well as in the semen for optimal sperm function such as capacitation, motility and acrosome reaction [13, 14]. While both leukocyte and spermatozoa serve as principal sources of ROS generation in semen, the spermatozoa are more susceptible to ROS—induced damage at stake against ROS due to its rich content of polyunsaturated fatty acids and poor antioxidant capacity. The first report on harmful effects of ROS on spermatozoa was published over 60 years ago [15] and a large body of literature has provided growing support for the concept that abnormal semen parameters and sperm damage are consequences of excessive levels of ROS resulting in impaired sperm function and subfertility [10, 16–19]. Consensus is growing about the clinical utility of seminal oxidative stress testing in infertility clinics [20, 21].

A common pathology seen in two-thirds of infertile cases is attributed to oxidative stress in semen, although by different mechanisms [22]. A previous report from our laboratory suggests that high levels of ROS could be an independent marker of male factor infertility [20]. Because proteins have many different and unique biological functions, oxidative modifications to proteins can lead to diverse functional consequences. In fact, previous reports from various laboratories, including ours, suggest global change in proteomic profile of human spermatozoa and seminal plasma under oxidative stress conditions [23–26]. Recently, we have reported the differential regulation of protein expression in infertile patients with variations in ROS levels as evidenced by global proteomic profiling [27]. Extracellular antioxidants are secreted by the male reproductive tract for minimizing oxidative stress suffered by spermatozoa throughout the post-testicular phase of sperm existence. Such protection is apparent in the epididymis [28]. However, at the time of ejaculation, spermatozoa move out of a hypoxic epididymal environment into the well-vascularized lower female reproductive tract with higher oxygen tension [29]. Although spermatozoa spend a very short period of time in seminal plasma, in order to counteract such stress, seminal plasma is well-endowed with high levels of antioxidants, almost 10 times higher than that of blood [30].This is in line with the role of seminal plasma in protecting oxidative DNA damage of spermatozoa in the female reproductive tract [31]. Here we test the hypothesis that the seminal plasma proteome influences fertilization and implantation in infertile men with various levels of ROS. The present study is the first report on comparative proteomic analysis of seminal plasma as a function of ROS levels in infertile patients with respect to fertile donors.

## Methods

### Clinical sample

This prospective study is a continuation of our recently published report on spermatozoal proteins in infertile men with different levels of ROS [27]. All specimens were collected by masturbation at the Andrology Laboratory of Cleveland Clinic after 2–3 days of sexual abstinence. The Institutional Review Board of Cleveland Clinic approved the entire protocol used in the study. All subjects consented in writing to be enrolled in this prospective study. Semen samples were collected from infertile male patients with different levels of oxidative stress (n = 42) and healthy donors with proven fertility (n = 17).

### Inclusion/exclusion criteria

Men in the age group of 20–40 years who attended the clinic for infertility treatment from March 2012 to March 2014 were enrolled in the study. All female partners of these infertile men were otherwise normal as evidenced by their gynecologic evaluation results on fertility assessment. Patients who suffered from a recurring fever in the 90**-**day period prior to semen analysis or patients with leukocytospermia, azoospermia and oligozoospermia were excluded from the study. Donors with a proven fertility within past 2 years and in the same age group as patients were included as controls.

### Semen analysis

Manual semen analysis was performed using a MicroCell counting chamber (Vitrolife, San Diego, CA, USA) to determine sperm concentration and motility after complete liquefaction of the samples for 20 min at 37 °C. Sperm morphology according to World Health Organization 2010 [32] was assessed in the smears of the raw semen stained with a Diff-Quik kit (Baxter Healthcare Corporation, Inc., McGaw Park, IL). Leukocytospermia was recorded when the round cell concentration was >1 × 10^6^/mL in the sample and was further confirmed by the peroxidase or the Endtz test [25]. Specimens that were positive for the Endtz test (>1×10^6^ white blood cells/mL) indicative of an underlying infection were not included in the study.

### Reactive oxygen species (ROS) measurement

ROS formation was measured by chemiluminescence assay in the semen using 10 μL 5 mM luminol (5-amino-2,3-dihydro-1, 4-phthalazinedione) as the probe for 15 min using a Berthold luminometer (AutolumatPlus 953, Oakridge, TN, USA). Results were expressed as relative light units per second per million spermatozoa (RLU/sec/10^6^ sperm) [33]. Samples were divided into three groups based on the ROS levels [27]:

1. Low ROS group: ROS levels 0–<93 RLU/sec/10^6^ sperm
2. Medium ROS: ROS levels >93–500 RLU/sec/10^6^ sperm
3. High ROS group: ROS concentration >500 RLU/sec/10^6^ sperm

### Sample preparation and Protein extraction

We analyzed 17, 14 and 11 patients respectively in the three ROS groups. However, the sperm concentration in these samples varied significantly. In proteomic studies protein concentration used has to be normalized i.e. protein contribution from the spermatozoa in each group must be similar. In other words, the sperm concentration should also be normalized, i.e. equal amount of protein contributed by similar number of spermatozoa in each patient and the fertile group. This requirement eliminated majority of the samples from being used in the proteomic analysis. In order to perform LC–MS analysis; we pooled the samples in each group. Pooling of semen samples is acceptable in proteomic analysis. Therefore, to obtain the desired concentration of protein for proteomic analysis, the minimum number of samples that could give us the maximum protein concentration and sperm concentration in each group was four, and hence this was the minimum number of samples that were pooled in each group. This was also explained in our earlier publication [27] where contribution from each subject to the pool is normalized in terms of concentration of proteins. Since it is a continuation of the previous study to establish the factors present in spermatozoa and seminal plasma responsible for infertility, seminal plasma from the same pool was used for proteomic analysis. Spermatozoa and round cells were separated from the seminal plasma by centrifugation at ~10,000×*g* for 10 min. The seminal plasma was checked for the presence of spermatozoa, if any, and centrifuged again to get clear seminal plasma devoid of spermatozoa. It was mixed with the protease inhibitor cocktail (Roche, Indianapolis, IN, USA) in phosphate buffer saline in order to prevent proteolysis during sample handling and again centrifuged at ~10,000×*g* for 30 min to get rid of any cellular debris. Protein concentration in the seminal plasma was determined using bicinchoninic acid (BCA) kit (Thermo, Rockford, IL, USA).

### Global proteomics analysis

Equal amounts of proteins from each group were resolved on a 1D SDS-PAGE. For the protein digestion, 12 bands were cut to minimize excess polyacrylamide and divided into a number of smaller pieces. The gel pieces were washed with water and dehydrated in acetonitrile. The bands were then reduced with Dithiothreitol (DTT) and alkylated with iodoacetamide prior to the in-gel digestion. All bands were digested in-gel using trypsin, by adding 5 μL of 10 ng/μL trypsin in 50 mM ammonium bicarbonate and incubating overnight at room temperature to achieve complete digestion. The peptides formed were extracted from the polyacrylamide in two aliquots of 30 μL 50 % acetonitrile with 5 % formic acid. These extracts were combined and evaporated to <10 μL in Speedvac and then resuspended in 1 % acetic acid to make up a final volume of ~30 μL for LC–MS analysis. The LC–MS system was a Finnigan LTQ-Orbitrap Elite hybrid mass spectrometer system. The HPLC column was a Dionex 15 cm × 75 μm internal diameter Acclaim Pepmap C18, 2 μm, 100 Å reverse phase capillary chromatography column. The extracts (5 μL) were injected into the column and the peptides eluted by an acetonitrile/0.1 % formic acid gradient at a flow rate of 0.25 μL/min were introduced into the source of the mass spectrometer on-line. The microelectrospray ion source is operated at 2.5 kV. The digest was analyzed using the data dependent multitask capability of the instrument acquiring full scan mass spectra to determine peptide molecular weights and product ion spectra to determine amino acid sequence in successive instrument scans.

### Data analysis

For semen parameters, comparison was made between fertile men and patients as well as fertile men and patients in each ROS group by Wilcoxon rank sum test. Tandem mass spectra were extracted by Proteome Discoverer version 1.4.1.288. Charge state deconvolution and de-isotoping was not performed. All MS/MS samples were analyzed using Mascot (Matrix Science, London, UK; version 2.3.02), SEQUEST (Thermo Fisher Scientific, San Jose, CA, USA; version 1.4.0.288) and X! Tandem (TheGPM, thegpm.org; version CYCLONE (2010.12.01.1). Mascot, Sequest and X! Tandem were set up to search the human reference with database (33,292 entries) assuming trypsin as the digestion enzyme. These searches were performed with a fragment ion mass tolerance of 0.8 Da, and a parent ion tolerance of 10 parts per million (PPM). Carbamidomethylation of cysteine was specified as a fixed modification, and oxidation of methionine was specified as a variable modification.

### Criteria for protein identification

To validate MS/MS-based peptide and protein identifications Scaffold (version 4.0.6.1, Proteome Software Inc., Portland, OR, USA) was used. Peptide identifications were accepted if they could be established at >95.0 % probability by the Peptide Prophet algorithm [34] with Scaffold delta-mass correction. Protein identifications were accepted if they could be established at >99.0 % probability to achieve a false discovery rate (FDR) of <1.0 % and contained at least 2 identified peptides. Protein probabilities were assigned by the Protein Prophet algorithm [35]. Proteins that contained similar peptides and could not be differentiated based on MS/MS analysis alone were grouped to satisfy the principles of parsimony. Proteins were annotated with gene ontology (GO) terms from National Center for Biotechnology Information (NCBI) (downloaded Oct 21, 2013) [36].

### Quantitative proteomics

For proteomic analysis, the relative quantity of the protein was determined by comparing the number of spectra (termed as spectral counts SpCs), used to identify each protein. The total number of mass spectra (SpC) that matched peptides to a particular protein was used to measure the abundance of proteins in the complex mixture. Normalization of spectral counts using the NSAF (normalized spectral abundance factor) approach was applied prior to relative protein quantification. DEPs were obtained by applying different constraints for significance tests and/or fold-change cutoffs based on the average SpC of the protein from multiple runs. Appropriate filters were used to identify DEPs that were dependent on the overall abundance of the proteins. It has been reported [37] that accurate quantification and determination of real biological change is dependent on the number of SpCs and hence, different constraints have to be applied to SpC levels in order to circumvent the biases and maintain a constant false positive ratio (FPR) for all proteins. The abundance of the proteins was classified as High (H), Medium (M), Low (L), or Very Low (VL) based on their average spectral counts amongst the 3 replicate runs. The error observed for the SpC measurements is greater for lower abundant proteins compared to higher abundant proteins. Due to this, different filtering criteria were used to determine if proteins are differentially present based on the overall abundance. The spectral count distribution for these samples is given below and a majority of the proteins have SpCs less than 20. Therefore, to normalize the values, the number of spectral counts for each protein was divided by the mass [38] or protein length, to get the spectral abundance factor (SAF) [39]. Individual SAF values were normalized to one by dividing by the sum of all SAFs for proteins in the complex, resulting in the NSAF value to accurately account for run to run variation [40]. Different constraints for significance tests (*p* value) and/or fold change cutoffs (or NSAF ratio) were applied for these four abundance categories, as shown below:

1. Very Low abundance: spectral count range 1.7–7; p ≤ 0.001 and NSAF ratio ≥2.5 for overexpressed, ≤0.4 for underexpressed proteins.
2. Low abundance: spectral count range 8–19; p ≤ 0.01 and NSAF ratio ≥2.5 for overexpressed, ≤0.4 for underexpressed proteins.
3. Medium abundance: spectral count range between 20 and 79; p ≤ 0.05 and NSAF ratio ≥2.0 for overexpressed, ≤0.5 for underexpressed proteins.
4. High abundance: spectral counts >80; p ≤ 0.05 and NSAF ratio ≥1.5 for overexpressed, ≤0.67 for underexpressed proteins.

### Bioinformatics analysis

Functional annotation and enrichment analysis were performed using publicly available bioinformatics annotation tools and databases such as GO Term Finder [41], GO Term Mapper, UniProt, Software Tools for Researching Annotations of Proteins (STRAP) [42], Database for Annotation, Visualization and Integrated Discovery (DAVID) (http://david.niaid.nih.gov), and proprietary software package such as IPA (Ingenuity Pathway Analysis) from Ingenuity^®^ Systems, used to obtain consensus-based, comprehensive functional context for the large list of proteins derived from proteomic study.

## Results

### Semen analysis

Sperm concentration, morphology and ROS levels were significantly different among the fertile men and infertile men. ROS levels in fertile men (n = 17) were within physiological limits (i.e., 4–50 RLU) whereas the infertile group (n = 42) had significantly elevated ROS levels with respect to control and were classified on the basis of gradient in ROS levels into three groups [27]. Of the 42 infertile men, 11 men had Low ROS levels between 0–<93 RLU/sec/10^6^ sperm (range 0–12.7 RLU/sec/10^6^ sperm), 17 had Medium ROS group levels >93–500 RLU/sec/10^6^ sperm (range 131.5–320.1 RLU/sec/10^6^ sperm) and 14 had High ROS levels >500 RLU/sec/10^6^ sperm (range 924.2–9395 RLU/sec/10^6^ sperm). Among the different ROS groups, a significant reduction in sperm concentration (from 53.60 ± 46.98 to 20.02 ± 33.45 × 10^6^/ml), motility (%) (from 47.7 ± 13.7 to 34.0 ± 17.1), and morphology (%) (from 7.7 ± 2.6 to 2.5 ± 1.8) was noticed in the High ROS group compared to the fertile group. Semen parameters in the other two ROS groups were comparable with the fertile group [27].

### Analysis of seminal plasma proteins

A total of 841 proteins were identified in all the 4 groups studied (Additional file 1: Table S1, Additional file 2: Table S2, Additional file 3: Table S3, Additional file 4: Table S4). Control fertile group had 572 proteins compared to 544, 612 and 670 proteins identified in infertile patients with Low, Medium and High ROS levels, respectively. Out of the total proteins identified in each group, 472 (83 %), 464 (85 %), 513 (84 %) and 568 (85 %) proteins were identified in all the three replicates analyzed for each of control, Low, Medium and High ROS groups, respectively. Similarly, 49 (9 %), 43 (8 %), 63 (10 %) and 73 (11 %) proteins were identified in two of the three replicates of control, Low, Medium and High ROS groups, respectively. On the other hand, 51 (9 %), 37 (7 %), 36 (6 %) and 29 (4 %) proteins were identified in only a single replicate of analyzed groups namely, control, Low, Medium and High ROS groups. Some of the most abundant proteins present in all the four groups were albumin, lactotransferrin isoform 1 and fibronectin isoform 3.

### Global expression profile of identified proteins

**Fig. 1:**
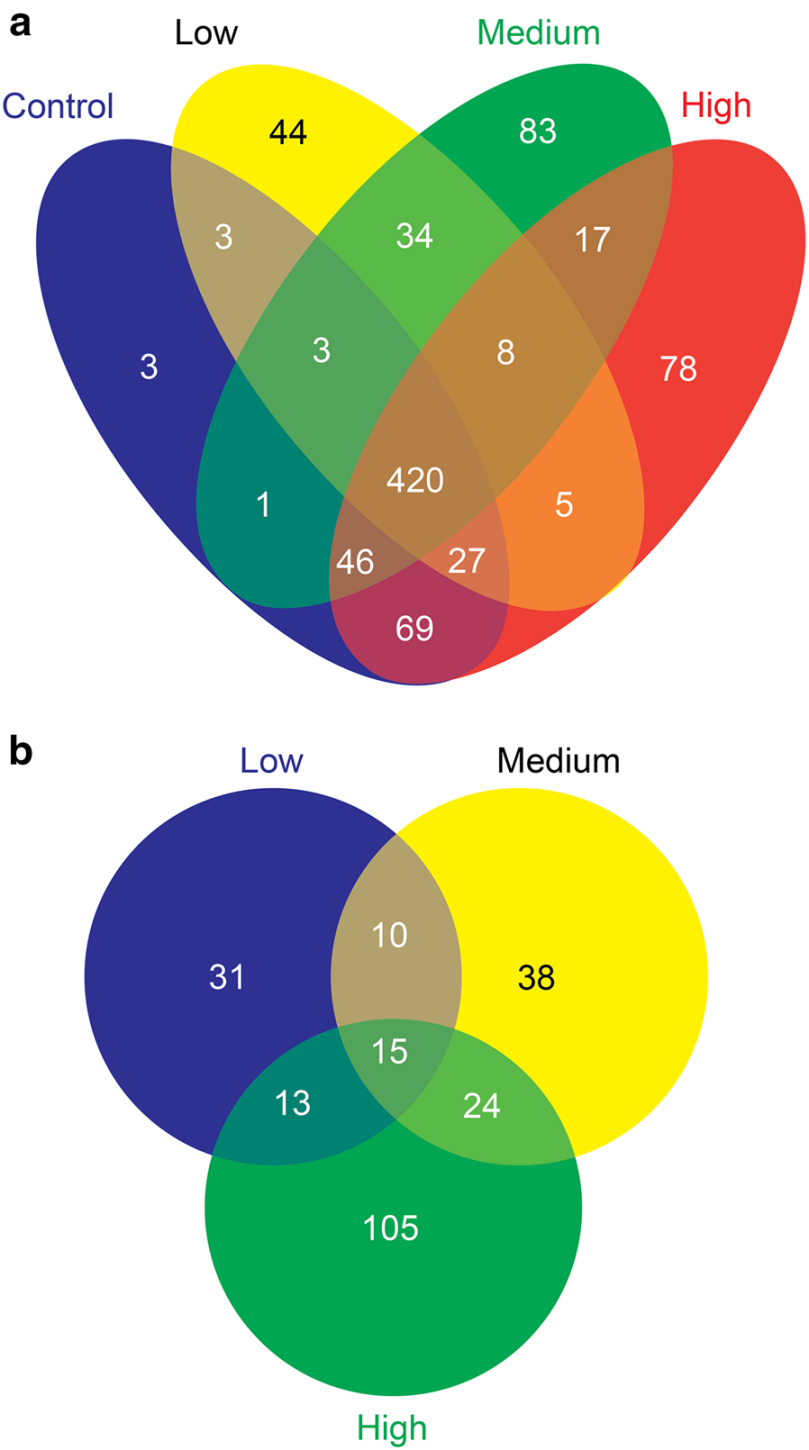
Global protein profile of human seminal plasma. Venn diagram showing, **a** Global proteomic analysis of expressed proteins; **b** distribution of differentially expressed proteins in Low, Medium and High ROS groups

The total spectral counts range for seminal plasma samples ranged from 19591 to 27654. The overall distribution of identified proteins in different groups is shown in Fig. 1. Irrespective of ROS levels and fertility status of the individual, 420 proteins were expressed across the groups while only 3, 44, 83, and 78 proteins were exclusively identified in control, Low, Medium and High ROS groups, respectively (Fig. 1a.).

### Expression profile of differentially expressed proteins

The total number of differentially expressed proteins (DEPs) as per the filtration criteria described in methods section was 236. The number of DEPs that were unique, similar or common to infertile groups with different levels of ROS in comparison to fertile control groups is shown in Fig. 1b. A total of 15 proteins are common to all the three (Low, Medium and High) infertile ROS groups when compared with control. In comparison to control, the total numbers of DEPs found are 69, 87 and 157 in case of Low, Medium and High ROS groups, respectively. Similarly the numbers of unique proteins in these groups with respect to control are 31, 38 and 105, respectively. The Low ROS group has 10 DEPs similar to Medium ROS and 13 DEPs similar to High ROS group while the Medium and High ROS groups have 24 similar DEPs (Fig. 1b).

**Fig. 2:**
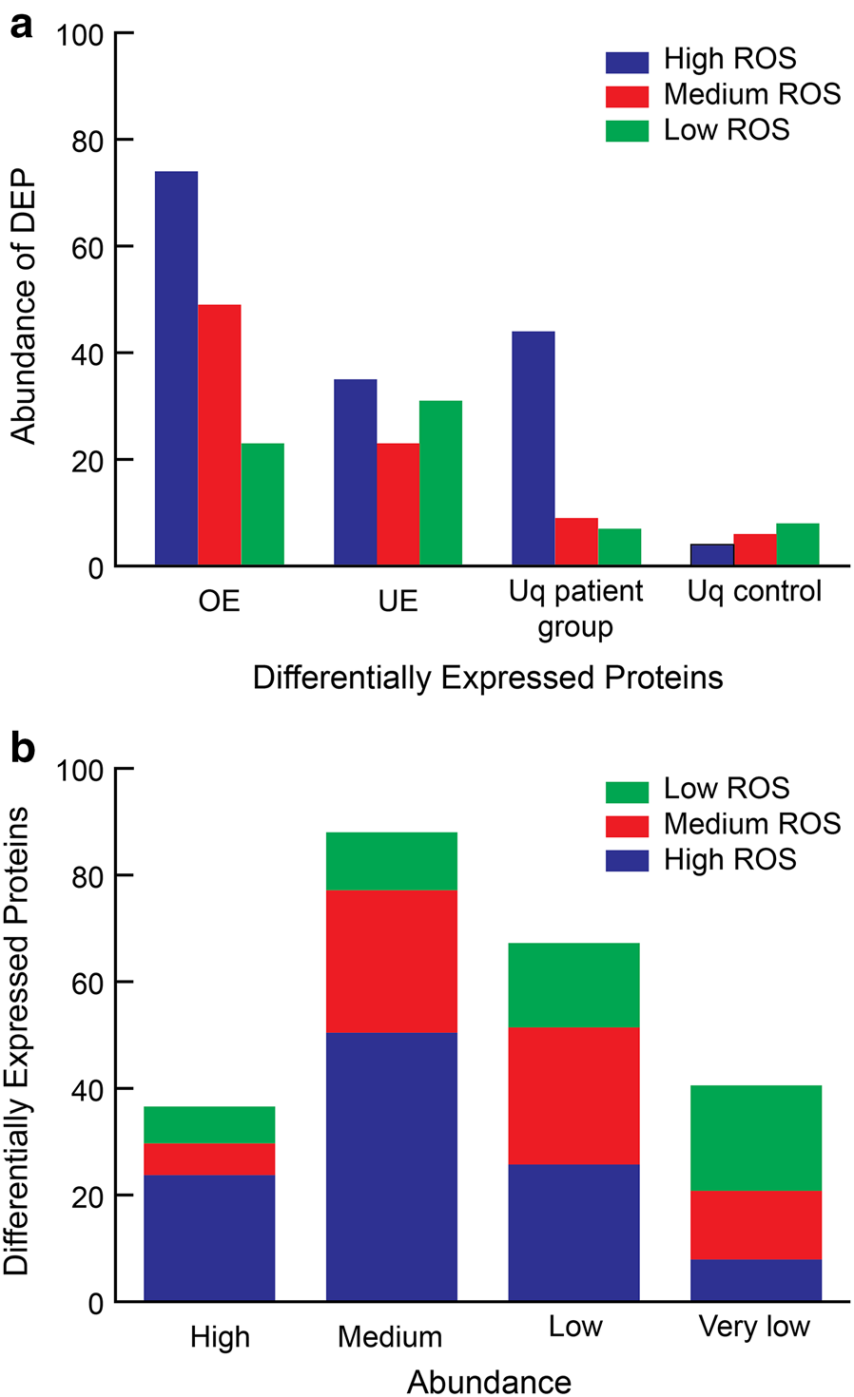
Distribution and abundance of proteins in infertile men with Low, Medium and High ROS levels in comparison with control. **a** Over, under and uniquely expressed proteins; **b** High, Medium, Low and Very Low abundance proteins

The distribution of overexpressed (OE), under expressed (UE) and unique proteins in each of the three categories (i.e., Low, Medium and High ROS) and control group is shown in Fig. 2a. The numbers of proteins overexpressed in infertile group in comparison with the fertile control group were 23, 49 and 74 in Low, Medium and High ROS groups, respectively whereas the numbers of proteins underexpressed were 31, 23 and 35, respectively. Highest number (44) of unique proteins were observed in High ROS group followed by Medium (9) and the smallest for Low (7) ROS group when compared with fertile controls. On the other hand, the control group exhibited 8, 6 and 4 unique proteins in comparison with Low, Medium and High ROS groups respectively. In terms of abundance, maximum numbers of high abundance proteins were identified in High ROS group (24) while in Medium and Low ROS groups 6 and 7 proteins were recorded (Fig. 2b). Similarly, 51 Medium abundance proteins were identified in High ROS group with respect to control followed by Medium (27) and Low (11) ROS group. Both High and Medium ROS groups expressed 26 Low abundance proteins while 16 Low abundance proteins were identified in Low ROS group in comparison with control. Low ROS group exhibited maximum numbers (20) of Very Low abundance proteins in comparison to control followed by Medium ROS group (13) and the list was recorded for High ROS group (8) (Fig. 2b).

### Functional annotations and pathway analysis

**Fig. 3:**
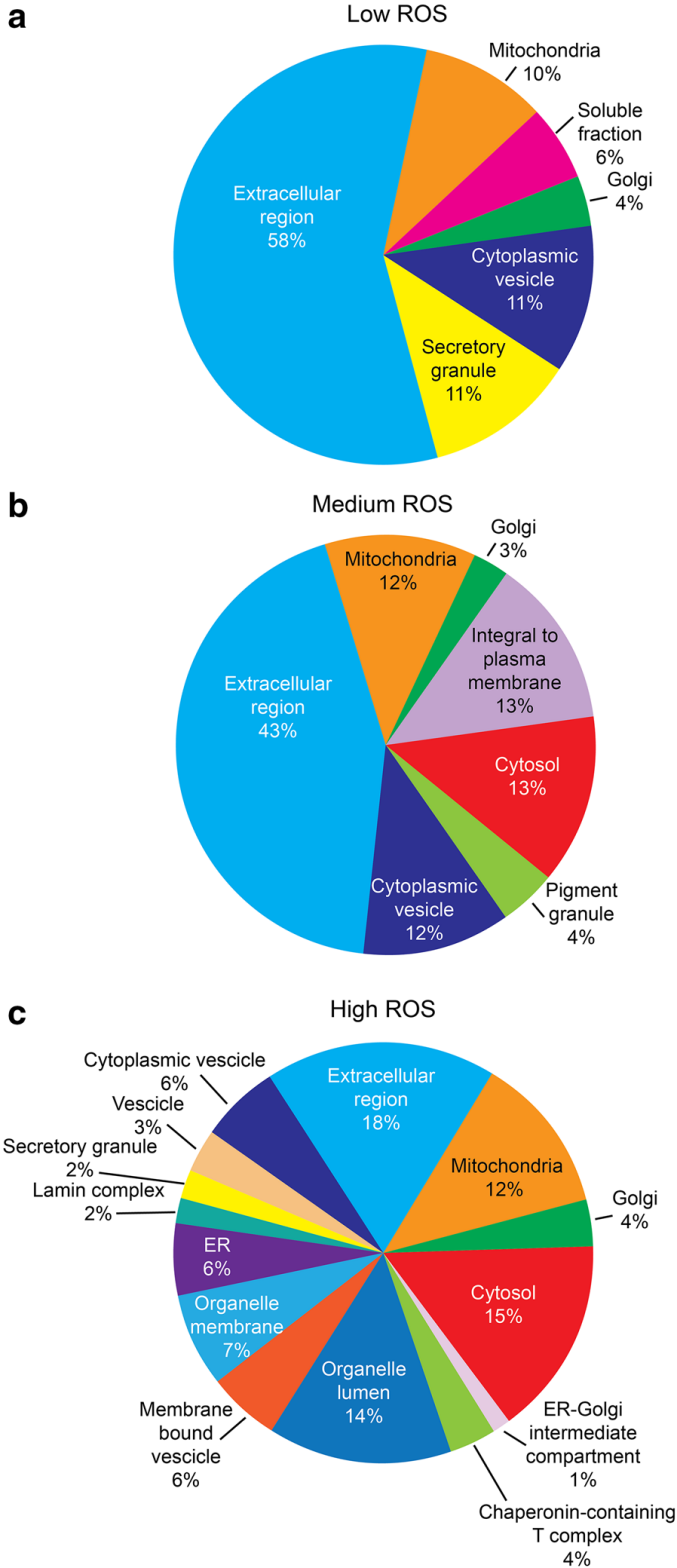
Proteomic signatures of human seminal plasma as a function of ROS gradient. *Pie chart* depicting spatial distribution of differentially expressed proteins DEPs in seminal plasma of infertile groups with respect to fertile group based on GO, STRAP and DAVID functional analysis. **a** Infertile men with Low ROS levels; **b** infertile men with Medium ROS levels; **c** infertile men with High ROS levels

**Fig. 4:**
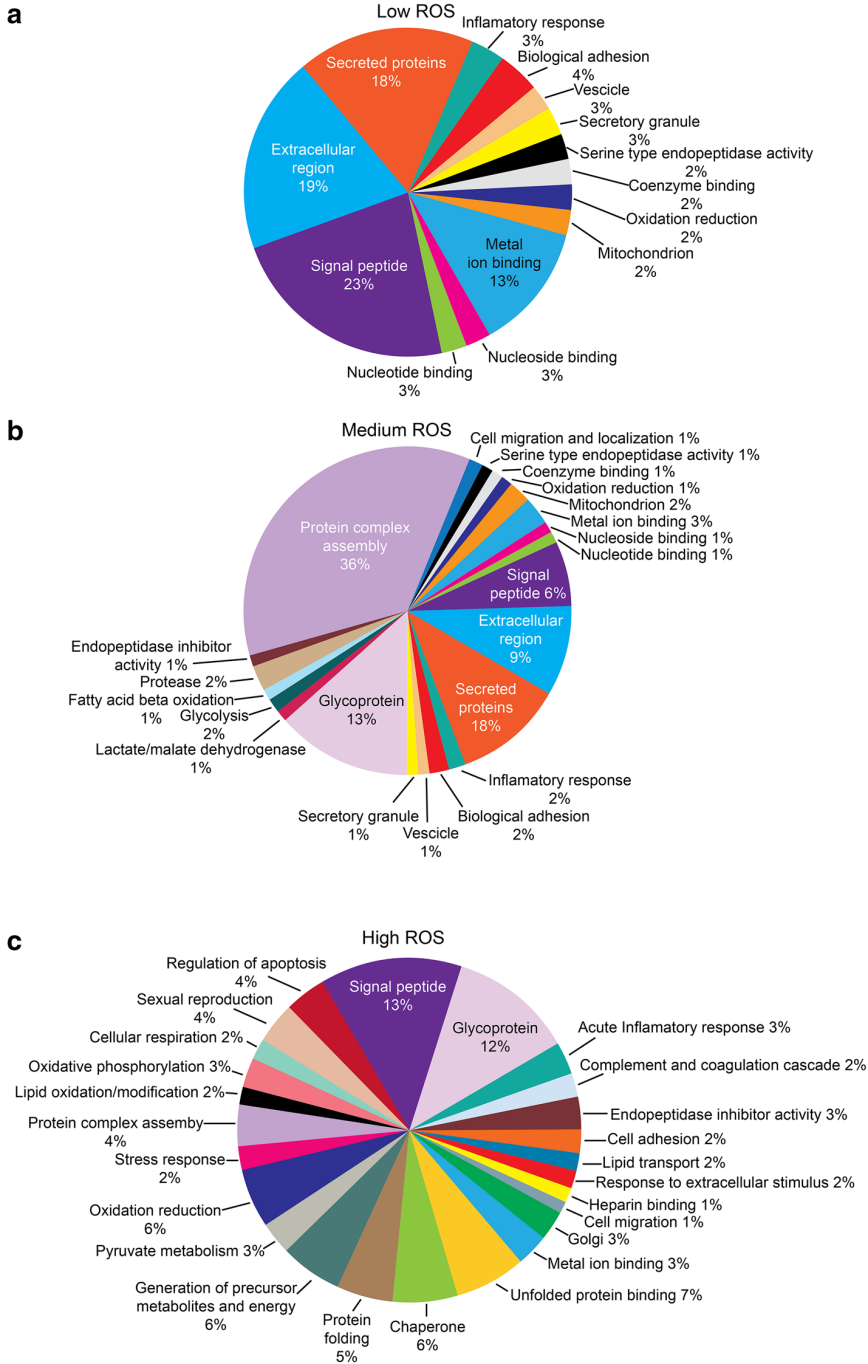
Proteomic signatures of human seminal plasma as a function of ROS gradient. *Pie chart* depicting distribution of differentially expressed proteins (DEPs) involved in different biological processes in seminal plasma of infertile groups with respect to fertile group based on GO, STRAP and DAVID functional analysis. **a** Infertile men with Low ROS levels; **b** Infertile men with Medium ROS levels; **c** infertile men with High ROS levels

Functional annotation and enrichment analysis from consolidated findings using publicly available bioinformatics annotation tools and databases (GO Term Finder, GO Term Mapper, UniProt, STRAP, DAVID) and proprietary software package (Ingenuity Pathway Analysis) revealed a differential expression profile of seminal plasma proteins in comparison to fertile donors (Figs. 3, 4). Seminal plasma is principally composed of secretory proteins, various peptides and proteins in membranous vesicles. With increase in the levels of ROS, a gradual increase in proteins of different cellular origin was found (Fig. 3). Proteins belonging to extracellular regions showed a gradual decline across the ROS gradient in comparison to fertile donors (Fig. 3). In Low ROS group, most of the proteins belonged to extracellular region, secretory granule, cytoplasmic vesicles, mitochondria, soluble fraction and Golgi body (Fig. 3a). In Medium ROS group, proteins integral to plasma membrane, mitochondria, cytosol and pigment granules were reported (Fig. 3b). In contrast, in High ROS group many proteins belonging to endoplasmic reticulum-Golgi intermediate compartment, organelle membrane, mitochondria, organelle lumen, cytoplasmic vesicles and membrane bound vesicles were found (Fig. 3c).

**Fig. 5:**
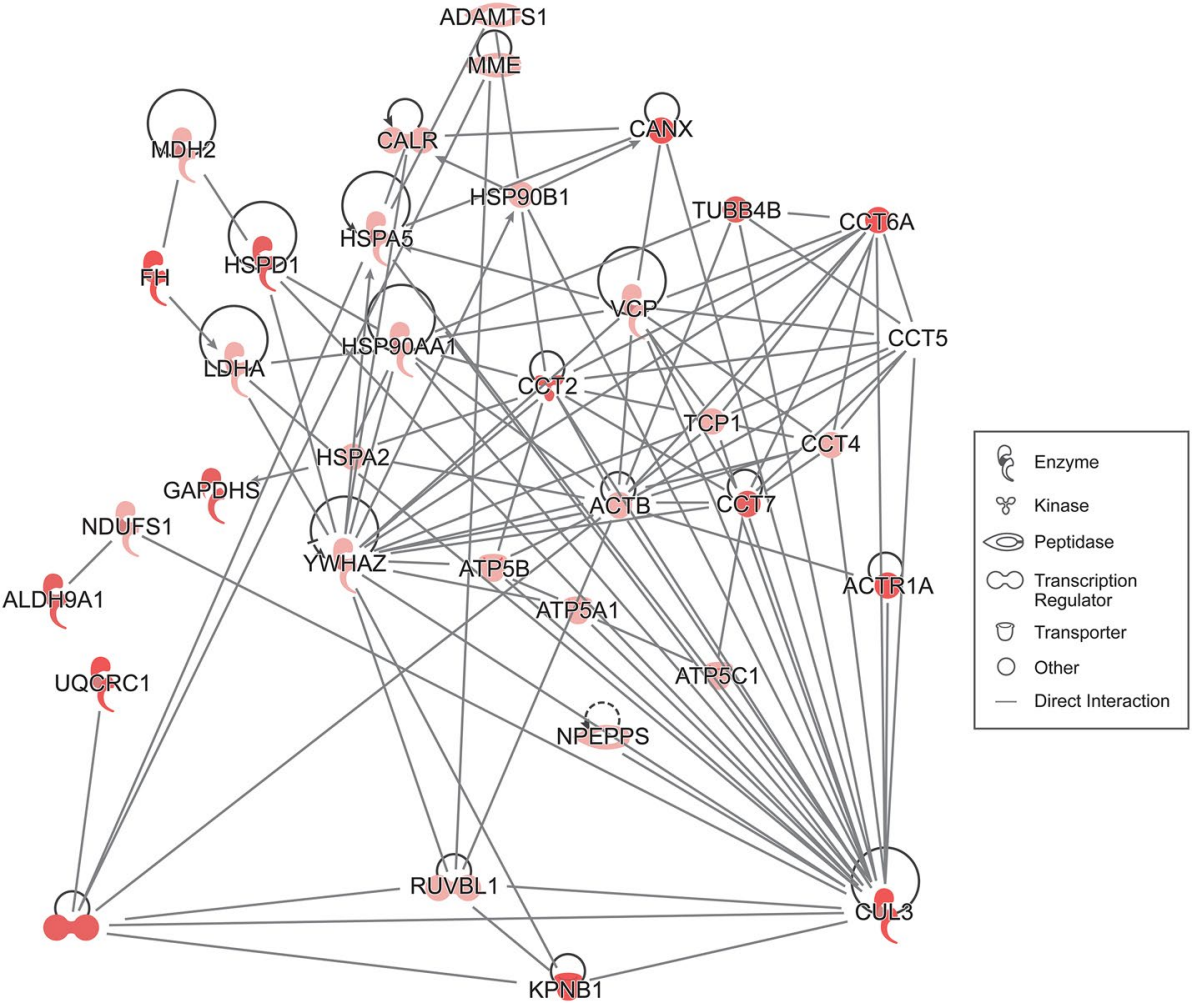
IPA generated network for over expressed seminal plasma proteins in infertile men with High ROS levels. All the 35 focus molecules identified by IPA were overexpressed in the present study. Further analysis revealed that the pathway involved proteins engaged in post-translational modifications, protein folding and developmental disorder. The intensity of color of the focus molecule is proportional to their fold of expression

Enriched functional analysis revealed that in the Low ROS group, majority of DEP were signal peptides, proteins of the extracellular region and secreted proteins (Fig. 4a). On the other hand, proteins involved in metabolic processes were differentially expressed in Medium and High ROS groups in comparison to control (Fig. 4b, c). In general, proteins involved in metabolism and energy production, protein folding and degradation, stress response proteins were activated and those involved in acute inflammatory responses were under expressed (Table 1). It was interesting to note that out of the 15 DEPs shared by three infertile ROS groups (Fig. 1b) only 8 were consistently overexpressed (3) or underexpressed (5) in all the three infertile ROS groups in comparison to the fertile control group (Table 2). Although the mitochondrial precursor of the trifunctional enzyme subunit alpha (HADHA; a mitochondrial matrix enzyme involved in fatty acid metabolism) was overexpressed across all infertile ROS groups, its expression was markedly augmented in High ROS group (~110 fold) in comparison to Low and Medium ROS group). Similarly, the protein FAM3D (Q96BQ1) was uniquely expressed in the fertile control group and not identified in any of the infertile group (Table 2). IPA analysis revealed that all the 35 proteins of a single biological network were overexpressed in High ROS group (Table 3; Fig. 5). A key member of the network, Neprilysin, also known as membrane metallo-endopeptidase (MME) was consistently overexpressed (>2 fold) across the three infertile ROS groups (Table 2). This pathway involved proteins required for protein turnover such as proteases, chaperones, proteins involved in ubiquitination, protein imports into nucleus and mitochondria and complex macromolecular assembly. Besides, it also included mitochondrial proteins for electron transport and ATP synthesis (Fig. 5).

**Table 1:** Differentially expressed proteins in infertile groups with respect fertile controls as revealed by DAVID functional annotations

Functional annotations	Low ROS	Medium ROS	High ROS
Proteins associated with molecular functions	*Overexpressed*: disulfide bond (13), glycoprotein (12), signal peptide (11), hydrolase (9), acetylation (9), EC (7), cytoplasmic vesicle (6), proteolysis (6), secreted (6), peptidase activity (5), mitochondrion (5), endopeptidase activity (4)	*Overexpressed:* signal peptide (21), glycoprotein (21), disulfide bond (16), secreted (17), extracellular region (16), cytoplasmic vesicle (8), mitochondrion (8), proteolysis (7), generation of precursor metabolites and energy (6), peptidase activity (6), fertilization (3), sperm-egg recognition (2), binding of sperm to zona pellucida (2)	*Overexpressed*: acetylation (47), phosphoprotein (41), cytoplasm (31), nucleotide binding (30), cytosol (25), mitochondrion (20), ATP binding (20), unfolded protein binding (16), proteolysis (16), transit peptide (15), generation of precursor metabolites and energy (14), oxidoreductase (13), peptidase activity (13), protein folding (12), sexual reproduction (9), protein complex assembly (9), regulation of apoptosis (9), cell cycle process (7), cellular ion homeostasis (5), protein transport (7), cell cycle (7)
	*Underexpressed*: signal peptide (27), secreted (21), extracellular region (23), disfulfide bond (17), response to wounding (6), hydrolase (6), calcium ion binding (6), peptidase inhibitor activity (5), cell adhesion (5), inflammatory response (4), defense response (4)	*Underexpressed*: signal peptide (17), secreted (12), extracellular region (14), N-glycosylation site (13), disulfide bond (10), growth factor binding (3), pyroglutamic acid (3)	*Underexpressed*: signal peptide (31), glycoprotein (27), acute inflammatory response (7), disulfide bond (20), response to wounding (9), proteinaceous extracellular matrix (7), homeostatic process (5), vesicle (5), cell adhesion (5)
Activated processes/functions	Proteolysis (6), pyruvate metabolic process (2), cell projection assembly (2), acetyl-CoA C-acetyltransferase activity (2), peptidase activity (5), coenzyme binding (3), protein dimerization activity (4), enzyme inhibitor activity (3)	Glycolysis (4), oxidation reduction (7), proteolysis (7), sexual reproduction (5), generation of precursor metabolites and energy (6), peptidase activity (6), fertilization (3), sperm-egg recognition (2), fatty acid metabolic process (4), sperm-egg recognition (2), binding of sperm to zonapellucida (2), peptidase activity (6), acetyltransferase activity (3), lactate dehydrogenase activity (2), hydrolyase activity (3), Ca^2+^-dependent protein binding (2)	Protein folding (12), generation of precursor metabolites and energy (14), oxidation reduction (13), proteolysis (16), gamete generation (6), glycolysis (6), regulation of apoptosis (9), cellular protein complex assembly (7), sexual reproduction (9), gastrulation (3), single fertilization (3), spermatid differentiation (3), sperm egg recognition (2), unfolded protein binding (16), nucleotide binding (30), purine nucleotide binding (26), peptidase activity (13), coenzyme binding (7), serine hydrolase activity (4)
Downregulated processes/functions	Acute inflammatory response (4), response to wounding (6), cell adhesion (5), NO mediated signal transduction (2), protein-lipid complex remodeling (2), defense response (4), activation of plasma proteins in acute inflammatory response (2); peptidase inhibitor activity (5), calcium ion binding (6), structural molecule activity (5)	Acute phase response (2), acute inflammatory response (2), growth factor binding (3)	Acute inflammatory response (7), response to wounding (9), defense response (8), lipid transport and localization (4), homeostatic process (5), complement activation (2), cell adhesion (5), cell morphogenesis involved in differentiation (3), peptidase inhibitor activity (7), glycosaminoglycan binding (3), protease binding (2), structural molecule activity (5)
Activated pathways	Valine, leucine and isoleucine degradation (3), propanoatemetabolism (2), butanoate metabolism (2), tryptophan metabolism (2), pyruvate metabolism (2), fatty acid metabolism (2), lysine degradation (2), mechanism of gene regulation by peroxisome proliferators via peroxisome proliferator-activated receptor alpha (2)	Propanoatemetabolism (4), pyruvate metabolism (4), glycolysis (3), metabolism of lipids and lipoproteins (5), integration of energy metabolism (4), metabolism of carbohydrates (3)	Integration of energy metabolism (11), diabetes pathways (12), metabolism of proteins (10), TCA cycle (4), metabolism of carbohydrates (5), metabolism of amino acids (5), Parkinson’s disease (6), fatty acid elongation in mitochondria (2)
Downregulated pathways	Complement and coagulation cascades (3), lectin induced complement pathway (2)		Complement and coagulation cascades (5), ECM-receptor interaction (4), glycerolipid metabolism (3), hemostasis (4), complement pathway (2)
Processes/Functions unique to each ROS group	Protease inhibitor (2), endopeptidase inhibitor activity (2), signal peptide (4), anchored to membrane (2), fatty acid metabolism (1), cell membrane (3), glycoprotein (3)	Plasma membrane (3), polymorphism (5), cell surface linked signal transduction (1), spermatogenesis (2)	Mitochondrial envelope (14), organelle lumen (16), TCA cycle (3), metal cluster binding (3), mitochondrial respiratory chain (4), carbohydrate catabolic process (3), monosaccharide metabolic process (4), oxidoreductase activity (3), nucleotide binding (12), regulation of apoptosis (4), ATP binding (4), metal ion binding (11), mitochondrion (25), transit peptide (21), oxidation reduction (11), generation of precursor metabolites and energy (12), cellular respiration (8), organelle lumen (16), oxidative phosphorylation (5), TCA cycle (5), metabolism of carbohydrates and amino acids (4), integration of energy metabolism (8), respiratory chain (4), endoplasmic reticulum(4)
Processes/functions unique to each control group	Secreted (5), signal peptide (5), glycoprotein (5), extracellular region (4), defense response (1), inflammatory response (1)	Signal peptide (5), N-linked glycosylation (4), metal ion binding (3), desmosome (2), cell adhesion (1)	Signal peptide (3), glycoprotein (3), anchoring junction (2), cell–cell junction assembly (1), homophilic cell adhesion (1), negative regulation of peptide secretion (1)
Functional categories related to reproduction/spermatogenesis			
Overexpressed	–	Sexual reproduction (5), fertilization (3), sperm-egg recognition (2), sperm-egg recognition (2), binding of sperm to zona pellucida (2)	Sexual reproduction (9), reproductive cellular process (5), spermatid development/differentiation (3), gamete generation (6), spermatogenesis (5), germ cell development (3), reproductive process in a multicellular organism (6), reproductive developmental process (4)
Underexpressed	**–**	Spermatogenesis (2), sperm motility, fertilization, sexual reproduction (1)	

Numbers in parentheses represent numbers of proteins identified

**Table 2:** Proteins that are uniformly under- or overexpressed in infertile ROS groups in comparison to fertile controls

Name of the gene	Name of the protein	NSAF ratio		
		Low ROS	Medium ROS	High ROS
LTBP1	Latent-transforming growth factor beta-binding protein 3 isoform 1 precursor	0.11	0.05	0.33
COL6A2	Collagen alpha-2(VI) chain isoform 2C2 precursor	0.15	0.29	0.22
GUCY1B3	Guanylatecyclase soluble subunit beta-1	0.22	0.03	0.12
GLG1	Golgi apparatus protein 1 isoform 2 precursor	0.31	0.47	0.34
NUCB2	Nucleobinding-2 isoform X1	0.54	0.46	0.6
FLT3	Receptor-type tyrosine-protein phosphatase S isoform X1	2.12	2.49	2.5
MMEL1	Neprilysin isoform X1	2.61	2.8	2.24
HADHA	Trifunctional enzyme subunit alpha, mitochondrial precursor	24.53	21.49	110.04

**Table 3:** Proteins involved in the proposed network as revealed by Ingenuity Pathway Analysis

No.	Uniprot ID	Name	Primary gene name	Function/catalytic activity	NSAF ratio		
					Low ROS	Medium ROS	High ROS
1	P60709	Actin,	ACTB	Actins are highly conserved proteins that are involved in various types of cell motility and are ubiquitously expressed in all eukaryotic cells	1.48	1	2.34
		cytoplasmic 1					
2	P61163	Alpha-centractin	ACTR1A	Component of a multi-subunit complex involved in microtubule based vesicle motility. It is associated with the centrosome	1.82	1	33.08
3	Q9UHI8	A disintegrin and metalloproteinase with thrombospondin motifs 1	ADAMTS1	Cleaves aggrecan, a proteoglycan, and may be involved in its turnover. Has angiogenic inhibitor activity. Active metalloprotease, which may be associated with various inflammatory processes as well as development of cancer	2.28	1.73	2.46
4	P49189	4-trimethylaminobutyraldehyde dehydrogenase	ALDH9A1	Converts gamma-trimethylaminobutyraldehyde into gamma-butyrobetaine. Catalyzes the irreversible oxidation of a broad range of aldehydes to the corresponding acids in an NAD-dependent reaction. Preferential cleavage of polypeptides between hydrophobic residues, particularly with Phe or Tyr at P1′	1	1	11.76
5	P25705	ATP synthase subunit alpha, mitochondrial	ATP5A1	Mitochondrial membrane ATP synthase (F(1)F(0) ATP synthase or Complex V) produces ATP from ADP in the presence of a proton gradient across the membrane which is generated by electron transport complexes of the respiratory chain	2	3.53	9.55
6	P06576	ATP synthase subunit beta, mitochondrial	ATP5B	Mitochondrial membrane ATP synthase (F(1)F(0) ATP synthase or Complex V) produces ATP from ADP in the presence of a proton gradient across the membrane which is generated by electron transport complexes of the respiratory chain	2.79	2.59	9.59
7	P36542	ATP synthase subunit gamma, mitochondrial	ATP5C1	Mitochondrial membrane ATP synthase (F(1)F(0) ATP synthase or Complex V) produces ATP from ADP in the presence of a proton gradient across the membrane which is generated by electron transport complexes of the respiratory chain	1	2.12	4.83
8	P27797	Calreticulin	CALR	Calcium-binding chaperone that promotes folding, oligomeric assembly and quality control in the endoplasmic reticulum (ER) via the calreticulin/calnexin cycle. This lectin interacts transiently with almost all of the monoglucosylated glycoproteins and cleaves aggrecan at the 1938-Glu-|-Leu-1939 site, within the chondroitin sulfate attachment domain	0.94	0.94	3
9	P27824	Calnexin	CANX	Calcium-binding protein that interacts with newly synthesized glycoproteins in the endoplasmic reticulum. It may act in assisting protein assembly and/or in the retention within the ER of unassembled protein subunits. It seems to play a major role in the ATP + H(2)O = ADP + phosphate	1	1	53.77
10	P78371	T-complex protein 1 subunit beta	CCT2	Molecular chaperone; assists the folding of proteins upon ATP hydrolysis. As part of the BBS/CCT complex may play a role in the assembly of BBSome, a complex involved in ciliogenesis regulating transports vesicles to the cilia. Known to play a role in vitro	1.41	1.58	11.35
11	P50991	T-complex protein 1 subunit delta	CCT4	Molecular chaperone; assists the folding of proteins upon ATP hydrolysis. As part of the BBS/CCT complex may play a role in the assembly of BBSome, a complex involved in ciliogenesis regulating transports vesicles to the cilia	0.37	1.43	3.39
12	P48643	T-complex protein 1 subunit epsilon	CCT5	Molecular chaperone; assists the folding of proteins upon ATP hydrolysis. As part of the BBS/CCT complex may play a role in the assembly of BBSome, a complex involved in ciliogenesis regulating transports vesicles to the cilia. Release of an N-terminal amino acid, preferentially alanine, from a wide range of peptides, amides and arylamides	0.57	1.39	10.45
13	P40227	T-complex protein 1 subunit zeta	CCT6A	Molecular chaperone; assists the folding of proteins upon ATP hydrolysis. Known to play a role, in vitro in the folding of actin and tubulin	1	1	46.53
14	Q99832	T-complex protein 1 subunit eta	CCT7	Molecular chaperone; assists the folding of proteins upon ATP hydrolysis. Known to play a role in vitro in the folding of actin and tubulin	2.26	2.48	8.1
15	Q13618	Cullin-3	CUL3	Core component of multiple cullin-RING-based BCR (BTB-CUL3-RBX1) E3 ubiquitin-protein ligase complexes which mediate the ubiquitination and subsequent proteasomal degradation of target proteins. As a scaffold protein may contribute to catalysis through positioning of the substrate and the ubiquitin-conjugating enzyme, involved in ER-Golgi transport by regulating the size of COPII coats, thereby playing a key role in collagen export, which is required for embryonic stem (ES) cells division	1	11.18	77.7
16	P07954	Fumaratehydratase, mitochondrial	FH	Also acts as a tumor suppressor	21.75	29.87	153.16
				NADH + acceptor = NAD(+) + reduced acceptor			
17	O14556	Glyceraldehyde-3-phosphate dehydrogenase, testis-specific	GAPDHS	May play an important role in regulating the switch between different pathways for energy production during spermiogenesis and in the spermatozoon. Required for sperm motility and male fertility	1	1.44	10.4
				ATP + H(2)O = ADP + phosphate			
18	P07900	Heat shock protein HSP 90-alpha	HSP90AA1	Molecular chaperone that promotes the maturation, structural maintenance and proper regulation of specific target proteins involved for instance in cell cycle control and signal transduction. Undergoes a functional cycle that is linked to its ATPase activity	1.58	1	2.24
19	P14625	Endoplasmin	HSP90B1	Molecular chaperone that functions in the processing and transport of secreted proteins. When associated with CNPY3, required for proper folding of Toll-like receptors. Functions in endoplasmic reticulum associated degradation (ERAD)	1	1.54	3.02
20	P54652	Heat shock-related 70 kDa protein 2	HSPA2	In co-operation with other chaperones, Hsp70 stabilize pre-existent proteins against aggregation and mediate the folding of newly translated polypeptides in the cytosol as well as within organelles	1.43	1	1.93
21	P11021	78 kDa glucose-regulated protein	HSPA5	Probably plays a role in facilitating the assembly of multimeric protein complexes inside the endoplasmic reticulum. Involved in the correct folding of proteins and degradation of misfolded proteins via its interaction with DNAJC10, probably to facilitate the release of DNAJC10 from its substrate	0.94	1	3.3
22	P10809	60 kDa heat shock protein, mitochondrial	HSPD1	Implicated in mitochondrial protein import and macromolecular assembly. May facilitate the correct folding of imported proteins. May also prevent misfolding and promote the refolding and proper assembly of unfolded polypeptides generated under stress condition	1.81	1	7.75
23	P52292	Importin subunit alpha-1	KPNA2	Functions in nuclear protein import as an adapter protein for nuclear receptor KPNB1. Binds specifically and directly to substrates containing either a simple or bipartite nuclear localization signal motif. Docking of the importin/substrate complex to the nuclear pore complex (NPC) is mediated by KPNB1 through binding to nucleoporin FxFG repeats and the complex is subsequently translocated through the pore by an energy requiring, Ran-dependent mechanism. At the nucleoplasmic side of the NPC, Ran binds to importin-beta and the three components separate and importin-alpha and -beta are re-exported from the nucleus to the cytoplasm where GTP hydrolysis releases Ran from importin. The directionality of nuclear import is thought to be conferred by an asymmetric distribution of the GTP- and GDP-bound forms of Ran between the cytoplasm and nucleus	1	1	12.34
24	P00338	L-lactate dehydrogenase A chain	LDHA	(S)-lactate + NAD(+) = pyruvate + NADH	2.02	2.16	2.28
25	P40926	Malate dehydrogenase, mitochondrial	MDH2	(S)-malate + NAD^+^ = oxaloacetate + NAD	1.09	7.08	8.62
26	P08473	Neprilysin	MME	Metalloprotease involved in sperm function, possibly by modulating the processes of fertilization and early embryonic development. Degrades a broad variety of small peptides with a preference for peptides shorter than 3 kDa containing neutral bulky aliphatic or aromatic amino acid residues	2.61	2.8	2.24
27	P28331	NADH-ubiquinone oxidoreductase 75 kDa subunit, mitochondrial	NDUFS1	Core subunit of the mitochondrial membrane respiratory chain NADH dehydrogenase (Complex I) that is believed to belong to the minimal assembly required for catalysis. Complex I functions in the transfer of electrons from NADH to the respiratory chain	2.44	1	4.11
28	P55786	Puromycin-sensitive aminopeptidase	NPEPPS	Aminopeptidase with broad substrate specificity for several peptides. Involved in proteolytic events essential for cell growth and viability. May act as regulator of neuropeptide activity. Plays a role in the antigen-processing pathway for MHC class I molecule 4-aminobutanal + NAD^+^ + H_2_O = 4-aminobutanoate + NADH	1.53	0.67	3.19
29	Q9Y265	RuvB-like 1	RUVBL1	Possesses single-stranded DNA-stimulated ATPase and ATP-dependent DNA helicase (5′ to 3′) activity. Component of a SWR1-like complex that specifically mediates the removal of histone H2AZ/H2AFZ from the nucleosome	0.3	0.67	2.98
30	Q9Y230	RuvB-like 2	RUVBL2	Possesses single-stranded DNA-stimulated ATPase and ATP-dependent DNA helicase (5′ to 3′) activity. Component of a SWR1-like complex that specifically mediates the removal of histone H2A.Z/H2AFZ from the nucleosome	1	0.75	13.69
31	P17987	T-complex protein 1 subunit alpha	TCP1	Molecular chaperone; assists the folding of proteins upon ATP hydrolysis. As part of the BBS/CCT complex may play a role in the assembly of BBSome, a complex involved in ciliogenesis regulating transports vesicles to the cilia. Known to play a role in folding of actin and tubulin	1.65	1.6	3.96
32	P68371	Tubulin beta-4B chain	TUBB4B	Tubulin is the major constituent of microtubules. It binds two moles of GTP, one at an exchangeable site on the beta chain and one at a non-exchangeable site on the alpha chain	1.02	1.85	6.12
33	P31930	Cytochrome b-c1 complex subunit 1, mitochondrial	UQCRC1	This is a component of the ubiquinol-cytochrome c reductase complex (complex III or cytochrome b-c1 complex), which is part of the mitochondrial respiratory chain. This protein may mediate formation of the complex between cytochromes c and c1	1	1	21.68
34	P55072	Transitional endoplasmic reticulum ATPase	VCP	Necessary for the fragmentation of Golgi stacks during mitosis and for their reassembly after mitosis. Involved in the formation of the transitional endoplasmic reticulum (tER). The transfer of membranes from the endoplasmic reticulum to the Golgi apparatus	1.06	1.65	4.15
35	P63104	14-3-3 protein zeta/delta	YWHAZ	Adapter protein implicated in the regulation of a large spectrum of both general and specialized signaling pathways. Binds to a large number of partners, usually by recognition of a phosphoserine or phosphothreonine motif. Binding generally results in the(S)-malate = fumarate + H_2_O	1.22	0.99	2.12

## Discussion

The importance of seminal plasma is most often undermined and restricted to transport and protection of spermatozoa until fertilization. Possible pathways by which seminal fluid is involved in eliciting the paternal effect includes post-ejaculatory effects on spermatozoa and regulation of various female events that impact embryo development [3]. In the current investigation we report the differential expression of seminal plasma proteins as a function of ROS levels in infertile men in comparison with fertile donors. Seminal plasma proteome is as complex as that of blood plasma [43, 44]. In the present study, comparative proteomic analysis revealed a network of protein associated with energy metabolism and protein turnover leading to reproductive dysfunction.

Global proteomic analysis revealed majority of proteins with respect to cellular distribution to be proteins of the cytoplasmic vesicles, secretory granules, soluble fraction, extracellular region, organelle membranes and membrane bound vesicles. In addition to sperm cells and soluble molecules, mammalian semen contains a variety of membranous vesicles such as prostasomes and epididymosomes, which are derived from prostatic and epididymal epithelial cells, respectively. Other tissues within the male genital tract, such as the vesicular glands, which produce the majority of seminal fluid, and the vasa deferentia, may also contribute membrane vesicles to the seminal plasma [45]. Epididymosomes are known to transfer proteins from the epididymal epithelium to passing sperm cells [46]. Many different processes have been attributed to prostasomes, including stimulation of sperm cell motility through delivery of Ca^2+^ signaling tools [47] and either inhibition [48] or stimulation [49] of the acrosome reaction. Prostasomes could also help to protect sperm cells from immune responses within the female reproductive tract by inhibiting the complement pathway [50], inhibiting lymphocyte proliferation [51], and inhibiting monocyte and neutrophil phagocytosis [52], possibly via contained immunomodulatory proteins such as galectin 3 [53] or CD48 [54]. However, with augmented levels of ROS, more organelle fragments, mitochondrial proteins and ER-Golgi intermediates were observed in the seminal plasma in comparison to control suggesting ROS mediated cell death. In fact, a significant enhancement in the expression of proteins associated with mitochondrial electron transport chain and ATP synthesis was noticed with increasing levels of ROS in seminal plasma. Since ~2 % of oxygen used by mitochondrial electron transport system is incompletely reduced to ROS particularly at the site of complex-I and -III, an enhanced activity of NDUFS1 (NADH-ubiquinone oxidoreductase 75 kDa subunit, complex-I) as reported in the present study would lead to further augmentation of ROS. Since proteins involved in acute inflammatory response are underexpressed and since all samples used in the study were leukocyte negative, it is suggested that the enhanced production of ROS in Medium and High ROS infertile group could be due to defective sperm physiology or as a consequence of systemic disease process or stress. On the other hand, the infertile group with Low ROS levels exhibited a under expression in stress response proteins. Therefore, despite low or comparable levels of ROS with fertile donors these men remain infertile. A marked increase (~110 fold) in HADHA, mitochondrial precursor (a mitochondrial matrix enzyme) in High ROS group (Table 2) along with complex-I and proteins of ATP synthase complex (inner mitochondrial membrane proteins) further establishes the fact that there is cell death and release of mitochondrial membrane fragments into seminal plasma.

IPA analysis revealed the altered pathways involved in various functional processes. Of all the identified pathways, the one involved in protein folding, post-translational modifications and developmental disorder was the most prominent one as all the 35 focus proteins were identified in our data set and over expressed in all three categories of infertile groups (Fig. 5). These proteins include proteases, chaperones, proteins involved in ubiquitination, protein imports into nucleus and mitochondria and complex macromolecular assembly along with mitochondrial electron chain proteins and ATP synthase.

Mammalian heat shock proteins (HSPs) are molecular chaperones classified according to their molecular weight into several families and named with a suffix of number denoting their molecular weight such as HSPH (HSP110), HSPC (HSP90), HSPA (HSP70), DNAJ (HSP40), HSPB (small HSPs, sHSPs), and two chaperone in families, namely HSPD/E (HSP60/HSP10) and CCT (TRiC) [55]. These HSP families are either inducible by stress (e.g. HSPA1), constitutively expressed, or both (e.g. HSPH1, HSPA8, HSP90AA1). Expression of some HSPs is developmentally regulated or restricted to specific cells [56]. Therefore, a marked over expression in these proteins are not esoteric in infertile men with varied levels of ROS and the High ROS group expressing maximum number of these proteins in terms of fold and abundance. In fact, overexpression of 5 HSPs and 5 CCTs is noticed in High ROS infertile group (Table 3) whereas only one HSP each, HSPA1L and HSP90AA1 was induced in Medium and Low ROS group respectively.

Besides HSPs, proteases play an important role in protein turn over. In this study, an augmentation in various proteases was noticed with respect to ROS levels particularly ADAMTS1 and MME. Most of ADAMs are membrane-anchored glycoproteins that are comprised of a pro-domain, a metalloprotease-like domain, a disintegrin domain, a cysteine-rich region, an epidermal growth factor repeat, a transmembrane region, and a cytoplasmic domain. Fertilin, the first ADAM described, has been implicated in integrin-mediated sperm-egg binding [57]. Instead of the transmembrane region, ADAMTS-1 (ADAM metallopeptidase with thrombospondin type 1 motif, 1) has three thrombospondin (TSP) type I motifs. These are found in both thrombospondins 1 and 2 [58]. These TSP type I motifs of ADAMTS-1 are functional for binding to heparin. Kuno and Matsushima [59] have found that ADAMTS-1 is secreted and incorporated into the extracellular matrix (ECM). ADAMTS-1 cleaves the hyalectan (hyaluronan-binding proteoglycan) aggrecan between a glutamate in the P1 pocket and small aliphatic residues in P1′. ADAMTS-1-null mice display several developmental abnormalities, primarily within the urogenital systems, affecting normal growth, organ morphology and function, and female fertility [60, 61]. A role for ADAMTS-1 in ovulation has been inferred from studies in rats [62], mice [63] and horses [64], which indicate that upregulation of ADAMTS-1 mRNA correlates temporally with the appearance of ADAMTS cleaved versican within the ECM of the cumulus oocyte complex [65]. It has been previously shown that the heparin in seminal fluid stimulates sperm capacitation in bulls. Interestingly, induction of sperm capacitation in the female reproductive tract is aided by heparin binding proteins secreted by the male accessory sex glands [66]. Seminal fluid heparin binding proteins are supposed to attach themselves to the sperm surface, especially lipids containing the phosphoryl-choline group, thus allowing heparin-like GAGs in the female reproductive tract to activate sperm capacitation [67]. Nevertheless, an over expression of ADAMTS1 will lead to excessive cleavage of heparin, rapid liquefaction of semen thereby affecting spermatozoon survival and the overall fertilization process, and this may be directly related to infertility in ROS groups.

Membrane metallo-endopeptidase (MME), also known as Neprilysin, neutral endopeptidase (NEP), cluster of differentiation 10 (CD10), and common acute lymphoblastic leukemia antigen (CALLA) is an enzyme that in humans is encoded by the MME gene. Neprilysin is a zinc-dependent metalloprotease that cleaves peptides at the amino side of hydrophobic residues and inactivates several peptide hormones including glucagon, enkephalins, substance P, neurotensin, oxytocin, and bradykinin. MMEs are essential for development and reproduction in mammals. The activities of MME in seminal fractions are very high in comparison to other tissues (10- to 20-fold higher than in the brain) [68]. Furthermore, the activity of these enzymes is altered in men with asthenozoospermia or necrozoospermia [9]. Subiran et al. [69] have reported the presence of MME in the prostasomes and in the neck region of few spermatozoa. However, they observed that addition of thiorphan (analogue of encephalin that inhibits MME) maintained sperm motility at 2 h, but this effect was not reversed by naloxone which displaces enkephalins from their receptors. This may suggest that MME regulates sperm motility by a mechanism that does not implicate the opioid system. They opined that MME could be involved in the degradation of other peptides, such as bradykinins or tachykinins, which are present in seminal fluid [70, 71]. In this respect, it is interesting to note that in experiments performed with bull spermatozoa, addition of phosphoramidon, another MME inhibitor, produced an increase in sperm motility after 2 h of incubation, presumably by inhibition of bradykinin degradation [70]. However, in the present study, we observed a ~2 fold increase in MME in the infertile group suggesting its role in other mechanism rather than sperm motility alone since the concentration, morphology and motility was impaired in the High ROS group in comparison to fertile controls [27]. Therefore, an in depth study involving various regulators of MME activity in the seminal plasma from infertile patients with High ROS levels as well as its activity in seminal plasma from fertile men after induction of oxidative stress in vitro may shed more light on the mechanism of MME action in seminal plasma and its impact on sperm function. Since both ADAMST1 and MME inhibit angiogenesis, their enhanced activity which is principally from the prostasomes may adversely affect embryo implantation.

Alternatively, both the enzymes are cited upstream to the proteins involved in post-translational modification, protein folding and developmental disorder as revealed by IPA. Therefore, they may be responsible for altering the conformation and subsequent change in activities of key proteins having reproductive function leading to infertility. Furthermore, a secretory protein that belongs to family with sequence similarity 3, member D (FAM3D) is uniquely expressed in fertile donors and absent in all the three infertile groups. Although not much is known about the function of this protein, it is predominantly found in placenta and it belongs to cytokine family of proteins [72]. Therefore, its role in modulating post-ejaculatory event in female reproductive tract for tolerance cannot be ruled out. Since MME is uniformly overexpressed in all the three infertile groups and FAM3D is uniquely expressed in fertile groups, it is suggested that measurement of ROS along with MME and FAM3D levels will be better markers for evaluating fertility status since a cohort of infertile patients (Low ROS group) also exhibit ROS levels comparable to fertile donors.

## Conclusions

In conclusion, we have for the first time demonstrated poor sperm quality that is associated with elevated oxidative stress levels may be associated with altered protein profile of seminal plasma. Since seminal plasma is established as a vehicle for carrying proteins to the spermatozoa during post-testicular maturation phase and has a role in protection and regulation of sperm function and induction of female reproductive tract for successful fertilization and embryo implantation, altered protein expression in response to elevated ROS may impair sperm function. Further validation of DEPs is necessary to establish the role of these proteins as biomarkers of oxidative stress-induced male factor infertility.

## Additional files

Additional file 1:
**Table S1.** Identified proteins in seminal plasma of fertile men Additional file 2:
**Table S2.** Identified proteins in seminal plasma of infertile men with Low ROS levels Additional file 3:
**Table S3.** Identified proteins in seminal plasma of infertile men with Medium ROS levels Additional file 4:
**Table S4.** Identified proteins in seminal plasma of infertile men with High ROS levels
