# Supplementary material for: Comparative proteomic network signatures in seminal plasma of infertile men as a function of reactive oxygen species
Source: Clin Proteomics. 2015 Aug 28;12(1):23. doi: 10.1186/s12014-015-9094-5 (PMC4552280; doi:10.1186/s12014-015-9094-5)
Supplement: Additional file 2: — Table S2. Identified proteins in seminal plasma of infertile men with Low ROS levels [file 12014_2015_9094_MOESM2_ESM.docx]

| **Table S2. Identified proteins in seminal plasma of infertile men with Low ROS level** | | | | | | | | | | | |
| --- | --- | --- | --- | --- | --- | --- | --- | --- | --- | --- | --- |
| **Protein** | **Accession #** | **MW** |  | **Replicate 1** |  | **Replicate 2** |  |  |  | **Replicate 3** |  |
|  |  | **kDa** | Peptide | Coverage | Spectral count | Peptide | Coverage | Spectral count | Peptide | Coverage | Spectral count |
| fibronectin isoform 1 preproprotein | 47132557 | 272 | 108 | 50% | 922 | 125 | 60% | 988 | 120 | 51% | 999 |
| serum albumin preproprotein | 4502027 | 69 | 93 | 82% | 1684 | 87 | 83% | 1672 | 101 | 81% | 1523 |
| lactotransferrin isoform 1 precursor | 54607120 | 78 | 91 | 83% | 1524 | 91 | 84% | 1605 | 91 | 84% | 1439 |
| prostatic acid phosphatase isoform PAP precursor | 6382064 | 45 | 68 | 73% | 1140 | 62 | 70% | 1633 | 76 | 77% | 1250 |
| aminopeptidase N isoform X1 | 530407092 | 110 | 50 | 50% | 337 | 53 | 57% | 357 | 50 | 51% | 363 |
| mucin-6 precursor | 151301154 | 257 | 49 | 25% | 504 | 55 | 27% | 544 | 55 | 26% | 542 |
| serotransferrin precursor | 4557871 | 77 | 43 | 71% | 260 | 42 | 69% | 288 | 46 | 72% | 290 |
| prostate-specific antigen isoform 1 preproprotein | 4502173 | 29 | 40 | 85% | 913 | 44 | 87% | 1009 | 43 | 86% | 992 |
| semenogelin-2 precursor | 4506885 | 65 | 39 | 48% | 694 | 44 | 48% | 510 | 53 | 52% | 587 |
| laminin subunit alpha-5 precursor | 21264602 | 400 | 37 | 13% | 95 | 40 | 14% | 99 | 53 | 19% | 134 |
| zinc-alpha-2-glycoprotein precursor | 4502337 | 34 | 36 | 66% | 366 | 33 | 66% | 427 | 36 | 66% | 388 |
| laminin subunit beta-2 isoform X1 | 530372442 | 196 | 31 | 17% | 106 | 31 | 18% | 119 | 31 | 21% | 127 |
| prosaposin isoform a preproprotein | 11386147 | 58 | 29 | 50% | 192 | 26 | 59% | 212 | 28 | 56% | 212 |
| angiotensin-converting enzyme isoform 1 precursor | 4503273 | 150 | 28 | 25% | 82 | 37 | 25% | 110 | 39 | 27% | 125 |
| clusterinpreproprotein | 355594753 | 52 | 28 | 43% | 345 | 28 | 46% | 370 | 30 | 45% | 374 |
| alpha-1-antitrypsin precursor | 189163532 | 47 | 26 | 58% | 193 | 32 | 63% | 236 | 30 | 57% | 187 |
| dipeptidyl peptidase 4 | 18765694 | 88 | 26 | 32% | 92 | 27 | 35% | 119 | 25 | 30% | 111 |
| neprilysin isoform X1 | 578807443 | 86 | 26 | 51% | 94 | 25 | 46% | 97 | 26 | 45% | 96 |
| plasma serine protease inhibitor preproprotein | 194018472 | 46 | 26 | 67% | 224 | 23 | 63% | 246 | 24 | 67% | 244 |
| sulfhydryl oxidase 1 isoform a precursor | 13325075 | 83 | 26 | 40% | 97 | 23 | 40% | 104 | 24 | 38% | 98 |
| extracellular matrix protein 1 isoform 1 precursor | 221316614 | 61 | 25 | 61% | 160 | 28 | 60% | 202 | 25 | 59% | 176 |
| semenogelin-1 preproprotein | 4506883 | 52 | 24 | 59% | 252 | 30 | 62% | 232 | 31 | 65% | 272 |
| heat shock protein HSP 90-alpha isoform 1 | 153792590 | 98 | 24 | 34% | 153 | 28 | 35% | 138 | 28 | 38% | 127 |
| agrin precursor | 54873613 | 215 | 24 | 10% | 57 | 20 | 8.70% | 47 | 18 | 8.70% | 46 |
| heat shock-related 70 protein 2 | 13676857 | 70 | 23 | 42% | 97 | 20 | 38% | 97 | 21 | 41% | 98 |
| beta-hexosaminidase subunit beta preproprotein | 4504373 | 63 | 23 | 43% | 77 | 18 | 41% | 66 | 18 | 37% | 75 |
| prolactin-inducible protein precursor | 4505821 | 17 | 22 | 77% | 1562 | 33 | 77% | 1468 | 28 | 77% | 1477 |
| isocitrate dehydrogenase [NADP] cytoplasmic | 538917681 | 47 | 20 | 52% | 77 | 21 | 59% | 112 | 21 | 63% | 97 |
| laminin subunit gamma-1 precursor | 145309326 | 178 | 20 | 18% | 59 | 18 | 15% | 54 | 18 | 16% | 57 |
| plastin-2 isoform X2 | 530402335 | 70 | 19 | 41% | 51 | 22 | 57% | 69 | 25 | 59% | 80 |
| galectin-3-binding protein precursor | 5031863 | 65 | 19 | 34% | 172 | 21 | 31% | 182 | 19 | 31% | 163 |
| IgGFc-binding protein precursor | 154146262 | 572 | 19 | 5.70% | 61 | 19 | 6.00% | 71 | 22 | 6.30% | 73 |
| actin, cytoplasmic 2 | 316659409 | 42 | 18 | 60% | 163 | 18 | 65% | 138 | 19 | 60% | 130 |
| alpha-1-acid glycoprotein 1 precursor | 167857790 | 24 | 18 | 49% | 169 | 16 | 51% | 309 | 20 | 51% | 271 |
| carboxypeptidase E preproprotein | 4503009 | 53 | 17 | 46% | 146 | 19 | 48% | 145 | 19 | 45% | 176 |
| polymeric immunoglobulin receptor isoform X1 | 530366266 | 85 | 17 | 22% | 52 | 15 | 29% | 49 | 17 | 27% | 55 |
| matrilin-2 isoform a precursor | 62548860 | 107 | 17 | 24% | 39 | 12 | 19% | 37 | 18 | 26% | 63 |
| cation-independent mannose-6-phosphate receptor precursor | 119964726 | 274 | 16 | 7.10% | 39 | 27 | 11% | 60 | 26 | 12% | 66 |
| L-lactate dehydrogenase C chain | 9257228 | 36 | 16 | 47% | 98 | 16 | 49% | 81 | 17 | 62% | 95 |
| fructose-bisphosphatealdolase A isoform 2 | 342187211 | 45 | 16 | 64% | 64 | 16 | 61% | 75 | 15 | 56% | 76 |
| programmed cell death 6-interacting protein isoform 1 | 22027538 | 96 | 16 | 33% | 52 | 15 | 31% | 41 | 15 | 29% | 58 |
| cartilage acidic protein 1 isoform B precursor | 330688397 | 70 | 15 | 35% | 48 | 19 | 42% | 61 | 19 | 44% | 60 |
| alpha-enolase isoform 1 | 4503571 | 47 | 15 | 53% | 73 | 18 | 60% | 76 | 15 | 53% | 60 |
| lipoprotein lipase precursor | 4557727 | 53 | 14 | 41% | 64 | 14 | 45% | 71 | 18 | 44% | 76 |
| cysteine-rich secretory protein 1 isoform 1 precursor | 25121982 | 28 | 14 | 69% | 84 | 14 | 67% | 103 | 13 | 69% | 103 |
| glucose-6-phosphate isomerase isoform 3 | 577019448 | 67 | 14 | 36% | 52 | 13 | 39% | 43 | 16 | 44% | 46 |
| cystatin-S precursor | 4503109 | 16 | 13 | 74% | 133 | 20 | 74% | 135 | 15 | 74% | 152 |
| nucleobindin-2 isoform X1 | 578820554 | 50 | 13 | 37% | 56 | 16 | 52% | 56 | 14 | 45% | 59 |
| cathepsin D preproprotein | 4503143 | 45 | 13 | 40% | 67 | 16 | 47% | 96 | 13 | 46% | 75 |
| lysosomal alpha-glucosidase isoform X1 | 530411863 | 105 | 13 | 21% | 39 | 15 | 27% | 56 | 13 | 22% | 49 |
| heat shock 70 protein 1A/1B | 167466173 | 70 | 13 | 28% | 35 | 15 | 39% | 48 | 13 | 37% | 39 |
| aldose reductase | 4502049 | 36 | 13 | 62% | 39 | 14 | 65% | 45 | 13 | 65% | 53 |
| cathepsin B isoform X1 | 578815059 | 38 | 13 | 43% | 58 | 13 | 49% | 59 | 15 | 48% | 74 |
| alpha-2-antiplasmin isoform X1 | 530410436 | 62 | 13 | 27% | 42 | 13 | 37% | 60 | 11 | 25% | 41 |
| alpha-1-antichymotrypsin precursor | 50659080 | 48 | 13 | 35% | 82 | 12 | 39% | 75 | 16 | 49% | 87 |
| rab GDP dissociation inhibitor beta isoform 1 | 6598323 | 51 | 13 | 46% | 34 | 9 | 33% | 26 | 11 | 33% | 31 |
| triosephosphateisomerase isoform 1 | 4507645 | 27 | 12 | 72% | 33 | 14 | 73% | 38 | 11 | 70% | 47 |
| epididymal secretory protein E1 precursor | 5453678 | 17 | 12 | 52% | 119 | 13 | 63% | 130 | 15 | 70% | 140 |
| beta-mannosidase precursor | 84798622 | 101 | 12 | 16% | 28 | 13 | 24% | 26 | 13 | 19% | 31 |
| sorbitol dehydrogenase | 156627571 | 38 | 12 | 50% | 56 | 13 | 50% | 67 | 11 | 45% | 71 |
| peptidyl-prolylcis-trans isomerase B precursor | 4758950 | 24 | 12 | 51% | 38 | 12 | 55% | 39 | 12 | 58% | 46 |
| kallistatin isoform 2 precursor | 21361302 | 49 | 12 | 37% | 37 | 11 | 37% | 42 | 14 | 43% | 49 |
| transmembrane protease serine 2 isoform 2 | 205360943 | 54 | 12 | 38% | 47 | 11 | 36% | 30 | 13 | 48% | 54 |
| acid ceramidase isoform b | 189011546 | 47 | 12 | 33% | 43 | 10 | 31% | 45 | 9 | 32% | 43 |
| endoplasmin precursor | 4507677 | 92 | 12 | 20% | 75 | 9 | 11% | 51 | 10 | 17% | 77 |
| plasma protease C1 inhibitor precursor | 73858570 | 55 | 12 | 27% | 37 | 7 | 16% | 25 | 13 | 27% | 50 |
| alpha-N-acetylglucosaminidase precursor | 66346698 | 82 | 11 | 26% | 33 | 15 | 30% | 37 | 13 | 27% | 37 |
| attractin isoform 2 preproprotein | 21450863 | 141 | 11 | 12% | 25 | 14 | 12% | 30 | 14 | 12% | 39 |
| cytosolic non-specific dipeptidase isoform X2 | 530414265 | 53 | 11 | 23% | 32 | 14 | 47% | 34 | 9 | 27% | 28 |
| creatine kinase B-type | 21536286 | 43 | 11 | 51% | 61 | 13 | 54% | 73 | 14 | 54% | 67 |
| pyruvate kinase PKM isoform X1 | 530405975 | 65 | 11 | 28% | 52 | 13 | 33% | 46 | 11 | 31% | 41 |
| hemopexin precursor | 11321561 | 52 | 11 | 35% | 34 | 12 | 46% | 45 | 12 | 40% | 50 |
| annexin A5 | 4502107 | 36 | 11 | 41% | 27 | 11 | 43% | 31 | 12 | 46% | 32 |
| sialate O-acetylesterase isoform 1 precursor | 24850115 | 58 | 11 | 34% | 40 | 11 | 40% | 42 | 11 | 34% | 45 |
| ribonuclease T2 precursor | 5231228 | 29 | 11 | 49% | 55 | 11 | 46% | 71 | 10 | 46% | 69 |
| protein-glutamine gamma-glutamyltransferase 4 | 156627577 | 77 | 10 | 23% | 35 | 17 | 30% | 58 | 19 | 39% | 54 |
| fatty acid synthase | 41872631 | 273 | 10 | 3.30% | 26 | 15 | 5.10% | 29 | 18 | 6.40% | 45 |
| annexin A3 | 4826643 | 36 | 10 | 39% | 26 | 12 | 46% | 36 | 8 | 31% | 26 |
| 78 glucose-regulated protein precursor | 16507237 | 72 | 10 | 25% | 25 | 11 | 25% | 29 | 12 | 18% | 29 |
| glyceraldehyde-3-phosphate dehydrogenase isoform 1 | 576583524 | 36 | 10 | 41% | 66 | 10 | 47% | 65 | 12 | 45% | 55 |
| beta-hexosaminidase subunit alpha preproprotein | 189181666 | 61 | 10 | 29% | 28 | 10 | 29% | 34 | 12 | 34% | 32 |
| von Willebrand factor A domain-containing protein 1 isoform 1 precursor | 40068485 | 47 | 10 | 33% | 34 | 10 | 41% | 40 | 11 | 44% | 45 |
| cysteine-rich secretory protein 3 isoform 1 precursor | 300244560 | 29 | 10 | 54% | 66 | 9 | 50% | 61 | 10 | 53% | 69 |
| heat shock cognate 71 protein isoform X1 | 578822169 | 71 | 10 | 33% | 28 | 9 | 33% | 25 | 10 | 34% | 26 |
| gamma-glutamyltranspeptidase 1 precursor | 572152963 | 61 | 10 | 15% | 31 | 9 | 16% | 39 | 7 | 11% | 26 |
| peroxiredoxin-1 | 320461711 | 22 | 10 | 53% | 35 | 7 | 42% | 19 | 9 | 49% | 26 |
| prostaglandin-H2 D-isomerase precursor | 32171249 | 21 | 10 | 54% | 54 | 7 | 51% | 64 | 8 | 51% | 63 |
| beta-microseminoprotein isoform a precursor | 4557036 | 13 | 9 | 66% | 65 | 17 | 60% | 63 | 16 | 75% | 68 |
| alpha-2-macroglobulin isoform X1 | 578822814 | 167 | 9 | 5.20% | 18 | 13 | 6.40% | 21 | 13 | 10.00% | 24 |
| glutathione S-transferase Mu 3 | 23065552 | 27 | 9 | 45% | 32 | 12 | 54% | 38 | 15 | 64% | 51 |
| growth arrest-specific protein 6 isoform 1 precursor | 4557617 | 75 | 9 | 19% | 22 | 12 | 22% | 37 | 10 | 19% | 23 |
| annexin A1 | 4502101 | 39 | 9 | 42% | 21 | 11 | 45% | 31 | 9 | 36% | 25 |
| serpin B6 isoform a | 41152086 | 43 | 9 | 36% | 29 | 10 | 40% | 26 | 14 | 42% | 37 |
| ADP-ribosylcyclase 1 | 38454326 | 34 | 9 | 31% | 32 | 10 | 27% | 41 | 9 | 26% | 49 |
| amiloride-sensitive amine oxidase [copper-containing] isoform 1 precursor | 440918691 | 87 | 9 | 17% | 18 | 10 | 19% | 27 | 8 | 13% | 18 |
| tripeptidyl-peptidase 1 preproprotein | 5729770 | 61 | 9 | 31% | 24 | 9 | 33% | 29 | 9 | 32% | 30 |
| CD177 antigen precursor | 110735433 | 46 | 9 | 30% | 39 | 9 | 39% | 45 | 7 | 32% | 54 |
| metalloproteinase inhibitor 2 precursor | 4507511 | 24 | 9 | 50% | 24 | 8 | 42% | 19 | 13 | 58% | 38 |
| procollagen-lysine,2-oxoglutarate 5-dioxygenase 1 precursor | 32307144 | 84 | 9 | 19% | 18 | 8 | 16% | 21 | 11 | 24% | 24 |
| nucleotide exchange factor SIL1 isoform X1 | 578810496 | 53 | 9 | 25% | 31 | 8 | 24% | 33 | 10 | 28% | 40 |
| metalloproteinase inhibitor 1 precursor | 4507509 | 23 | 9 | 67% | 91 | 8 | 57% | 73 | 8 | 57% | 79 |
| legumainpreproprotein | 56682962 | 49 | 9 | 32% | 34 | 6 | 19% | 28 | 9 | 28% | 35 |
| phosphoglycerate kinase 1 | 4505763 | 45 | 9 | 39% | 22 | 6 | 36% | 22 | 9 | 43% | 30 |
| proteasome subunit alpha type-6 isoform a | 23110944 | 27 | 9 | 36% | 20 | 3 | 14% | 9 | 3 | 14% | 7 |
| A disintegrin and metalloproteinase with thrombospondin motifs 1 preproprotein | 50845384 | 105 | 8 | 13% | 20 | 11 | 17% | 24 | 11 | 16% | 33 |
| receptor-type tyrosine-protein phosphatase S isoform X1 | 530425335 | 215 | 8 | 7.30% | 27 | 11 | 7.30% | 33 | 8 | 5.70% | 22 |
| peroxiredoxin-6 | 4758638 | 25 | 8 | 48% | 26 | 10 | 56% | 27 | 8 | 53% | 25 |
| malate dehydrogenase, cytoplasmic isoform 1 | 312283701 | 39 | 8 | 32% | 24 | 10 | 44% | 32 | 8 | 33% | 25 |
| phospholipase A1 member A isoform 2 precursor | 332688256 | 48 | 8 | 23% | 44 | 10 | 38% | 74 | 6 | 19% | 46 |
| 72 type IV collagenase isoform b | 189217853 | 69 | 8 | 20% | 26 | 9 | 24% | 33 | 10 | 26% | 33 |
| L-lactate dehydrogenase B chain | 291575128 | 37 | 8 | 33% | 19 | 9 | 43% | 25 | 10 | 43% | 19 |
| phosphoglycerate kinase 2 | 31543397 | 45 | 8 | 31% | 25 | 9 | 36% | 28 | 9 | 31% | 25 |
| limbic system-associated membrane protein preproprotein | 45594240 | 37 | 8 | 29% | 35 | 9 | 32% | 41 | 8 | 28% | 33 |
| L-lactate dehydrogenase A chain isoform 1 | 5031857 | 37 | 8 | 38% | 23 | 9 | 41% | 26 | 6 | 38% | 17 |
| Golgi apparatus protein 1 isoform 2 precursor | 224586815 | 136 | 8 | 9.00% | 22 | 8 | 7.90% | 22 | 10 | 9.80% | 35 |
| 14-3-3 protein zeta/delta isoform X2 | 530389317 | 28 | 8 | 43% | 24 | 8 | 40% | 28 | 10 | 49% | 33 |
| elongation factor 1-alpha 1 | 4503471 | 50 | 8 | 24% | 42 | 8 | 26% | 50 | 9 | 32% | 58 |
| mesothelin isoform X1 | 530407442 | 68 | 8 | 10% | 18 | 8 | 10% | 18 | 9 | 13% | 23 |
| prostasinpreproprotein | 4506153 | 36 | 8 | 31% | 26 | 8 | 41% | 34 | 7 | 25% | 31 |
| ras-related protein Rab-3B | 19923750 | 25 | 8 | 49% | 25 | 7 | 47% | 23 | 8 | 49% | 22 |
| tubulin alpha-1A chain isoform 1 | 17986283 | 50 | 8 | 20% | 27 | 6 | 20% | 16 | 3 | 10% | 10 |
| protein OS-9 isoform X1 | 530399626 | 76 | 8 | 18% | 12 | 5 | 13% | 9 | 9 | 22% | 20 |
| antileukoproteinase precursor | 4507065 | 14 | 8 | 53% | 33 | 5 | 45% | 16 | 7 | 52% | 25 |
| protein DJ-1 isoform X1 | 530360487 | 20 | 7 | 64% | 25 | 10 | 64% | 28 | 11 | 74% | 40 |
| protein disulfide-isomerase A3 precursor | 21361657 | 57 | 7 | 20% | 13 | 10 | 28% | 24 | 8 | 17% | 25 |
| di-N-acetylchitobiase precursor | 4758092 | 44 | 7 | 29% | 21 | 10 | 43% | 27 | 8 | 37% | 27 |
| annexin A2 isoform 2 | 50845386 | 39 | 7 | 29% | 20 | 10 | 39% | 25 | 7 | 29% | 23 |
| cystatin-C precursor | 4503107 | 16 | 7 | 45% | 54 | 9 | 48% | 52 | 11 | 45% | 56 |
| vitamin D-binding protein isoform 3 precursor | 324021745 | 55 | 7 | 22% | 24 | 9 | 31% | 29 | 10 | 31% | 31 |
| carboxypeptidase M precursor | 38327526 | 51 | 7 | 12% | 28 | 8 | 9.50% | 23 | 9 | 12% | 29 |
| soluble calcium-activated nucleotidase 1 | 229577444 | 45 | 7 | 29% | 17 | 8 | 31% | 22 | 9 | 30% | 26 |
| kallikrein-2 isoform 1 preproprotein | 5031829 | 29 | 7 | 41% | 35 | 8 | 68% | 33 | 8 | 51% | 48 |
| epididymal sperm-binding protein 1 precursor | 301601648 | 26 | 7 | 34% | 24 | 7 | 29% | 20 | 8 | 32% | 23 |
| acrosin precursor | 148613878 | 46 | 7 | 21% | 23 | 7 | 19% | 29 | 8 | 25% | 28 |
| peroxiredoxin-2 | 32189392 | 22 | 7 | 32% | 17 | 7 | 34% | 21 | 8 | 34% | 25 |
| cysteine-rich secretory protein LCCL domain-containing 2 precursor | 13899332 | 56 | 7 | 18% | 18 | 7 | 18% | 19 | 8 | 22% | 21 |
| syntenin-1 isoform 1 | 55749490 | 32 | 7 | 40% | 26 | 7 | 44% | 24 | 7 | 37% | 31 |
| voltage-dependent calcium channel subunit alpha-2/delta-1 isoform X2 | 530386377 | 125 | 7 | 8.60% | 16 | 6 | 7.60% | 15 | 9 | 12% | 24 |
| acrosin-binding protein precursor | 17999524 | 61 | 7 | 27% | 19 | 6 | 17% | 14 | 8 | 22% | 26 |
| protein FAM3B isoform a precursor | 46255030 | 26 | 7 | 39% | 21 | 6 | 32% | 24 | 7 | 32% | 18 |
| peptidyl-prolylcis-trans isomerase A | 10863927 | 18 | 7 | 55% | 29 | 6 | 44% | 17 | 7 | 48% | 18 |
| alpha-1-acid glycoprotein 2 precursor | 4505529 | 24 | 7 | 51% | 33 | 6 | 48% | 52 | 6 | 48% | 48 |
| epididymal secretory protein E3-beta precursor | 11641279 | 18 | 7 | 46% | 35 | 6 | 44% | 31 | 6 | 44% | 42 |
| heat shock protein HSP 90-beta isoform X1 | 530381931 | 83 | 7 | 25% | 17 | 6 | 25% | 13 | 6 | 27% | 14 |
| monocyte differentiation antigen CD14 precursor | 291575163 | 40 | 7 | 35% | 16 | 5 | 20% | 14 | 7 | 35% | 16 |
| adenosylhomocysteinase isoform 1 | 9951915 | 48 | 7 | 20% | 15 | 4 | 12% | 11 | 8 | 19% | 22 |
| elongation factor 1-gamma | 4503481 | 50 | 7 | 19% | 23 | 4 | 14% | 13 | 5 | 17% | 14 |
| tissue alpha-L-fucosidase precursor | 119360348 | 54 | 6 | 27% | 14 | 10 | 39% | 38 | 9 | 39% | 22 |
| leucine-rich alpha-2-glycoprotein precursor | 16418467 | 38 | 6 | 29% | 21 | 10 | 43% | 33 | 8 | 34% | 29 |
| zonapellucida-binding protein 1 isoform 1 precursor | 229577313 | 40 | 6 | 27% | 40 | 9 | 19% | 29 | 8 | 21% | 31 |
| elongation factor 2 | 4503483 | 95 | 6 | 9.70% | 13 | 9 | 18% | 18 | 7 | 13% | 21 |
| phosphatidylethanolamine-binding protein 1 preproprotein | 4505621 | 21 | 6 | 52% | 21 | 9 | 69% | 20 | 6 | 55% | 20 |
| carboxypeptidase Z isoform 1 precursor | 62388877 | 74 | 6 | 14% | 19 | 9 | 24% | 27 | 4 | 10.00% | 15 |
| phosphatidylethanolamine-binding protein 4 precursor | 116812622 | 26 | 6 | 42% | 48 | 8 | 56% | 56 | 7 | 43% | 54 |
| glutathione reductase, mitochondrial isoform 2 precursor | 305410789 | 53 | 6 | 15% | 11 | 7 | 21% | 15 | 9 | 27% | 22 |
| pro-cathepsin H preproprotein | 23110955 | 37 | 6 | 24% | 19 | 7 | 21% | 24 | 8 | 24% | 29 |
| gastricsin isoform 1 preproprotein | 4505757 | 42 | 6 | 12% | 42 | 7 | 12% | 52 | 7 | 14% | 52 |
| collagen alpha-1(XVIII) chain isoform 2 precursor | 110611233 | 136 | 6 | 5.00% | 14 | 7 | 5.70% | 20 | 7 | 5.70% | 19 |
| CD109 antigen isoform 2 precursor | 227430301 | 160 | 6 | 8.00% | 12 | 7 | 9.00% | 21 | 7 | 9.30% | 25 |
| insulin-like growth factor-binding protein 2 precursor | 55925576 | 35 | 6 | 26% | 24 | 7 | 39% | 32 | 6 | 28% | 22 |
| prostate and testis expressed protein 1 precursor | 19923082 | 14 | 6 | 53% | 20 | 7 | 53% | 30 | 6 | 53% | 33 |
| ras-related protein Rab-27A | 19923264 | 25 | 6 | 36% | 14 | 7 | 37% | 23 | 6 | 30% | 20 |
| maltase-glucoamylase, intestinal isoform X1 | 578814724 | 312 | 6 | 3.10% | 10 | 6 | 2.90% | 15 | 10 | 4.70% | 20 |
| procollagen-lysine,2-oxoglutarate 5-dioxygenase 3 precursor | 4505891 | 85 | 6 | 13% | 12 | 6 | 14% | 16 | 7 | 15% | 14 |
| lactoylglutathionelyase | 118402586 | 21 | 6 | 42% | 12 | 6 | 38% | 16 | 7 | 53% | 21 |
| ribonuclease 4 precursor | 37577172 | 17 | 6 | 35% | 25 | 6 | 35% | 27 | 6 | 35% | 26 |
| cadherin-1 preproprotein | 4757960 | 97 | 6 | 8.40% | 13 | 6 | 8.40% | 14 | 5 | 8.40% | 16 |
| ceroid-lipofuscinosis neuronal protein 5 | 5729772 | 46 | 6 | 15% | 12 | 6 | 23% | 13 | 5 | 13% | 14 |
| ATP synthase subunit beta, mitochondrial precursor | 32189394 | 57 | 6 | 23% | 71 | 6 | 21% | 21 | 4 | 13% | 17 |
| kallikrein-11 isoform 1 precursor | 209862865 | 27 | 6 | 24% | 19 | 5 | 22% | 20 | 7 | 27% | 23 |
| alpha-actinin-4 isoform X2 | 530417304 | 104 | 6 | 8.80% | 12 | 5 | 8.40% | 15 | 7 | 11% | 17 |
| tubulin beta-4B chain | 5174735 | 50 | 6 | 18% | 15 | 5 | 19% | 15 | 6 | 20% | 23 |
| cullin-associated NEDD8-dissociated protein 1 | 21361794 | 136 | 6 | 8.80% | 15 | 5 | 7.20% | 12 | 6 | 8.70% | 17 |
| dipeptidase 3 isoform a precursor | 193211608 | 56 | 6 | 21% | 16 | 5 | 13% | 15 | 5 | 17% | 17 |
| glutathione S-transferase P | 4504183 | 23 | 6 | 50% | 20 | 4 | 38% | 12 | 7 | 56% | 18 |
| superoxide dismutase [Cu-Zn] | 4507149 | 16 | 6 | 60% | 23 | 4 | 60% | 13 | 5 | 64% | 19 |
| phosphoglyceratemutase 2 | 50593010 | 29 | 6 | 41% | 13 | 4 | 22% | 9 | 4 | 25% | 8 |
| protein disulfide-isomerase precursor | 20070125 | 57 | 6 | 14% | 15 | 4 | 11% | 13 | 4 | 16% | 15 |
| cathepsin Z preproprotein | 22538442 | 34 | 6 | 29% | 18 | 3 | 14% | 9 | 6 | 29% | 18 |
| cathelicidin antimicrobial peptide preproprotein | 348041314 | 20 | 5 | 31% | 27 | 8 | 43% | 30 | 9 | 43% | 41 |
| beta-galactosidase isoform a preproprotein | 119372308 | 76 | 5 | 11% | 12 | 8 | 17% | 23 | 7 | 13% | 20 |
| arylsulfatase A isoform a precursor | 313569795 | 54 | 5 | 20% | 15 | 8 | 24% | 22 | 6 | 22% | 13 |
| dipeptidyl peptidase 2 preproprotein | 62420888 | 54 | 5 | 17% | 11 | 7 | 25% | 20 | 10 | 34% | 22 |
| nucleobindin-1 precursor | 20070228 | 54 | 5 | 15% | 12 | 7 | 20% | 20 | 8 | 21% | 22 |
| neutral alpha-glucosidase AB isoform 2 precursor | 38202257 | 107 | 5 | 7.90% | 14 | 7 | 13% | 15 | 7 | 12% | 10 |
| multiple inositol polyphosphate phosphatase 1 isoform 1 precursor | 19923761 | 55 | 5 | 17% | 13 | 7 | 24% | 15 | 6 | 18% | 16 |
| complement factor B preproprotein | 67782358 | 86 | 5 | 9.40% | 12 | 7 | 14% | 17 | 6 | 11% | 13 |
| glycodelin precursor | 65507501 | 21 | 5 | 32% | 60 | 7 | 32% | 59 | 5 | 28% | 63 |
| aspartate aminotransferase, cytoplasmic | 4504067 | 46 | 5 | 21% | 14 | 7 | 26% | 15 | 5 | 24% | 15 |
| calsyntenin-1 isoform X1 | 530360505 | 108 | 5 | 5.70% | 13 | 6 | 12% | 16 | 9 | 14% | 18 |
| transthyretin precursor | 4507725 | 16 | 5 | 49% | 14 | 6 | 45% | 19 | 8 | 69% | 25 |
| extracellular superoxide dismutase [Cu-Zn] precursor | 118582275 | 26 | 5 | 40% | 18 | 6 | 44% | 34 | 6 | 44% | 29 |
| beta-2-microglobulin precursor | 4757826 | 14 | 5 | 43% | 32 | 5 | 38% | 30 | 6 | 45% | 34 |
| epididymal secretory protein E3-alpha precursor | 11386189 | 18 | 5 | 31% | 9 | 5 | 26% | 13 | 6 | 46% | 24 |
| carbonic anhydrase 2 | 4557395 | 29 | 5 | 23% | 13 | 5 | 25% | 10 | 6 | 30% | 18 |
| retinal dehydrogenase 1 | 21361176 | 55 | 5 | 17% | 9 | 5 | 19% | 8 | 4 | 15% | 10 |
| lysosome-associated membrane glycoprotein 2 isoform C precursor | 169790833 | 45 | 5 | 12% | 15 | 5 | 9.20% | 19 | 3 | 7.10% | 15 |
| trifunctional enzyme subunit alpha, mitochondrial precursor | 20127408 | 83 | 5 | 14% | 36 | 5 | 14% | 18 | 3 | 8.10% | 11 |
| WAP four-disulfide core domain protein 2 precursor | 56699495 | 13 | 5 | 45% | 28 | 4 | 45% | 20 | 5 | 45% | 31 |
| carboxypeptidase Q isoform X1 | 530388680 | 52 | 5 | 18% | 15 | 4 | 15% | 11 | 5 | 13% | 17 |
| phosphoglyceratemutase 1 | 4505753 | 29 | 5 | 48% | 11 | 4 | 41% | 11 | 5 | 48% | 13 |
| G-protein coupled receptor family C group 5 member C isoform a | 40217831 | 53 | 5 | 12% | 11 | 4 | 6.80% | 9 | 5 | 13% | 13 |
| beta-1,4-galactosyltransferase 1 | 13929462 | 44 | 5 | 23% | 15 | 4 | 19% | 19 | 4 | 19% | 11 |
| 14-3-3 protein theta | 5803227 | 28 | 5 | 38% | 11 | 3 | 23% | 9 | 3 | 23% | 9 |
| L-xylulosereductase isoform 2 | 304571975 | 26 | 5 | 33% | 13 | 2 | 11% | 5 | 5 | 36% | 13 |
| peroxiredoxin-4 precursor | 5453549 | 31 | 4 | 25% | 8 | 7 | 46% | 18 | 10 | 53% | 24 |
| kunitz-type protease inhibitor 1 isoform 2 precursor | 4504329 | 57 | 4 | 7.80% | 12 | 7 | 12% | 18 | 6 | 12% | 15 |
| biotinidase isoform 3 | 4557373 | 61 | 4 | 9.80% | 15 | 6 | 16% | 25 | 6 | 14% | 25 |
| alpha-galactosidase A precursor | 4504009 | 49 | 4 | 9.60% | 13 | 6 | 18% | 19 | 6 | 14% | 21 |
| complement C3 precursor | 115298678 | 187 | 4 | 3.30% | 9 | 6 | 5.80% | 19 | 4 | 3.10% | 13 |
| matrix-remodeling-associated protein 5 isoform X1 | 530421042 | 315 | 4 | 1.10% | 7 | 5 | 2.60% | 10 | 6 | 2.90% | 11 |
| beta-2-glycoprotein 1 precursor | 153266841 | 38 | 4 | 16% | 11 | 5 | 26% | 11 | 6 | 29% | 12 |
| T-complex protein 1 subunit alpha isoform a | 57863257 | 60 | 4 | 12% | 12 | 5 | 17% | 17 | 5 | 13% | 16 |
| complement C4-B-like preproprotein | 338858017 | 193 | 4 | 3.30% | 8 | 5 | 6.50% | 8 | 4 | 4.00% | 11 |
| acid sphingomyelinase-like phosphodiesterase 3a isoform X1 | 530383315 | 49 | 4 | 14% | 6 | 5 | 14% | 11 | 4 | 12% | 9 |
| transitional endoplasmic reticulum ATPase | 6005942 | 89 | 4 | 5.50% | 8 | 5 | 13% | 10 | 4 | 10% | 9 |
| protein disulfide-isomerase A6 isoform X3 | 530366876 | 49 | 4 | 13% | 9 | 5 | 13% | 8 | 4 | 8.90% | 9 |
| glutathione S-transferase theta-1 | 167466164 | 27 | 4 | 22% | 8 | 4 | 25% | 5 | 9 | 49% | 18 |
| ganglioside GM2 activator isoform 1 precursor | 39995109 | 21 | 4 | 23% | 12 | 4 | 31% | 14 | 6 | 41% | 18 |
| ribonuclease pancreatic precursor | 38201684 | 18 | 4 | 48% | 41 | 4 | 42% | 40 | 4 | 42% | 49 |
| cathepsin F precursor | 6042196 | 53 | 4 | 6.80% | 8 | 4 | 8.30% | 13 | 4 | 7.90% | 13 |
| growth/differentiation factor 15 precursor | 153792495 | 34 | 4 | 23% | 20 | 4 | 23% | 18 | 4 | 23% | 19 |
| selenoprotein S isoform 2 | 33285002 | 21 | 4 | 22% | 18 | 4 | 22% | 16 | 4 | 22% | 14 |
| regenerating islet-derived protein 3-gamma isoform 1 precursor | 38348213 | 19 | 4 | 34% | 10 | 4 | 34% | 13 | 4 | 34% | 17 |
| immunoglobulin lambda-like polypeptide 5 isoform 1 | 295986608 | 23 | 4 | 27% | 18 | 4 | 27% | 15 | 3 | 16% | 14 |
| bile salt-activated lipase precursor | 148536848 | 80 | 4 | 9.50% | 11 | 4 | 9.50% | 12 | 3 | 7.40% | 8 |
| selenoprotein P isoform 1 precursor | 148277018 | 43 | 4 | 8.40% | 10 | 4 | 14% | 17 | 3 | 12% | 15 |
| T-complex protein 1 subunit theta isoform 1 | 48762932 | 60 | 4 | 6.60% | 7 | 4 | 6.60% | 7 | 3 | 4.60% | 5 |
| heat shock 70 protein 1-like isoform X1 | 530381921 | 78 | 4 | 25% | 10 | 4 | 27% | 8 | 3 | 28% | 8 |
| cystatin-SA precursor | 4503105 | 16 | 4 | 53% | 11 | 4 | 58% | 13 | 3 | 52% | 11 |
| T-complex protein 1 subunit beta isoform 1 | 5453603 | 57 | 4 | 12% | 12 | 4 | 9.00% | 8 | 2 | 5.00% | 8 |
| ATP synthase subunit alpha, mitochondrial isoform c | 50345982 | 54 | 4 | 13% | 18 | 4 | 13% | 13 | 2 | 7.60% | 5 |
| 4-trimethylaminobutyraldehyde dehydrogenase | 115387104 | 56 | 4 | 9.70% | 6 | 4 | 8.10% | 9 | 2 | 4.20% | 3 |
| 14-3-3 protein epsilon isoform X1 | 530410617 | 27 | 4 | 35% | 14 | 3 | 18% | 8 | 7 | 40% | 20 |
| proteasome subunit beta type-1 | 4506193 | 26 | 4 | 24% | 10 | 3 | 15% | 6 | 6 | 32% | 17 |
| CD9 antigen | 4502693 | 25 | 4 | 21% | 24 | 3 | 21% | 27 | 5 | 15% | 28 |
| cystatin-SN precursor | 19882251 | 16 | 4 | 59% | 11 | 3 | 36% | 12 | 5 | 59% | 14 |
| retinoid-inducible serine carboxypeptidase precursor | 11055992 | 51 | 4 | 9.70% | 8 | 3 | 6.90% | 10 | 4 | 13% | 13 |
| serum amyloid P-component precursor | 4502133 | 25 | 4 | 18% | 9 | 3 | 16% | 3 | 4 | 19% | 10 |
| peptidase inhibitor 15 preproprotein | 7705676 | 29 | 4 | 26% | 9 | 3 | 22% | 9 | 4 | 29% | 11 |
| follistatin-related protein 1 precursor | 5901956 | 35 | 4 | 17% | 15 | 3 | 10% | 10 | 3 | 9.70% | 10 |
| acylamino-acid-releasing enzyme | 23510451 | 81 | 4 | 8.20% | 9 | 3 | 4.60% | 9 | 3 | 4.80% | 7 |
| sperm acrosome membrane-associated protein 1 precursor | 13569934 | 32 | 4 | 17% | 8 | 3 | 16% | 9 | 3 | 16% | 6 |
| NME1-NME2 protein | 66392203 | 30 | 4 | 17% | 8 | 2 | 11% | 7 | 4 | 18% | 9 |
| ras-related protein Rab-3D | 4759000 | 24 | 4 | 42% | 6 | 2 | 31% | 6 | 4 | 44% | 8 |
| N(4)-(beta-N-acetylglucosaminyl)-L-asparaginase isoform 1 preproprotein | 285002251 | 37 | 4 | 22% | 10 | 2 | 12% | 6 | 2 | 6.60% | 8 |
| glutamate carboxypeptidase 2 isoform 3 | 301500668 | 83 | 4 | 6.30% | 6 | 2 | 4.20% | 2 | 2 | 3.00% | 2 |
| L-lactate dehydrogenase A-like 6B | 15082234 | 42 | 4 | 15% | 18 | 2 | 10.00% | 6 | 1 | 9.70% | 12 |
| cAMP-dependent protein kinase type II-alpha regulatory subunit isoform X1 | 530372834 | 46 | 4 | 15% | 5 | 1 | 4.20% | 1 | 2 | 8.40% | 3 |
| guanylatecyclase soluble subunit beta-1 | 4504215 | 71 | 4 | 8.70% | 19 | 1 | 1.90% | 2 | 1 | 1.90% | 4 |
| fumaratehydratase, mitochondrial | 19743875 | 55 | 4 | 18% | 10 | 1 | 5.70% | 3 | 1 | 5.70% | 1 |
| ubiquitin-like modifier-activating enzyme 1 isoform X1 | 530421539 | 123 | 3 | 5.00% | 7 | 7 | 12% | 13 | 6 | 9.70% | 12 |
| contactin-associated protein-like 2 precursor | 7662350 | 148 | 3 | 3.70% | 9 | 6 | 8.10% | 14 | 5 | 6.40% | 13 |
| fructose-1,6-bisphosphatase 1 | 16579888 | 37 | 3 | 14% | 8 | 6 | 28% | 13 | 3 | 14% | 11 |
| leukemia inhibitory factor receptor precursor | 189083786 | 124 | 3 | 2.70% | 8 | 5 | 6.10% | 9 | 7 | 7.70% | 15 |
| proteasome subunit alpha type-2 | 4506181 | 26 | 3 | 24% | 8 | 5 | 38% | 14 | 6 | 44% | 16 |
| profilin-1 | 4826898 | 15 | 3 | 30% | 7 | 5 | 50% | 12 | 5 | 50% | 12 |
| ezrin | 21614499 | 69 | 3 | 4.90% | 9 | 5 | 13% | 14 | 4 | 7.70% | 11 |
| lysosome-associated membrane glycoprotein 1 precursor | 112380628 | 45 | 3 | 8.60% | 11 | 5 | 12% | 19 | 4 | 11% | 13 |
| endoplasmic reticulum aminopeptidase 1 isoform X1 | 530380009 | 108 | 3 | 4.10% | 8 | 5 | 7.00% | 13 | 4 | 4.90% | 8 |
| ubiquitin-40S ribosomal protein S27a precursor | 294459921 | 18 | 3 | 22% | 18 | 5 | 26% | 25 | 3 | 22% | 23 |
| lysosomal Pro-X carboxypeptidase isoform 1 preproprotein | 4826940 | 56 | 3 | 9.50% | 7 | 4 | 13% | 10 | 5 | 15% | 13 |
| amyloid beta A4 protein isoform h precursor | 324021738 | 85 | 3 | 5.10% | 10 | 4 | 7.80% | 18 | 5 | 9.30% | 20 |
| ras-related protein Rab-27B isoform X1 | 530414276 | 25 | 3 | 18% | 10 | 4 | 28% | 8 | 5 | 33% | 11 |
| olfactomedin-4 precursor | 32313593 | 57 | 3 | 5.10% | 4 | 4 | 11% | 10 | 4 | 6.90% | 10 |
| CD59 glycoprotein preproprotein | 10835165 | 14 | 3 | 25% | 13 | 4 | 25% | 18 | 4 | 25% | 22 |
| trypsin-1 preproprotein | 4506145 | 27 | 3 | 15% | 17 | 4 | 15% | 10 | 4 | 15% | 10 |
| metalloreductase STEAP4 isoform 1 | 100815815 | 52 | 3 | 12% | 9 | 4 | 15% | 12 | 4 | 14% | 12 |
| alpha-1B-glycoprotein precursor | 21071030 | 54 | 3 | 10% | 6 | 4 | 12% | 10 | 3 | 8.90% | 5 |
| apolipoprotein A-I preproprotein | 4557321 | 31 | 3 | 9.40% | 7 | 4 | 13% | 13 | 3 | 13% | 14 |
| sialidase-1 precursor | 4557791 | 45 | 3 | 12% | 8 | 4 | 12% | 11 | 3 | 12% | 9 |
| neuroserpin precursor | 170295807 | 46 | 3 | 13% | 11 | 4 | 16% | 14 | 3 | 13% | 14 |
| thioredoxin isoform 1 | 50592994 | 12 | 3 | 22% | 5 | 4 | 43% | 10 | 3 | 34% | 9 |
| collagen alpha-2(VI) chain isoform 2C2 precursor | 115527062 | 109 | 3 | 3.00% | 7 | 4 | 5.50% | 11 | 2 | 1.80% | 4 |
| N-acetylglucosamine-6-sulfatase precursor | 4504061 | 62 | 3 | 9.40% | 5 | 4 | 12% | 6 | 1 | 2.20% | 3 |
| glutaminyl-peptide cyclotransferase precursor | 6912618 | 41 | 3 | 23% | 8 | 4 | 22% | 10 | 1 | 3.90% | 3 |
| adenylyl cyclase-associated protein 1 isoform X1 | 530361588 | 52 | 3 | 9.90% | 10 | 3 | 9.90% | 10 | 4 | 12% | 20 |
| protein CutA isoform X1 | 578811722 | 19 | 3 | 33% | 11 | 3 | 33% | 8 | 4 | 41% | 10 |
| serine protease inhibitor Kazal-type 2 isoform 1 precursor | 413081531 | 14 | 3 | 51% | 28 | 3 | 51% | 22 | 3 | 51% | 29 |
| granulins isoform X1 | 530411988 | 64 | 3 | 4.00% | 7 | 3 | 7.60% | 5 | 3 | 4.40% | 5 |
| cystatin-M precursor | 4503113 | 17 | 3 | 35% | 8 | 3 | 35% | 9 | 3 | 35% | 9 |
| testis-expressed sequence 101 protein isoform 2 precursor | 194097358 | 27 | 3 | 12% | 7 | 3 | 16% | 11 | 3 | 16% | 15 |
| phospholipase A2, membrane associated precursor | 239915985 | 16 | 3 | 23% | 7 | 3 | 23% | 7 | 3 | 23% | 8 |
| proteasome subunit beta type-5 isoform 1 | 4506201 | 28 | 3 | 14% | 9 | 3 | 14% | 7 | 3 | 14% | 9 |
| NAD(P)H-hydrate epimerase precursor | 91984773 | 32 | 3 | 17% | 9 | 3 | 16% | 9 | 3 | 16% | 9 |
| prostate stem cell antigen preproprotein | 289547757 | 12 | 3 | 25% | 12 | 3 | 25% | 10 | 3 | 25% | 16 |
| Golgi-associated plant pathogenesis-related protein 1 isoform a | 11641247 | 17 | 3 | 28% | 7 | 3 | 28% | 7 | 2 | 17% | 6 |
| gamma-glutamylcyclotransferase isoform 1 | 13129018 | 21 | 3 | 18% | 8 | 3 | 18% | 8 | 2 | 13% | 3 |
| collectin-12 | 18641360 | 82 | 3 | 4.40% | 7 | 3 | 7.80% | 7 | 2 | 3.50% | 5 |
| cofilin-1 | 5031635 | 19 | 3 | 34% | 11 | 2 | 23% | 6 | 4 | 39% | 12 |
| G-protein coupled receptor 64 isoform 2 precursor | 119943116 | 110 | 3 | 3.50% | 8 | 2 | 2.70% | 8 | 4 | 6.00% | 15 |
| nephronectin isoform A precursor | 296011067 | 64 | 3 | 9.60% | 7 | 2 | 4.80% | 5 | 4 | 12% | 10 |
| CD63 antigen isoform A | 383872455 | 26 | 3 | 7.60% | 10 | 2 | 5.00% | 14 | 3 | 7.60% | 19 |
| plasma alpha-L-fucosidase precursor | 40068512 | 54 | 3 | 6.00% | 9 | 2 | 3.60% | 6 | 3 | 6.00% | 8 |
| sperm acrosome membrane-associated protein 3 | 27777653 | 23 | 3 | 19% | 5 | 2 | 12% | 6 | 3 | 18% | 8 |
| amyloid-like protein 2 isoform 2 precursor | 214010181 | 85 | 3 | 4.40% | 4 | 2 | 5.50% | 4 | 3 | 8.50% | 5 |
| D-dopachrome decarboxylase | 145386531 | 13 | 3 | 31% | 9 | 2 | 21% | 6 | 3 | 31% | 7 |
| Golgi membrane protein 1 | 29550838 | 45 | 3 | 7.70% | 5 | 2 | 6.00% | 4 | 2 | 6.00% | 5 |
| ferritin heavy chain | 56682959 | 21 | 3 | 18% | 5 | 2 | 9.80% | 4 | 2 | 9.80% | 4 |
| seizure 6-like protein 2 isoform 2 precursor | 166235136 | 98 | 3 | 5.10% | 7 | 2 | 2.30% | 2 | 1 | 0.88% | 3 |
| succinyl-CoA:3-ketoacid coenzyme A transferase 1, mitochondrial precursor | 4557817 | 56 | 3 | 10% | 9 | 2 | 10% | 3 | 1 | 4.00% | 2 |
| insulin-like growth factor-binding protein 4 precursor | 62243290 | 28 | 3 | 13% | 8 | 1 | 3.10% | 3 | 5 | 29% | 8 |
| ATP-citrate synthase isoform 2 | 38569423 | 120 | 3 | 2.70% | 4 | 1 | 1.50% | 5 | 5 | 6.20% | 8 |
| prostate and testis expressed protein 4 precursor | 221554530 | 11 | 3 | 30% | 8 | 1 | 12% | 3 | 3 | 26% | 8 |
| transforming protein RhoA precursor | 10835049 | 22 | 3 | 23% | 8 | 1 | 6.20% | 3 | 3 | 14% | 5 |
| calmodulin | 4502549 | 17 | 3 | 31% | 7 | 1 | 11% | 3 | 2 | 22% | 6 |
| desmoplakin isoform I | 58530840 | 332 | 3 | 1.00% | 4 | 1 | 0.31% | 1 | 1 | 0.35% | 1 |
| protein NOV homolog precursor | 4505423 | 39 | 3 | 14% | 8 | 1 | 4.20% | 3 | 1 | 4.20% | 3 |
| fibulin-2 isoform X1 | 578805783 | 127 | 3 | 2.40% | 3 | ni | ni | ni | 1 | 0.84% | 2 |
| importin subunit beta-1 isoform 2 | 449784879 | 81 | 3 | 4.70% | 3 | ni | ni | ni | 1 | 2.10% | 1 |
| 6-phosphofructokinase type C isoform 1 | 11321601 | 86 | 2 | 2.70% | 4 | 7 | 14% | 11 | 4 | 8.50% | 5 |
| metalloproteinase inhibitor 3 precursor | 4507513 | 24 | 2 | 9.50% | 6 | 5 | 25% | 12 | 6 | 28% | 15 |
| T-complex protein 1 subunit eta isoform a | 5453607 | 59 | 2 | 4.60% | 11 | 5 | 11% | 12 | 5 | 11% | 16 |
| N-acetyllactosaminide beta-1,3-N-acetylglucosaminyltransferase | 5802984 | 47 | 2 | 9.90% | 6 | 5 | 28% | 9 | 3 | 15% | 7 |
| sphingomyelinphosphodiesterase isoform 2 precursor | 300795589 | 70 | 2 | 4.00% | 6 | 5 | 12% | 13 | 1 | 1.70% | 3 |
| deoxyribonuclease-2-alpha precursor | 4503349 | 40 | 2 | 6.10% | 6 | 4 | 19% | 10 | 5 | 19% | 13 |
| chitinase domain-containing protein 1 isoform X2 | 530395670 | 48 | 2 | 5.70% | 3 | 4 | 9.00% | 11 | 4 | 13% | 8 |
| glyceraldehyde-3-phosphate dehydrogenase, testis-specific | 7657116 | 45 | 2 | 15% | 6 | 4 | 22% | 9 | 3 | 14% | 5 |
| selenium-binding protein 1 isoform 3 | 385137130 | 57 | 2 | 7.00% | 4 | 4 | 11% | 8 | 2 | 6.80% | 6 |
| vitamin K-dependent protein S preproprotein | 192447438 | 75 | 2 | 2.20% | 5 | 4 | 6.20% | 6 | 1 | 2.20% | 2 |
| inositol monophosphatase 1 isoform 2 | 221625487 | 37 | 2 | 6.80% | 4 | 3 | 13% | 10 | 4 | 17% | 13 |
| alcohol dehydrogenase [NADP(+)] | 320202986 | 37 | 2 | 5.50% | 9 | 3 | 17% | 8 | 4 | 19% | 9 |
| proteasome subunit beta type-3 | 22538465 | 23 | 2 | 17% | 4 | 3 | 19% | 4 | 4 | 27% | 8 |
| BPI fold-containing family B member 2 precursor | 15055535 | 49 | 2 | 8.30% | 8 | 3 | 13% | 5 | 3 | 13% | 5 |
| vitronectin precursor | 88853069 | 54 | 2 | 3.10% | 4 | 3 | 8.60% | 12 | 3 | 5.20% | 7 |
| proteasome subunit alpha type-7 | 4506189 | 28 | 2 | 15% | 6 | 3 | 16% | 9 | 3 | 19% | 10 |
| cell division control protein 42 homolog isoform 1 precursor | 4757952 | 21 | 2 | 20% | 8 | 3 | 20% | 6 | 3 | 20% | 7 |
| acetyl-CoA acetyltransferase, cytosolic | 148539872 | 41 | 2 | 6.80% | 4 | 3 | 19% | 8 | 3 | 21% | 6 |
| ras-related protein Rab-7a | 34147513 | 23 | 2 | 13% | 3 | 3 | 18% | 4 | 3 | 18% | 4 |
| glypican-1 precursor | 167001141 | 62 | 2 | 4.80% | 4 | 3 | 7.30% | 7 | 3 | 7.70% | 5 |
| N-acetylglucosamine-1-phosphotransferase subunit gamma precursor | 14249738 | 34 | 2 | 20% | 4 | 3 | 23% | 5 | 2 | 20% | 5 |
| beta-glucuronidase isoform 1 precursor | 268834192 | 75 | 2 | 3.10% | 4 | 3 | 10% | 3 | 2 | 4.10% | 4 |
| protein FAM3C precursor | 7661714 | 25 | 2 | 15% | 6 | 3 | 20% | 7 | 2 | 13% | 5 |
| saccharopine dehydrogenase-like oxidoreductase | 55770836 | 47 | 2 | 12% | 15 | 3 | 15% | 12 | 2 | 12% | 14 |
| protein S100-A11 | 5032057 | 12 | 2 | 24% | 6 | 3 | 34% | 8 | 2 | 24% | 6 |
| puromycin-sensitive aminopeptidase | 158937236 | 103 | 2 | 2.40% | 3 | 3 | 6.00% | 4 | 2 | 5.00% | 4 |
| cathepsin L1 isoform 1 preproprotein | 22202619 | 38 | 2 | 7.80% | 4 | 3 | 7.80% | 5 | 1 | 3.60% | 4 |
| kunitz-type protease inhibitor 3 precursor | 189571689 | 10 | 2 | 19% | 12 | 3 | 29% | 13 | 1 | 19% | 8 |
| sialic acid synthase | 12056473 | 40 | 2 | 7.80% | 6 | 3 | 8.90% | 4 | 1 | 7.80% | 2 |
| acid sphingomyelinase-like phosphodiesterase 3b isoform 1 precursor | 57242798 | 51 | 2 | 6.20% | 4 | 3 | 11% | 5 | 1 | 2.60% | 3 |
| cysteine-rich secretory protein LCCL domain-containing 1 isoform 1 precursor | 13899303 | 57 | 2 | 6.00% | 6 | 2 | 5.40% | 6 | 4 | 11% | 8 |
| cAMP-dependent protein kinase catalytic subunit alpha isoform 1 | 4506055 | 41 | 2 | 6.80% | 7 | 2 | 10% | 8 | 4 | 8.50% | 10 |
| ras-related C3 botulinum toxin substrate 1 isoform Rac1b | 9845509 | 23 | 2 | 9.00% | 3 | 2 | 7.10% | 4 | 4 | 21% | 7 |
| calpain-1 catalytic subunit isoform X1 | 578821763 | 82 | 2 | 3.80% | 4 | 2 | 6.20% | 4 | 4 | 9.20% | 7 |
| 2,4-dienoyl-CoA reductase, mitochondrial precursor | 4503301 | 36 | 2 | 13% | 24 | 2 | 13% | 14 | 3 | 22% | 33 |
| tetraspanin-1 | 21264578 | 26 | 2 | 7.50% | 11 | 2 | 7.50% | 15 | 3 | 11% | 15 |
| 6-phosphogluconate dehydrogenase, decarboxylating | 40068518 | 53 | 2 | 5.20% | 3 | 2 | 6.60% | 6 | 3 | 9.50% | 5 |
| CD44 antigen isoform 1 precursor | 48255935 | 82 | 2 | 3.00% | 6 | 2 | 1.60% | 4 | 3 | 4.20% | 7 |
| zymogen granule protein 16 homolog B precursor | 94536866 | 23 | 2 | 14% | 4 | 2 | 14% | 4 | 3 | 18% | 9 |
| glutathione peroxidase 3 precursor | 6006001 | 26 | 2 | 14% | 6 | 2 | 14% | 6 | 3 | 19% | 7 |
| malate dehydrogenase, mitochondrial isoform 1 precursor | 21735621 | 36 | 2 | 8.90% | 2 | 2 | 5.60% | 5 | 3 | 9.50% | 6 |
| dihydrolipoyl dehydrogenase, mitochondrial isoform 3 | 576583539 | 52 | 2 | 11% | 62 | 2 | 11% | 29 | 2 | 11% | 43 |
| fibronectin isoform 3 preproprotein | 16933542 | 259 | 2 | 52% | 16 | 2 | 60% | 26 | 2 | 52% | 26 |
| protein lifeguard 3 | 50593008 | 35 | 2 | 4.80% | 10 | 2 | 4.80% | 10 | 2 | 4.80% | 12 |
| choline transporter-like protein 4 isoform 1 | 148612887 | 79 | 2 | 3.10% | 9 | 2 | 3.10% | 12 | 2 | 3.10% | 10 |
| 60 heat shock protein, mitochondrial isoform X1 | 530370277 | 61 | 2 | 9.10% | 14 | 2 | 9.10% | 9 | 2 | 9.10% | 10 |
| NADH-ubiquinone oxidoreductase 75 subunit, mitochondrial isoform 2 precursor | 316983154 | 75 | 2 | 6.50% | 17 | 2 | 6.50% | 14 | 2 | 6.50% | 10 |
| proteasome subunit alpha type-3 isoform 2 | 23110939 | 28 | 2 | 8.90% | 6 | 2 | 13% | 4 | 2 | 8.90% | 4 |
| ras-related protein Rap-1b isoform 3 | 354459354 | 19 | 2 | 14% | 6 | 2 | 14% | 6 | 2 | 14% | 6 |
| phosphoglucomutase-1 isoform 1 | 21361621 | 61 | 2 | 9.80% | 6 | 2 | 9.80% | 7 | 2 | 9.80% | 7 |
| chloride intracellular channel protein 1 | 14251209 | 27 | 2 | 19% | 6 | 2 | 12% | 5 | 2 | 12% | 8 |
| bifunctional epoxide hydrolase 2 isoform b | 374532804 | 57 | 2 | 4.20% | 4 | 2 | 11% | 6 | 2 | 4.20% | 6 |
| disintegrin and metalloproteinase domain-containing protein 10 precursor | 4557251 | 84 | 2 | 3.90% | 5 | 2 | 3.90% | 6 | 2 | 3.90% | 5 |
| proteasome subunit beta type-6 isoform 1 proprotein | 23110925 | 25 | 2 | 8.40% | 2 | 2 | 21% | 3 | 2 | 8.80% | 4 |
| dihydropteridinereductase | 208973246 | 26 | 2 | 11% | 3 | 2 | 11% | 3 | 2 | 11% | 6 |
| trifunctional enzyme subunit beta, mitochondrial isoform 1 precursor | 4504327 | 51 | 2 | 8.90% | 24 | 2 | 8.90% | 15 | 1 | 5.50% | 10 |
| peptidyl-prolylcis-trans isomerase C precursor | 4505991 | 23 | 2 | 9.90% | 6 | 2 | 9.90% | 4 | 1 | 6.10% | 3 |
| hypoxanthine-guanine phosphoribosyltransferase | 4504483 | 25 | 2 | 11% | 6 | 2 | 9.60% | 4 | 1 | 4.60% | 2 |
| hornerin | 57864582 | 282 | 2 | 1.70% | 6 | 2 | 1.50% | 3 | 1 | 0.84% | 2 |
| cAMP-dependent protein kinase type I-alpha regulatory subunit isoform a | 47132581 | 43 | 2 | 11% | 13 | 2 | 11% | 7 | 1 | 6.30% | 1 |
| dnaJ homolog subfamily C member 3 precursor | 5453980 | 58 | 2 | 4.20% | 4 | 1 | 3.80% | 1 | 4 | 7.70% | 10 |
| latent-transforming growth factor beta-binding protein 3 isoform 1 precursor | 194328809 | 139 | 2 | 3.40% | 4 | 1 | 1.30% | 3 | 4 | 5.00% | 6 |
| isoaspartyl peptidase/L-asparaginase | 145275200 | 32 | 2 | 16% | 3 | 1 | 10% | 3 | 4 | 21% | 8 |
| neuropilin-1 isoform a precursor | 182508169 | 103 | 2 | 4.60% | 5 | 1 | 1.50% | 3 | 3 | 6.10% | 5 |
| heat shock protein beta-1 | 4504517 | 23 | 2 | 13% | 6 | 1 | 8.30% | 3 | 3 | 21% | 7 |
| proteasome subunit beta type-4 | 22538467 | 29 | 2 | 14% | 2 | 1 | 5.70% | 3 | 3 | 18% | 5 |
| calreticulin precursor | 4757900 | 48 | 2 | 16% | 7 | 1 | 7.00% | 6 | 2 | 11% | 6 |
| insulin-like growth factor-binding protein 5 precursor | 10834982 | 31 | 2 | 11% | 12 | 1 | 5.90% | 3 | 2 | 11% | 5 |
| ribose-phosphate pyrophosphokinase 2 isoform 2 | 4506129 | 35 | 2 | 10% | 6 | 1 | 4.70% | 3 | 2 | 10% | 8 |
| serine/threonine-protein phosphatase 2A activator isoform b | 30065643 | 37 | 2 | 13% | 7 | 1 | 9.00% | 2 | 2 | 13% | 5 |
| interferon-induced transmembrane protein 1 | 150010589 | 14 | 2 | 27% | 5 | 1 | 13% | 3 | 2 | 27% | 3 |
| proteasome subunit alpha type-1 isoform 1 | 23110935 | 30 | 2 | 10% | 6 | 1 | 5.60% | 1 | 2 | 10% | 4 |
| nucleoside diphosphate kinase 3 precursor | 37693993 | 19 | 2 | 20% | 4 | 1 | 10% | 3 | 2 | 16% | 5 |
| thrombospondin-4 precursor | 31543806 | 106 | 2 | 2.60% | 4 | 1 | 2.60% | 2 | 2 | 4.30% | 4 |
| rho GDP-dissociation inhibitor 1 isoform a | 4757768 | 23 | 2 | 16% | 4 | 1 | 7.40% | 3 | 2 | 16% | 5 |
| antithrombin-III isoform X1 | 530364787 | 47 | 2 | 6.70% | 2 | 1 | 6.00% | 1 | 1 | 6.70% | 1 |
| twisted gastrulation protein homolog 1 precursor | 10190664 | 25 | 2 | 11% | 7 | 1 | 6.70% | 7 | 1 | 6.70% | 9 |
| L-seryl-tRNA(Sec) kinase isoform X1 | 530392997 | 41 | 2 | 3.60% | 2 | 1 | 3.40% | 6 | 1 | 3.40% | 1 |
| ruvB-like 1 | 4506753 | 50 | 2 | 6.60% | 3 | 1 | 2.60% | 1 | 1 | 3.10% | 3 |
| protein CREG1 precursor | 4503037 | 24 | 2 | 12% | 5 | 1 | 9.50% | 3 | 1 | 9.50% | 8 |
| hydroxyacyl-coenzyme A dehydrogenase, mitochondrial isoform 1 precursor | 296179427 | 36 | 2 | 19% | 5 | 1 | 7.90% | 4 | 1 | 7.90% | 4 |
| mammalian ependymin-related protein 1 isoform 1 precursor | 345110632 | 25 | 2 | 8.90% | 2 | 1 | 4.90% | 3 | 1 | 4.00% | 3 |
| xaa-Pro dipeptidase isoform 1 | 149589008 | 55 | 2 | 7.70% | 4 | 1 | 5.70% | 3 | 1 | 5.70% | 3 |
| CD81 antigen | 4757944 | 26 | 2 | 15% | 9 | ni | ni | ni | 2 | 18% | 2 |
| cytochrome b-c1 complex subunit 2, mitochondrial precursor | 50592988 | 48 | 2 | 13% | 5 | ni | ni | ni | 2 | 8.20% | 3 |
| ral guanine nucleotide dissociation stimulator-like 2 isoform 2 | 344189933 | 75 | 2 | 9.50% | 2 | ni | ni | ni | ni | ni | ni |
| transmembrane emp24 domain-containing protein 4 precursor | 33457308 | 26 | 2 | 8.80% | 2 | ni | ni | ni | ni | ni | ni |
| leucine-rich repeat-containing protein 37B precursor | 53829385 | 106 | 2 | 5.40% | 6 | ni | ni | ni | ni | ni | ni |
| beta-galactoside alpha-2,6-sialyltransferase 1 isoform X5 | 578807681 | 47 | 1 | 1.70% | 1 | 5 | 16% | 11 | 3 | 10% | 6 |
| protein dopey-2 isoform X1 | 578836735 | 258 | 1 | 0.44% | 1 | 4 | 1.30% | 5 | 4 | 2.00% | 11 |
| myosin-9 | 12667788 | 227 | 1 | 0.36% | 2 | 4 | 2.70% | 6 | 3 | 1.60% | 4 |
| EGF-containing fibulin-like extracellular matrix protein 1 precursor | 86788132 | 55 | 1 | 4.70% | 7 | 4 | 13% | 14 | 3 | 9.70% | 15 |
| alpha-N-acetylgalactosaminidase isoform X1 | 530420112 | 47 | 1 | 2.90% | 3 | 4 | 14% | 7 | 2 | 5.60% | 4 |
| uncharacterized protein LOC100293211 | 578798051 | 29 | 1 | 4.10% | 3 | 4 | 20% | 9 | 2 | 8.10% | 4 |
| N(G),N(G)-dimethylargininedimethylaminohydrolase 1 isoform 1 | 6912328 | 31 | 1 | 9.10% | 2 | 3 | 27% | 6 | 4 | 34% | 7 |
| vacuolar protein sorting-associated protein 28 homolog isoform X6 | 578816018 | 23 | 1 | 7.40% | 2 | 3 | 25% | 4 | 4 | 30% | 6 |
| endothelial lipase isoform X1 | 530414043 | 61 | 1 | 4.90% | 1 | 3 | 8.40% | 4 | 3 | 13% | 7 |
| sperm acrosome-associated protein 5 precursor | 120952755 | 18 | 1 | 8.20% | 1 | 3 | 23% | 7 | 3 | 21% | 7 |
| angiogenin precursor | 148277046 | 17 | 1 | 11% | 3 | 3 | 31% | 10 | 2 | 24% | 7 |
| lysosomal acid lipase/cholesteryl ester hydrolase isoform 1 precursor | 189083851 | 45 | 1 | 7.50% | 4 | 3 | 14% | 8 | 2 | 12% | 6 |
| protein MENT isoform X1 | 578801150 | 37 | 1 | 6.20% | 4 | 3 | 11% | 6 | 2 | 5.60% | 7 |
| ester hydrolase C11orf54 isoform c | 554506535 | 33 | 1 | 2.00% | 1 | 3 | 16% | 7 | 2 | 9.80% | 4 |
| annexin A11 isoform X1 | 530393508 | 66 | 1 | 2.00% | 2 | 3 | 4.50% | 5 | 2 | 1.50% | 2 |
| proteasome subunit alpha type-4 isoform 1 | 156713442 | 29 | 1 | 8.40% | 2 | 3 | 15% | 6 | 2 | 11% | 5 |
| homogentisate 1,2-dioxygenase isoform X2 | 530374530 | 38 | 1 | 3.60% | 3 | 3 | 16% | 7 | 2 | 6.50% | 4 |
| gamma-glutamyl hydrolase precursor | 4503987 | 36 | 1 | 7.50% | 3 | 3 | 17% | 5 | 1 | 7.50% | 3 |
| interleukin-1 receptor-like 1 isoform X1 | 578805137 | 63 | 1 | 1.40% | 2 | 2 | 3.10% | 5 | 6 | 12% | 11 |
| protein phosphatase 1 regulatory subunit 7 isoform 1 | 4506013 | 42 | 1 | 5.00% | 3 | 2 | 8.10% | 5 | 3 | 13% | 6 |
| acrosomal protein SP-10 isoform a precursor | 4501879 | 28 | 1 | 6.00% | 2 | 2 | 15% | 5 | 3 | 18% | 5 |
| ras-related protein Rab-2A isoform a | 4506365 | 24 | 1 | 6.10% | 1 | 2 | 12% | 5 | 3 | 19% | 7 |
| glutathione S-transferase Mu 1 isoform 1 | 23065544 | 26 | 1 | 6.00% | 3 | 2 | 14% | 6 | 3 | 20% | 8 |
| titin isoform IC | 388998877 | 3994 | 1 | 0.12% | 1 | 2 | 0.12% | 2 | 2 | 0.04% | 2 |
| glutathione synthetase isoform X2 | 530418095 | 52 | 1 | 4.20% | 4 | 2 | 4.20% | 11 | 2 | 6.10% | 13 |
| interleukin-6 receptor subunit beta isoform 1 precursor | 28610147 | 104 | 1 | 1.90% | 3 | 2 | 4.10% | 5 | 2 | 2.70% | 4 |
| prostate and testis expressed protein 2 precursor | 47086459 | 13 | 1 | 12% | 3 | 2 | 27% | 4 | 2 | 27% | 6 |
| solute carrier family 2, facilitated glucose transporter member 5 isoform 1 | 4507013 | 55 | 1 | 4.20% | 1 | 2 | 4.20% | 3 | 2 | 6.40% | 2 |
| junctional adhesion molecule A precursor | 8393638 | 33 | 1 | 3.70% | 3 | 2 | 7.70% | 5 | 2 | 7.70% | 5 |
| ADP-ribosylation factor 1 | 4502201 | 21 | 1 | 8.30% | 1 | 2 | 15% | 4 | 2 | 15% | 3 |
| EGF-containing fibulin-like extracellular matrix protein 2 precursor | 320118911 | 49 | 1 | 3.40% | 2 | 2 | 6.10% | 3 | 2 | 6.10% | 4 |
| protein Niban | 16757970 | 103 | 1 | 1.30% | 3 | 2 | 2.40% | 4 | 2 | 2.80% | 6 |
| alpha-actinin-1 isoform a | 194097350 | 106 | 1 | 9.30% | 2 | 2 | 8.30% | 2 | 2 | 13% | 3 |
| angiotensinogen preproprotein | 4557287 | 53 | 1 | 4.90% | 3 | 2 | 7.40% | 4 | 1 | 4.90% | 3 |
| galactoside 3(4)-L-fucosyltransferase | 148277014 | 42 | 1 | 4.40% | 3 | 2 | 7.20% | 6 | 1 | 4.40% | 2 |
| semaphorin-3F isoform X2 | 530372974 | 88 | 1 | 3.80% | 2 | 2 | 5.50% | 4 | 1 | 3.80% | 2 |
| acetyl-CoA acetyltransferase, mitochondrial isoform X1 | 578822176 | 35 | 1 | 6.50% | 15 | 2 | 8.00% | 10 | 1 | 6.50% | 8 |
| carbonic anhydrase 4 precursor | 4502519 | 35 | 1 | 4.80% | 3 | 2 | 9.00% | 4 | 1 | 4.80% | 3 |
| proteasome subunit alpha type-5 isoform 1 | 23110942 | 26 | 1 | 2.90% | 2 | 2 | 7.90% | 2 | 1 | 2.90% | 3 |
| hsc70-interacting protein isoform 1 | 19923193 | 41 | 1 | 3.80% | 3 | 2 | 5.70% | 4 | 1 | 3.80% | 3 |
| probable serine carboxypeptidase CPVL isoform X1 | 530384848 | 54 | 1 | 2.90% | 2 | 2 | 5.70% | 4 | 1 | 2.90% | 2 |
| lipid phosphate phosphohydrolase 1 isoform 2 | 29171738 | 32 | 1 | 7.00% | 4 | 2 | 10% | 11 | 1 | 7.00% | 2 |
| platelet-activating factor acetylhydrolase isoform X1 | 530382617 | 50 | 1 | 4.80% | 6 | 2 | 9.50% | 4 | 1 | 4.80% | 6 |
| transforming growth factor beta receptor type 3 isoform b precursor | 307574634 | 93 | 1 | 1.80% | 1 | 2 | 3.30% | 3 | 1 | 1.80% | 1 |
| lumican precursor | 4505047 | 38 | 1 | 5.30% | 3 | 2 | 7.70% | 4 | 1 | 5.30% | 3 |
| complement C2 isoform 1 preproprotein | 14550407 | 83 | 1 | 1.30% | 1 | 2 | 2.50% | 2 | 1 | 1.30% | 2 |
| immunoglobulin J chain precursor | 21489959 | 18 | 1 | 7.50% | 1 | 2 | 13% | 3 | ni | ni | ni |
| histone H4 | 11415030 | 11 | 1 | 7.80% | 3 | 2 | 17% | 7 | ni | ni | ni |
| probable inactive ribonuclease-like protein 13 precursor | 59276062 | 18 | 1 | 10% | 3 | 1 | 6.40% | 3 | 5 | 26% | 9 |
| mucin-5B precursor | 301172750 | 596 | 1 | 0.19% | 3 | 1 | 0.17% | 1 | 3 | 0.59% | 5 |
| cytochrome c | 11128019 | 12 | 1 | 7.60% | 3 | 1 | 7.60% | 3 | 3 | 25% | 7 |
| glyoxalase domain-containing protein 4 isoform X1 | 578829940 | 32 | 1 | 4.50% | 3 | 1 | 3.50% | 1 | 3 | 12% | 8 |
| ceruloplasmin precursor | 4557485 | 122 | 1 | 1.20% | 3 | 1 | 1.20% | 3 | 2 | 2.80% | 4 |
| neutrophil gelatinase-associated lipocalin precursor | 38455402 | 23 | 1 | 7.60% | 1 | 1 | 7.60% | 2 | 2 | 18% | 4 |
| hypoxia up-regulated protein 1 precursor | 5453832 | 111 | 1 | 1.50% | 1 | 1 | 1.50% | 1 | 2 | 2.30% | 5 |
| N-sulphoglucosaminesulphohydrolase isoform X1 | 530412678 | 39 | 1 | 7.50% | 3 | 1 | 7.50% | 3 | 2 | 11% | 6 |
| putative phospholipase B-like 2 isoform 1 precursor | 229093316 | 65 | 1 | 2.00% | 3 | 1 | 2.20% | 1 | 2 | 4.40% | 2 |
| thrombospondin-1 precursor | 40317626 | 129 | 1 | 2.00% | 3 | 1 | 2.00% | 1 | 2 | 3.00% | 5 |
| proteasome subunit beta type-2 isoform 2 | 315139006 | 20 | 1 | 6.20% | 3 | 1 | 6.20% | 3 | 2 | 15% | 6 |
| vasorin precursor | 88702793 | 72 | 1 | 4.20% | 3 | 1 | 4.20% | 3 | 2 | 6.50% | 4 |
| desmocollin-2 isoform Dsc2b preproprotein | 13435366 | 94 | 1 | 1.70% | 3 | 1 | 1.70% | 3 | 2 | 2.80% | 3 |
| platelet-derived growth factor subunit A isoform X2 | 578813519 | 24 | 1 | 3.80% | 3 | 1 | 3.80% | 3 | 2 | 9.00% | 4 |
| trefoil factor 3 precursor | 281485608 | 10 | 1 | 19% | 2 | 1 | 19% | 3 | 2 | 19% | 5 |
| tubulin alpha-1B chain | 57013276 | 50 | 1 | 17% | 1 | 1 | 19% | 3 | 2 | 10% | 2 |
| calcium and integrin-binding protein 1 isoform a | 480540325 | 26 | 1 | 6.50% | 3 | 1 | 6.50% | 1 | 2 | 13% | 4 |
| macrophage migration inhibitory factor | 4505185 | 12 | 1 | 9.60% | 4 | 1 | 9.60% | 5 | 1 | 9.60% | 9 |
| 45 calcium-binding protein isoform 2 precursor | 18699732 | 42 | 1 | 3.30% | 1 | 1 | 7.70% | 2 | 1 | 3.30% | 2 |
| HD domain-containing protein 2 | 116875826 | 23 | 1 | 8.30% | 3 | 1 | 8.30% | 3 | 1 | 8.30% | 3 |
| protein disulfide-isomerase A4 precursor | 4758304 | 73 | 1 | 3.10% | 3 | 1 | 1.90% | 3 | 1 | 1.90% | 3 |
| fructose-bisphosphatealdolase C isoform X1 | 530410965 | 48 | 1 | 13% | 3 | 1 | 13% | 3 | 1 | 5.10% | 4 |
| 4F2 cell-surface antigen heavy chain isoform c | 65506891 | 68 | 1 | 1.90% | 3 | 1 | 1.90% | 2 | 1 | 1.90% | 3 |
| dystroglycanpreproprotein | 294997302 | 98 | 1 | 2.30% | 3 | 1 | 3.70% | 1 | 1 | 2.30% | 2 |
| spermine synthase isoform 2 | 386643030 | 35 | 1 | 8.90% | 2 | 1 | 8.90% | 3 | 1 | 13% | 1 |
| delta and Notch-like epidermal growth factor-related receptor precursor | 116235485 | 78 | 1 | 2.00% | 2 | 1 | 2.00% | 2 | 1 | 2.00% | 2 |
| dermcidin isoform X1 | 530399720 | 12 | 1 | 9.10% | 1 | 1 | 9.10% | 2 | 1 | 9.10% | 2 |
| heat shock 70 protein 13 precursor | 48928056 | 52 | 1 | 2.10% | 3 | 1 | 2.50% | 3 | ni | ni | ni |
| translin isoform 1 | 4759270 | 26 | 1 | 6.60% | 1 | ni | ni | ni | 3 | 22% | 5 |
| anterior gradient protein 2 homolog isoform X1 | 530384410 | 20 | 1 | 10% | 4 | ni | ni | ni | 2 | 16% | 5 |
| dynein light chain 2, cytoplasmic | 18087855 | 10 | 1 | 7.90% | 2 | ni | ni | ni | 2 | 20% | 4 |
| galectin-3 isoform 1 | 115430223 | 26 | 1 | 5.20% | 2 | ni | ni | ni | 2 | 13% | 4 |
| procollagen-lysine,2-oxoglutarate 5-dioxygenase 2 isoform X1 | 530374784 | 77 | 1 | 2.10% | 3 | ni | ni | ni | 2 | 4.50% | 4 |
| MAM domain-containing protein 2 precursor | 223278413 | 78 | 1 | 1.60% | 2 | ni | ni | ni | 1 | 1.60% | 2 |
| N-acetylated-alpha-linked acidic dipeptidase 2 | 4885505 | 84 | 1 | 1.50% | 2 | ni | ni | ni | 1 | 1.50% | 1 |
| bifunctional ATP-dependent dihydroxyacetone kinase/FAD-AMP lyase (cyclizing) isoform X3 | 578821282 | 59 | 1 | 2.10% | 1 | ni | ni | ni | 1 | 4.30% | 1 |
| torsin-1B precursor | 14149653 | 38 | 1 | 4.50% | 1 | ni | ni | ni | 1 | 4.50% | 3 |
| alpha-2-HS-glycoprotein preproprotein | 156523970 | 39 | 1 | 1.90% | 1 | ni | ni | ni | ni | ni | ni |
| lipolysis-stimulated lipoprotein receptor isoform 4 | 386781455 | 69 | 1 | 2.20% | 3 | ni | ni | ni | ni | ni | ni |
| gelsolin isoform X8 | 578817385 | 82 | 1 | 2.00% | 1 | ni | ni | ni | ni | ni | ni |
| vesicle-associated membrane protein 2 | 172072620 | 13 | 1 | 21% | 1 | ni | ni | ni | ni | ni | ni |
| ubiquitin-conjugating enzyme E2 L3 isoform 3 | 373432682 | 14 | 1 | 18% | 2 | ni | ni | ni | ni | ni | ni |
| T-complex protein 1 subunit delta isoform b | 375477430 | 55 | ni | ni | ni | 3 | 7.50% | 3 | 3 | 8.10% | 5 |
| T-complex protein 1 subunit epsilon | 24307939 | 60 | ni | ni | ni | 3 | 13% | 6 | 2 | 8.50% | 4 |
| calpain small subunit 1 | 51599151 | 28 | ni | ni | ni | 2 | 17% | 3 | 2 | 6.30% | 4 |
| peroxiredoxin-5, mitochondrial isoform a precursor | 6912238 | 22 | ni | ni | ni | 2 | 18% | 2 | 2 | 13% | 4 |
| tomoregulin-2 precursor | 12383051 | 41 | ni | ni | ni | 2 | 5.90% | 5 | 2 | 6.70% | 6 |
| importin-5 isoform X2 | 530423350 | 126 | ni | ni | ni | 2 | 2.80% | 3 | 1 | 1.80% | 2 |
| reticulon-4 receptor-like 1 precursor | 30425553 | 49 | ni | ni | ni | 2 | 6.30% | 3 | 1 | 3.20% | 3 |
| epithelial discoidin domain-containing receptor 1 isoform 1 precursor | 38327632 | 97 | ni | ni | ni | 2 | 3.10% | 6 | 1 | 1.00% | 2 |
| transcobalamin-2 isoform 1 precursor | 21071010 | 48 | ni | ni | ni | 2 | 8.00% | 3 | 1 | 6.60% | 1 |
| delta-aminolevulinic acid dehydratase | 189083849 | 36 | ni | ni | ni | 2 | 8.80% | 3 | 1 | 5.80% | 3 |
| 2',3'-cyclic-nucleotide 3'-phosphodiesterase isoform X1 | 578830418 | 45 | ni | ni | ni | 2 | 12% | 3 | 1 | 6.20% | 3 |
| collagen alpha-1(XXIII) chain isoform X3 | 578811278 | 53 | ni | ni | ni | 2 | 4.90% | 3 | ni | ni | ni |
| annexin A7 isoform 1 | 4502111 | 50 | ni | ni | ni | 2 | 5.80% | 2 | ni | ni | ni |
| desmoglein-1 preproprotein | 119703744 | 114 | ni | ni | ni | 2 | 3.80% | 2 | ni | ni | ni |
| GTP-binding nuclear protein Ran | 5453555 | 24 | ni | ni | ni | 1 | 6.50% | 2 | 2 | 11% | 6 |
| disintegrin and metalloproteinase domain-containing protein 7 preproprotein | 114326453 | 86 | ni | ni | ni | 1 | 3.20% | 1 | 2 | 2.50% | 3 |
| N-acetylgalactosamine-6-sulfatase isoform X2 | 530424709 | 59 | ni | ni | ni | 1 | 4.20% | 3 | 2 | 4.70% | 3 |
| protein DPCD | 39930355 | 23 | ni | ni | ni | 1 | 5.40% | 2 | 2 | 10% | 5 |
| 14-3-3 protein gamma | 21464101 | 28 | ni | ni | ni | 1 | 13% | 3 | 2 | 17% | 6 |
| basal cell adhesion molecule isoform 1 precursor | 31543106 | 67 | ni | ni | ni | 1 | 2.10% | 1 | 2 | 4.30% | 6 |
| ly6/PLAUR domain-containing protein 3 precursor | 93004088 | 36 | ni | ni | ni | 1 | 5.80% | 2 | 2 | 8.70% | 2 |
| low-density lipoprotein receptor isoform 2 precursor | 307775412 | 95 | ni | ni | ni | 1 | 1.90% | 2 | 2 | 3.40% | 2 |
| arachidonate 15-lipoxygenase B isoform a | 85067499 | 73 | ni | ni | ni | 1 | 4.80% | 1 | 2 | 5.30% | 2 |
| sortilin isoform 1 preproprotein | 17149834 | 92 | ni | ni | ni | 1 | 1.80% | 1 | 1 | 1.80% | 1 |
| complement C1q tumor necrosis factor-related protein 1 isoform X1 | 578830330 | 42 | ni | ni | ni | 1 | 2.60% | 3 | 1 | 2.60% | 2 |
| lysozyme-like protein 4 precursor | 21389465 | 16 | ni | ni | ni | 1 | 6.80% | 2 | 1 | 8.20% | 1 |
| purine nucleoside phosphorylase | 157168362 | 32 | ni | ni | ni | 1 | 6.20% | 3 | 1 | 3.50% | 1 |
| rab GDP dissociation inhibitor alpha | 4503971 | 51 | ni | ni | ni | 1 | 13% | 1 | 1 | 18% | 2 |
| thioredoxin domain-containing protein 16 isoform 1 precursor | 237648959 | 94 | ni | ni | ni | 1 | 2.90% | 1 | 1 | 1.50% | 1 |
| brain-specific serine protease 4 isoform X1 | 530408666 | 34 | ni | ni | ni | 1 | 4.10% | 1 | 1 | 6.30% | 1 |
| UTP--glucose-1-phosphate uridylyltransferase isoform a | 48255966 | 57 | ni | ni | ni | 1 | 1.80% | 1 | ni | ni | ni |
| plakophilin-1 isoform 1a | 53729344 | 80 | ni | ni | ni | 1 | 2.60% | 2 | ni | ni | ni |
| tripeptidyl-peptidase 2 | 186972143 | 138 | ni | ni | ni | 1 | 1.80% | 1 | ni | ni | ni |
| stress-induced-phosphoprotein 1 isoform a | 544063423 | 68 | ni | ni | ni | 1 | 2.20% | 1 | ni | ni | ni |
| iduronate 2-sulfatase isoform c | 262118210 | 52 | ni | ni | ni | 1 | 4.10% | 1 | ni | ni | ni |
| leucine-rich repeat-containing protein 37A3 precursor | 75677612 | 181 | ni | ni | ni | 1 | 0.92% | 1 | ni | ni | ni |
| CDGSH iron-sulfur domain-containing protein 1 | 8923930 | 12 | ni | ni | ni | 1 | 20% | 2 | ni | ni | ni |
| tumor necrosis factor ligand superfamily member 10 isoform 1 | 4507593 | 33 | ni | ni | ni | 1 | 4.30% | 1 | ni | ni | ni |
| vesicular integral-membrane protein VIP36 precursor | 5803023 | 40 | ni | ni | ni | 1 | 5.90% | 1 | ni | ni | ni |
| phosphoglycolate phosphatase | 108796653 | 34 | ni | ni | ni | ni | ni | ni | 3 | 17% | 5 |
| valine--tRNA ligase isoform X1 | 530382523 | 141 | ni | ni | ni | ni | ni | ni | 2 | 4.40% | 2 |
| retinol-binding protein 4 precursor | 55743122 | 23 | ni | ni | ni | ni | ni | ni | 2 | 10.00% | 2 |
| pancreatic secretory granule membrane major glycoprotein GP2 isoform X2 | 530407931 | 73 | ni | ni | ni | ni | ni | ni | 2 | 4.10% | 3 |
| collagen alpha-1(VI) chain precursor | 87196339 | 109 | ni | ni | ni | ni | ni | ni | 2 | 2.20% | 4 |
| 14-3-3 protein sigma | 5454052 | 28 | ni | ni | ni | ni | ni | ni | 2 | 17% | 3 |
| receptor-type tyrosine-protein phosphatase F isoform 2 precursor | 109633039 | 212 | ni | ni | ni | ni | ni | ni | 2 | 2.20% | 2 |
| dyslexia-associated protein KIAA0319-like protein isoform X3 | 578799789 | 116 | ni | ni | ni | ni | ni | ni | 1 | 1.60% | 1 |
| tryptophan--tRNA ligase, cytoplasmic isoform X4 | 578826102 | 53 | ni | ni | ni | ni | ni | ni | 1 | 4.50% | 1 |
| beta-1,4-galactosyltransferase 4 isoform X4 | 578807844 | 40 | ni | ni | ni | ni | ni | ni | 1 | 5.20% | 2 |
| histone H2A type 1-B/E | 10645195 | 14 | ni | ni | ni | ni | ni | ni | 1 | 6.90% | 1 |
| lipase member I | 39752679 | 55 | ni | ni | ni | ni | ni | ni | 1 | 2.50% | 1 |
| desmocollin-1 isoform Dsc1b preproprotein | 4826702 | 94 | ni | ni | ni | ni | ni | ni | 1 | 1.80% | 1 |
| protein-L-isoaspartate(D-aspartate) O-methyltransferase isoform 1 | 226530908 | 30 | ni | ni | ni | ni | ni | ni | 1 | 6.00% | 1 |
| nicotinate-nucleotide pyrophosphorylase [carboxylating] isoform X1 | 530407851 | 31 | ni | ni | ni | ni | ni | ni | 1 | 13% | 2 |
| sperm acrosome membrane-associated protein 4 precursor | 19424138 | 13 | ni | ni | ni | ni | ni | ni | 1 | 10% | 1 |
| acyl-protein thioesterase 1 isoform 3 | 525342581 | 23 | ni | ni | ni | ni | ni | ni | 1 | 6.50% | 2 |
