# Supplementary material for: Comparative proteomic network signatures in seminal plasma of infertile men as a function of reactive oxygen species
Source: Clin Proteomics. 2015 Aug 28;12(1):23. doi: 10.1186/s12014-015-9094-5 (PMC4552280; doi:10.1186/s12014-015-9094-5)
Supplement: Additional file 4: — Table S4. Identified proteins in seminal plasma of infertile men with High ROS levels [file 12014_2015_9094_MOESM4_ESM.docx]

| **Table S4. Identified proteins in seminal plasma of infertile men with High ROS level** | | | | | | | | | | | | | | |
| --- | --- | --- | --- | --- | --- | --- | --- | --- | --- | --- | --- | --- | --- | --- |
| **Protein** | **Accession#** | **MW**  **kDa** | **Replicate 1** | | | **Replicate 2** | | | | | **Replicate 3** | | | |
|  |  |  | Peptides | Coverage | Spectral Counts | Peptides | | Coverage | | Spectral Counts | Peptides | | Coverage | Spectral Counts |
| fibronectin isoform 3 preproprotein | 16933542 | 259 | 126 | 56% | 1226 | 141 | 64% | | 1323 | | 151 | 59% | | 1303 |
| serum albumin preproprotein | 4502027 | 69 | 115 | 83% | 1784 | 100 | 84% | | 2180 | | 108 | 85% | | 1803 |
| lactotransferrin isoform 1 precursor | 54607120 | 78 | 86 | 84% | 2377 | 81 | 84% | | 1841 | | 81 | 85% | | 1630 |
| mucin-6 precursor | 151301154 | 257 | 58 | 27% | 637 | 68 | 29% | | 761 | | 58 | 26% | | 681 |
| aminopeptidase N isoform X1 | 530407092 | 110 | 54 | 59% | 403 | 60 | 60% | | 398 | | 61 | 61% | | 432 |
| semenogelin-2 precursor | 4506885 | 65 | 49 | 56% | 832 | 56 | 56% | | 679 | | 43 | 50% | | 547 |
| serotransferrin precursor | 4557871 | 77 | 49 | 72% | 349 | 51 | 70% | | 396 | | 51 | 72% | | 386 |
| prostatic acid phosphatase isoform PAP precursor | 6382064 | 45 | 46 | 72% | 754 | 53 | 72% | | 1019 | | 53 | 73% | | 807 |
| laminin subunit alpha-5 precursor | 21264602 | 400 | 41 | 16% | 93 | 36 | 15% | | 76 | | 39 | 16% | | 90 |
| IgGFc-binding protein precursor | 154146262 | 572 | 40 | 12% | 125 | 39 | 12% | | 136 | | 35 | 11% | | 115 |
| plastin-2 isoform X2 | 530402335 | 70 | 35 | 67% | 108 | 33 | 65% | | 135 | | 37 | 73% | | 127 |
| prostate-specific antigen isoform 1 preproprotein | 4502173 | 29 | 33 | 85% | 749 | 37 | 85% | | 909 | | 34 | 84% | | 690 |
| angiotensin-converting enzyme isoform 1 precursor | 4503273 | 150 | 33 | 33% | 116 | 36 | 32% | | 140 | | 40 | 35% | | 150 |
| zinc-alpha-2-glycoprotein precursor | 4502337 | 34 | 33 | 66% | 305 | 30 | 63% | | 378 | | 35 | 66% | | 329 |
| prosaposin isoform b preproprotein | 110224476 | 58 | 33 | 58% | 199 | 30 | 63% | | 235 | | 32 | 63% | | 217 |
| semenogelin-1 preproprotein | 4506883 | 52 | 32 | 63% | 333 | 35 | 66% | | 353 | | 28 | 60% | | 233 |
| laminin subunit beta-2 isoform X1 | 530372442 | 196 | 31 | 22% | 127 | 32 | 22% | | 129 | | 31 | 22% | | 128 |
| alpha-1-antitrypsin precursor | 189163532 | 47 | 29 | 61% | 190 | 31 | 63% | | 254 | | 31 | 61% | | 225 |
| neprilysin isoform X1 | 578807443 | 86 | 29 | 52% | 103 | 29 | 51% | | 130 | | 30 | 54% | | 106 |
| clusterin preproprotein | 355594753 | 52 | 29 | 46% | 339 | 27 | 46% | | 405 | | 30 | 48% | | 377 |
| heat shock protein HSP 90-alpha isoform 1 | 153792590 | 98 | 27 | 38% | 270 | 29 | 34% | | 207 | | 29 | 38% | | 222 |
| extracellular matrix protein 1 isoform 1 precursor | 221316614 | 61 | 27 | 59% | 177 | 29 | 61% | | 225 | | 28 | 61% | | 206 |
| plasma serine protease inhibitor preproprotein | 194018472 | 46 | 26 | 65% | 244 | 24 | 62% | | 271 | | 25 | 65% | | 257 |
| sulfhydryl oxidase 1 isoform a precursor | 13325075 | 83 | 25 | 40% | 96 | 24 | 38% | | 118 | | 28 | 42% | | 119 |
| protein-glutamine gamma-glutamyltransferase 4 | 156627577 | 77 | 25 | 46% | 99 | 22 | 41% | | 106 | | 27 | 57% | | 99 |
| complement C3 precursor | 115298678 | 187 | 24 | 22% | 57 | 23 | 21% | | 62 | | 26 | 24% | | 69 |
| beta-hexosaminidase subunit beta preproprotein | 4504373 | 63 | 24 | 44% | 87 | 20 | 39% | | 81 | | 24 | 43% | | 100 |
| alpha-2-macroglobulin isoform X1 | 578822814 | 167 | 23 | 18% | 75 | 31 | 25% | | 103 | | 30 | 23% | | 110 |
| dipeptidyl peptidase 4 | 18765694 | 88 | 23 | 30% | 92 | 29 | 46% | | 123 | | 27 | 35% | | 112 |
| prolactin-inducible protein precursor | 4505821 | 17 | 22 | 74% | 1412 | 35 | 77% | | 1584 | | 23 | 77% | | 320 |
| actin, cytoplasmic 1 | 4501885 | 42 | 22 | 66% | 319 | 23 | 67% | | 234 | | 22 | 71% | | 256 |
| heat shock-related 70 protein 2 | 13676857 | 70 | 22 | 45% | 148 | 19 | 32% | | 118 | | 22 | 36% | | 145 |
| matrix-remodeling-associated protein 5 isoform X1 | 530421042 | 315 | 22 | 10% | 49 | 18 | 8.90% | | 48 | | 21 | 9.00% | | 51 |
| endoplasmin precursor | 4507677 | 92 | 21 | 27% | 232 | 20 | 26% | | 163 | | 20 | 30% | | 217 |
| carboxypeptidase E preproprotein | 4503009 | 53 | 21 | 50% | 145 | 20 | 49% | | 166 | | 18 | 49% | | 117 |
| matrilin-2 isoform a precursor | 62548860 | 107 | 21 | 29% | 75 | 17 | 27% | | 70 | | 20 | 31% | | 70 |
| cartilage acidic protein 1 isoform B precursor | 330688397 | 70 | 20 | 48% | 71 | 21 | 57% | | 78 | | 20 | 42% | | 74 |
| L-lactate dehydrogenase C chain | 9257228 | 36 | 20 | 53% | 192 | 17 | 60% | | 139 | | 21 | 63% | | 177 |
| 78 glucose-regulated protein precursor | 16507237 | 72 | 19 | 38% | 144 | 20 | 36% | | 82 | | 17 | 38% | | 84 |
| isocitrate dehydrogenase [NADP] cytoplasmic | 538917681 | 47 | 19 | 52% | 85 | 19 | 58% | | 97 | | 22 | 59% | | 119 |
| lipoprotein lipase precursor | 4557727 | 53 | 19 | 43% | 107 | 19 | 50% | | 129 | | 17 | 47% | | 93 |
| trifunctional enzyme subunit alpha, mitochondrial precursor | 20127408 | 83 | 19 | 44% | 205 | 15 | 36% | | 121 | | 15 | 37% | | 136 |
| galectin-3-binding protein precursor | 5031863 | 65 | 18 | 29% | 140 | 21 | 33% | | 187 | | 21 | 40% | | 186 |
| nucleobindin-2 isoform X1 | 578820554 | 50 | 18 | 52% | 67 | 16 | 51% | | 72 | | 17 | 51% | | 68 |
| programmed cell death 6-interacting protein isoform 1 | 22027538 | 96 | 17 | 35% | 52 | 23 | 41% | | 60 | | 22 | 39% | | 65 |
| agrin precursor | 54873613 | 215 | 17 | 6.70% | 43 | 22 | 10% | | 46 | | 19 | 9.30% | | 42 |
| alpha-enolase isoform 1 | 4503571 | 47 | 17 | 53% | 114 | 18 | 55% | | 107 | | 18 | 55% | | 99 |
| heat shock 70 protein 1A/1B | 167466173 | 70 | 17 | 37% | 64 | 17 | 41% | | 61 | | 18 | 41% | | 60 |
| kallistatin isoform 2 precursor | 21361302 | 49 | 17 | 55% | 52 | 16 | 55% | | 58 | | 16 | 49% | | 51 |
| carboxypeptidase Z isoform 1 precursor | 62388877 | 74 | 17 | 36% | 70 | 14 | 37% | | 52 | | 16 | 40% | | 60 |
| laminin subunit gamma-1 precursor | 145309326 | 178 | 16 | 14% | 47 | 17 | 16% | | 42 | | 22 | 19% | | 55 |
| mucin-5B precursor | 301172750 | 596 | 16 | 3.30% | 42 | 17 | 3.60% | | 57 | | 19 | 4.40% | | 48 |
| ATP synthase subunit beta, mitochondrial precursor | 32189394 | 57 | 16 | 50% | 205 | 17 | 52% | | 115 | | 16 | 50% | | 128 |
| tubulin alpha-3C/D chain | 156564363 | 50 | 15 | 51% | 111 | 16 | 56% | | 95 | | 15 | 55% | | 102 |
| cathepsin B preproprotein | 22538431 | 38 | 15 | 48% | 69 | 14 | 51% | | 62 | | 16 | 49% | | 77 |
| hemopexin precursor | 11321561 | 52 | 15 | 52% | 55 | 13 | 47% | | 74 | | 16 | 53% | | 72 |
| tubulin beta-4B chain | 5174735 | 50 | 15 | 63% | 71 | 13 | 51% | | 67 | | 15 | 58% | | 72 |
| metalloproteinase inhibitor 2 precursor | 4507511 | 24 | 15 | 66% | 38 | 12 | 54% | | 31 | | 8 | 41% | | 15 |
| polymeric immunoglobulin receptor isoform X1 | 530366266 | 85 | 14 | 26% | 48 | 16 | 29% | | 57 | | 14 | 26% | | 51 |
| ATP synthase subunit alpha, mitochondrial isoform c | 382546190 | 54 | 14 | 40% | 102 | 16 | 49% | | 71 | | 14 | 41% | | 77 |
| maltase-glucoamylase, intestinal isoform X1 | 578814724 | 312 | 14 | 7.10% | 33 | 15 | 8.10% | | 34 | | 22 | 11% | | 56 |
| epididymal secretory protein E1 precursor | 5453678 | 17 | 14 | 63% | 151 | 15 | 67% | | 184 | | 15 | 56% | | 104 |
| fructose-bisphosphate aldolase A isoform 2 | 342187211 | 45 | 14 | 56% | 64 | 14 | 56% | | 79 | | 17 | 64% | | 88 |
| beta-mannosidase precursor | 84798622 | 101 | 14 | 23% | 34 | 14 | 22% | | 37 | | 16 | 25% | | 35 |
| glucose-6-phosphate isomerase isoform X2 | 530416229 | 63 | 14 | 34% | 63 | 13 | 42% | | 51 | | 16 | 45% | | 57 |
| saccharopine dehydrogenase-like oxidoreductase | 55770836 | 47 | 14 | 62% | 119 | 12 | 49% | | 86 | | 16 | 57% | | 154 |
| alpha-1-antichymotrypsin precursor | 50659080 | 48 | 14 | 42% | 71 | 11 | 33% | | 75 | | 16 | 44% | | 86 |
| pyruvate kinase PKM isoform X1 | 530405975 | 65 | 13 | 28% | 57 | 16 | 35% | | 58 | | 15 | 32% | | 49 |
| cysteine-rich secretory protein 1 isoform 1 precursor | 25121982 | 28 | 13 | 67% | 110 | 15 | 71% | | 138 | | 16 | 67% | | 118 |
| acid ceramidase isoform b | 189011546 | 47 | 13 | 39% | 65 | 14 | 39% | | 55 | | 11 | 39% | | 44 |
| beta-hexosaminidase subunit alpha preproprotein | 189181666 | 61 | 13 | 38% | 37 | 13 | 36% | | 44 | | 14 | 42% | | 43 |
| cathepsin D preproprotein | 4503143 | 45 | 13 | 47% | 76 | 12 | 42% | | 77 | | 13 | 49% | | 76 |
| glutathione S-transferase Mu 3 | 23065552 | 27 | 13 | 54% | 48 | 12 | 60% | | 38 | | 8 | 44% | | 30 |
| 60 heat shock protein, mitochondrial isoform X1 | 530370277 | 61 | 13 | 32% | 67 | 11 | 29% | | 62 | | 11 | 28% | | 74 |
| cytosolic non-specific dipeptidase isoform X2 | 530414265 | 53 | 13 | 34% | 50 | 10 | 28% | | 35 | | 14 | 42% | | 40 |
| alpha-N-acetylglucosaminidase precursor | 66346698 | 82 | 12 | 25% | 32 | 16 | 32% | | 39 | | 15 | 32% | | 37 |
| cation-independent mannose-6-phosphate receptor precursor | 119964726 | 274 | 12 | 6.20% | 28 | 15 | 6.30% | | 34 | | 19 | 9.20% | | 46 |
| zona pellucida-binding protein 1 isoform 1 precursor | 229577313 | 40 | 12 | 38% | 175 | 15 | 38% | | 110 | | 13 | 37% | | 129 |
| procollagen-lysine,2-oxoglutarate 5-dioxygenase 1 precursor | 32307144 | 84 | 12 | 24% | 25 | 15 | 32% | | 40 | | 11 | 23% | | 29 |
| peptidyl-prolyl cis-trans isomerase B precursor | 4758950 | 24 | 12 | 52% | 68 | 15 | 59% | | 75 | | 10 | 48% | | 40 |
| sorbitol dehydrogenase | 156627571 | 38 | 12 | 51% | 55 | 13 | 58% | | 73 | | 12 | 45% | | 52 |
| rab GDP dissociation inhibitor beta isoform 1 | 6598323 | 51 | 12 | 33% | 33 | 11 | 41% | | 35 | | 13 | 46% | | 35 |
| plasma protease C1 inhibitor precursor | 73858568 | 55 | 12 | 26% | 44 | 11 | 24% | | 44 | | 12 | 25% | | 48 |
| cytosol aminopeptidase | 41393561 | 56 | 12 | 28% | 69 | 11 | 29% | | 50 | | 9 | 29% | | 66 |
| sialate O-acetylesterase isoform 1 precursor | 24850115 | 58 | 12 | 39% | 47 | 10 | 38% | | 43 | | 11 | 37% | | 44 |
| lysosomal alpha-glucosidase isoform X1 | 530411863 | 105 | 11 | 21% | 40 | 16 | 30% | | 56 | | 13 | 25% | | 36 |
| growth arrest-specific protein 6 isoform 1 precursor | 4557617 | 75 | 11 | 24% | 29 | 13 | 26% | | 42 | | 11 | 25% | | 37 |
| alpha-2-antiplasmin isoform X1 | 530410436 | 62 | 11 | 28% | 40 | 12 | 37% | | 62 | | 12 | 35% | | 39 |
| malate dehydrogenase, mitochondrial isoform 1 precursor | 21735621 | 36 | 11 | 49% | 55 | 12 | 48% | | 42 | | 12 | 45% | | 55 |
| glyceraldehyde-3-phosphate dehydrogenase isoform 1 | 576583524 | 36 | 11 | 43% | 83 | 12 | 53% | | 77 | | 11 | 42% | | 59 |
| carboxylesterase 5A isoform 1 precursor | 219521907 | 64 | 11 | 30% | 37 | 12 | 31% | | 37 | | 11 | 27% | | 32 |
| vitamin D-binding protein isoform 3 precursor | 324021745 | 55 | 11 | 37% | 36 | 11 | 40% | | 31 | | 13 | 41% | | 35 |
| von Willebrand factor A domain-containing protein 1 isoform 1 precursor | 40068485 | 47 | 11 | 47% | 55 | 11 | 44% | | 65 | | 11 | 47% | | 60 |
| transmembrane protease serine 2 isoform 2 | 205360943 | 54 | 11 | 39% | 49 | 11 | 44% | | 50 | | 10 | 29% | | 35 |
| receptor-type tyrosine-protein phosphatase S isoform X1 | 530425335 | 215 | 11 | 8.40% | 38 | 11 | 8.30% | | 34 | | 8 | 5.30% | | 23 |
| attractin isoform 1 preproprotein | 21450861 | 159 | 11 | 11% | 28 | 10 | 7.60% | | 26 | | 13 | 9.70% | | 38 |
| T-complex protein 1 subunit eta isoform a | 5453607 | 59 | 11 | 29% | 62 | 10 | 29% | | 48 | | 9 | 29% | | 51 |
| acrosin-binding protein precursor | 17999524 | 61 | 11 | 35% | 51 | 10 | 30% | | 55 | | 7 | 23% | | 34 |
| dipeptidase 3 isoform a precursor | 193211608 | 56 | 11 | 20% | 29 | 10 | 20% | | 29 | | 7 | 16% | | 29 |
| alpha-1B-glycoprotein precursor | 21071030 | 54 | 11 | 39% | 28 | 9 | 33% | | 34 | | 13 | 48% | | 37 |
| aldose reductase | 4502049 | 36 | 11 | 59% | 33 | 9 | 47% | | 25 | | 12 | 62% | | 36 |
| A disintegrin and metalloproteinase with thrombospondin motifs 1 preproprotein | 50845384 | 105 | 11 | 16% | 32 | 9 | 13% | | 25 | | 10 | 16% | | 33 |
| serpin B6 isoform c | 425876766 | 44 | 11 | 36% | 31 | 9 | 32% | | 31 | | 10 | 34% | | 35 |
| fumarate hydratase, mitochondrial | 19743875 | 55 | 11 | 45% | 50 | 8 | 34% | | 45 | | 9 | 39% | | 55 |
| aconitate hydratase, mitochondrial precursor | 4501867 | 85 | 11 | 31% | 36 | 6 | 19% | | 18 | | 5 | 13% | | 22 |
| neutral alpha-glucosidase AB isoform 2 precursor | 38202257 | 107 | 10 | 17% | 45 | 13 | 18% | | 52 | | 10 | 16% | | 43 |
| triosephosphate isomerase isoform 2 | 226529917 | 31 | 10 | 58% | 36 | 12 | 62% | | 46 | | 11 | 57% | | 30 |
| procollagen-lysine,2-oxoglutarate 5-dioxygenase 3 precursor | 4505891 | 85 | 10 | 20% | 23 | 12 | 23% | | 28 | | 10 | 20% | | 26 |
| tripeptidyl-peptidase 1 preproprotein | 5729770 | 61 | 10 | 33% | 32 | 11 | 43% | | 36 | | 12 | 45% | | 39 |
| ribonuclease T2 precursor | 5231228 | 29 | 10 | 53% | 43 | 11 | 49% | | 61 | | 11 | 53% | | 54 |
| protein DJ-1 isoform X1 | 530360487 | 20 | 10 | 72% | 44 | 11 | 76% | | 47 | | 9 | 59% | | 24 |
| olfactomedin-4 precursor | 32313593 | 57 | 10 | 27% | 34 | 11 | 35% | | 38 | | 9 | 25% | | 33 |
| annexin A5 | 4502107 | 36 | 10 | 35% | 26 | 10 | 42% | | 31 | | 13 | 51% | | 29 |
| phospholipase A1 member A isoform 2 precursor | 332688256 | 48 | 10 | 36% | 54 | 9 | 37% | | 61 | | 10 | 35% | | 50 |
| transitional endoplasmic reticulum ATPase | 6005942 | 89 | 10 | 16% | 31 | 9 | 19% | | 29 | | 9 | 17% | | 24 |
| alpha-actinin-4 isoform X3 | 578834952 | 105 | 10 | 18% | 21 | 8 | 12% | | 21 | | 9 | 15% | | 24 |
| amiloride-sensitive amine oxidase [copper-containing] isoform 1 precursor | 440918691 | 87 | 9 | 16% | 25 | 14 | 30% | | 38 | | 12 | 22% | | 33 |
| nucleotide exchange factor SIL1 precursor | 11968009 | 52 | 9 | 27% | 31 | 13 | 33% | | 51 | | 11 | 30% | | 45 |
| protein disulfide-isomerase A3 precursor | 21361657 | 57 | 9 | 22% | 42 | 12 | 33% | | 49 | | 13 | 26% | | 51 |
| tissue alpha-L-fucosidase precursor | 119360348 | 54 | 9 | 32% | 29 | 12 | 47% | | 59 | | 11 | 33% | | 39 |
| beta-galactosidase isoform b | 119372312 | 73 | 9 | 23% | 20 | 12 | 25% | | 36 | | 11 | 26% | | 33 |
| cathelicidin antimicrobial peptide preproprotein | 348041314 | 20 | 9 | 42% | 40 | 12 | 50% | | 57 | | 7 | 43% | | 29 |
| creatine kinase B-type | 21536286 | 43 | 9 | 45% | 44 | 11 | 51% | | 51 | | 11 | 50% | | 39 |
| di-N-acetylchitobiase precursor | 4758092 | 44 | 9 | 37% | 26 | 11 | 43% | | 32 | | 9 | 39% | | 24 |
| T-complex protein 1 subunit beta isoform 1 | 5453603 | 57 | 9 | 29% | 75 | 10 | 29% | | 55 | | 10 | 29% | | 81 |
| legumain preproprotein | 56682962 | 49 | 9 | 32% | 42 | 9 | 33% | | 53 | | 9 | 32% | | 39 |
| leucine-rich alpha-2-glycoprotein precursor | 16418467 | 38 | 9 | 41% | 36 | 9 | 40% | | 47 | | 9 | 40% | | 42 |
| prostasin preproprotein | 4506153 | 36 | 9 | 35% | 28 | 9 | 41% | | 36 | | 9 | 41% | | 29 |
| cystatin-S precursor | 4503109 | 16 | 9 | 67% | 40 | 9 | 67% | | 44 | | 6 | 54% | | 15 |
| metalloproteinase inhibitor 1 precursor | 4507509 | 23 | 9 | 67% | 89 | 8 | 57% | | 79 | | 10 | 67% | | 85 |
| CD177 antigen precursor | 110735433 | 46 | 9 | 39% | 48 | 8 | 37% | | 47 | | 10 | 37% | | 52 |
| Golgi apparatus protein 1 isoform 2 precursor | 224586815 | 136 | 9 | 7.70% | 26 | 8 | 9.60% | | 24 | | 10 | 9.60% | | 32 |
| elongation factor 1-alpha 1 | 4503471 | 50 | 9 | 24% | 43 | 8 | 30% | | 48 | | 9 | 32% | | 53 |
| annexin A1 | 4502101 | 39 | 9 | 36% | 22 | 8 | 32% | | 27 | | 9 | 35% | | 27 |
| 2,4-dienoyl-CoA reductase, mitochondrial precursor | 4503301 | 36 | 9 | 36% | 93 | 8 | 42% | | 75 | | 8 | 38% | | 118 |
| succinyl-CoA:3-ketoacid coenzyme A transferase 1, mitochondrial precursor | 4557817 | 56 | 9 | 34% | 94 | 8 | 29% | | 60 | | 8 | 31% | | 82 |
| protein disulfide-isomerase precursor | 20070125 | 57 | 9 | 26% | 41 | 7 | 20% | | 30 | | 9 | 25% | | 32 |
| retinoid-inducible serine carboxypeptidase precursor | 11055992 | 51 | 9 | 27% | 24 | 7 | 20% | | 17 | | 9 | 27% | | 28 |
| monocyte differentiation antigen CD14 precursor | 291575163 | 40 | 9 | 33% | 27 | 7 | 24% | | 23 | | 7 | 27% | | 19 |
| succinate dehydrogenase [ubiquinone] flavoprotein subunit, mitochondrial | 156416003 | 73 | 9 | 31% | 54 | 7 | 28% | | 38 | | 7 | 24% | | 39 |
| hexokinase-1 isoform HKI-R | 15991827 | 102 | 9 | 18% | 46 | 7 | 14% | | 18 | | 6 | 12% | | 22 |
| importin subunit beta-1 isoform 2 | 449784879 | 81 | 9 | 23% | 36 | 5 | 18% | | 15 | | 7 | 15% | | 26 |
| beta-microseminoprotein isoform a precursor | 4557036 | 13 | 8 | 66% | 31 | 16 | 75% | | 61 | | 6 | 54% | | 23 |
| 72 type IV collagenase isoform a preproprotein | 11342666 | 74 | 8 | 21% | 31 | 13 | 30% | | 40 | | 14 | 37% | | 33 |
| annexin A3 isoform X1 | 530377641 | 36 | 8 | 31% | 23 | 11 | 44% | | 28 | | 8 | 32% | | 19 |
| 4-trimethylaminobutyraldehyde dehydrogenase | 115387104 | 56 | 8 | 28% | 40 | 11 | 24% | | 31 | | 8 | 20% | | 30 |
| annexin A2 isoform 2 | 50845386 | 39 | 8 | 35% | 23 | 11 | 44% | | 26 | | 8 | 30% | | 25 |
| collagen alpha-1(XVIII) chain isoform 2 precursor | 110611233 | 136 | 8 | 5.00% | 28 | 10 | 8.20% | | 30 | | 8 | 8.80% | | 26 |
| multiple inositol polyphosphate phosphatase 1 isoform 1 precursor | 19923761 | 55 | 8 | 30% | 19 | 9 | 32% | | 23 | | 11 | 40% | | 27 |
| cystatin-C precursor | 568599832 | 16 | 8 | 57% | 50 | 9 | 57% | | 64 | | 9 | 60% | | 42 |
| protein OS-9 isoform 1 precursor | 5803109 | 76 | 8 | 18% | 20 | 9 | 24% | | 27 | | 8 | 20% | | 19 |
| calsyntenin-1 isoform X1 | 530360505 | 108 | 8 | 15% | 15 | 9 | 14% | | 19 | | 8 | 15% | | 22 |
| malate dehydrogenase, cytoplasmic isoform 1 | 312283701 | 39 | 8 | 30% | 29 | 9 | 34% | | 29 | | 7 | 31% | | 25 |
| peroxiredoxin-1 | 320461711 | 22 | 8 | 41% | 25 | 9 | 46% | | 24 | | 7 | 37% | | 18 |
| phosphoglycerate kinase 1 | 4505763 | 45 | 8 | 36% | 25 | 8 | 42% | | 28 | | 9 | 43% | | 30 |
| arylsulfatase A isoform a precursor | 313569795 | 54 | 8 | 31% | 19 | 8 | 31% | | 22 | | 8 | 26% | | 22 |
| phosphatidylethanolamine-binding protein 1 preproprotein | 4505621 | 21 | 8 | 69% | 24 | 8 | 61% | | 25 | | 4 | 40% | | 12 |
| cullin-associated NEDD8-dissociated protein 1 | 21361794 | 136 | 8 | 11% | 20 | 7 | 9.70% | | 14 | | 9 | 13% | | 20 |
| epididymal sperm-binding protein 1 precursor | 301601648 | 26 | 8 | 43% | 48 | 7 | 47% | | 36 | | 8 | 52% | | 42 |
| acetyl-CoA acetyltransferase, mitochondrial precursor | 4557237 | 45 | 8 | 29% | 66 | 7 | 29% | | 43 | | 8 | 32% | | 67 |
| G-protein coupled receptor 64 isoform 2 precursor | 119943116 | 110 | 8 | 9.60% | 22 | 7 | 9.60% | | 23 | | 8 | 9.40% | | 34 |
| voltage-dependent calcium channel subunit alpha-2/delta-1 isoform X2 | 530386377 | 125 | 8 | 10% | 14 | 7 | 9.10% | | 15 | | 8 | 10% | | 19 |
| kallikrein-2 isoform 1 preproprotein | 5031829 | 29 | 8 | 64% | 37 | 7 | 59% | | 41 | | 7 | 55% | | 38 |
| protein FAM3B isoform a precursor | 46255030 | 26 | 8 | 43% | 37 | 7 | 36% | | 27 | | 7 | 30% | | 29 |
| 14-3-3 protein epsilon isoform X1 | 530410617 | 27 | 8 | 35% | 26 | 7 | 32% | | 29 | | 7 | 40% | | 22 |
| cadherin-1 preproprotein | 4757960 | 97 | 8 | 8.40% | 19 | 7 | 11% | | 22 | | 6 | 8.40% | | 17 |
| elongation factor 1-gamma | 4503481 | 50 | 8 | 31% | 38 | 6 | 27% | | 41 | | 8 | 32% | | 44 |
| heat shock protein HSP 90-beta isoform X1 | 530381931 | 83 | 8 | 28% | 21 | 6 | 25% | | 12 | | 7 | 29% | | 16 |
| adenosylhomocysteinase isoform 1 | 9951915 | 48 | 8 | 23% | 18 | 6 | 16% | | 10 | | 7 | 19% | | 19 |
| epididymal secretory protein E3-beta precursor | 11641279 | 18 | 8 | 59% | 41 | 6 | 44% | | 52 | | 4 | 37% | | 26 |
| proteasome subunit alpha type-6 isoform a | 23110944 | 27 | 8 | 39% | 20 | 6 | 23% | | 16 | | 3 | 13% | | 11 |
| glutamate carboxypeptidase 2 isoform 3 | 301500668 | 83 | 8 | 16% | 16 | 5 | 11% | | 13 | | 7 | 14% | | 18 |
| phosphoglycerate kinase 2 | 31543397 | 45 | 7 | 29% | 31 | 10 | 43% | | 39 | | 12 | 51% | | 38 |
| acrosin precursor | 148613878 | 46 | 7 | 21% | 42 | 10 | 28% | | 46 | | 10 | 30% | | 39 |
| carboxypeptidase M precursor | 53832021 | 51 | 7 | 12% | 21 | 9 | 18% | | 23 | | 9 | 14% | | 22 |
| elongation factor 2 | 4503483 | 95 | 7 | 7.80% | 18 | 9 | 13% | | 23 | | 9 | 11% | | 26 |
| prostaglandin-H2 D-isomerase precursor | 32171249 | 21 | 7 | 47% | 64 | 9 | 51% | | 83 | | 8 | 48% | | 57 |
| gastricsin isoform 1 preproprotein | 4505757 | 42 | 7 | 13% | 46 | 9 | 15% | | 69 | | 8 | 17% | | 56 |
| insulin-like growth factor-binding protein 2 precursor | 55925576 | 35 | 7 | 39% | 32 | 9 | 50% | | 46 | | 8 | 25% | | 30 |
| retinal dehydrogenase 1 | 21361176 | 55 | 7 | 23% | 18 | 9 | 28% | | 19 | | 8 | 26% | | 22 |
| cathepsin F precursor | 6042196 | 53 | 7 | 10% | 17 | 9 | 16% | | 23 | | 6 | 12% | | 14 |
| complement factor B preproprotein | 67782358 | 86 | 7 | 15% | 18 | 8 | 15% | | 25 | | 8 | 16% | | 20 |
| gamma-glutamyltranspeptidase 1 precursor | 572152963 | 61 | 7 | 11% | 24 | 8 | 16% | | 30 | | 6 | 11% | | 24 |
| alpha-1-acid glycoprotein 1 precursor | 167857790 | 24 | 7 | 41% | 28 | 7 | 41% | | 31 | | 8 | 41% | | 35 |
| phosphatidylethanolamine-binding protein 4 precursor | 116812622 | 26 | 7 | 59% | 39 | 7 | 43% | | 49 | | 6 | 42% | | 33 |
| extracellular superoxide dismutase [Cu-Zn] precursor | 118582275 | 26 | 7 | 54% | 39 | 7 | 54% | | 44 | | 6 | 50% | | 29 |
| sialidase-1 precursor | 4557791 | 45 | 7 | 26% | 18 | 7 | 23% | | 22 | | 5 | 14% | | 12 |
| proteasome subunit beta type-1 | 4506193 | 26 | 7 | 39% | 22 | 7 | 32% | | 26 | | 4 | 23% | | 10 |
| glutathione reductase, mitochondrial isoform 1 precursor | 50301238 | 56 | 7 | 18% | 16 | 6 | 16% | | 22 | | 12 | 33% | | 32 |
| dihydrolipoyl dehydrogenase, mitochondrial isoform 3 | 576583539 | 52 | 7 | 34% | 157 | 6 | 29% | | 111 | | 8 | 33% | | 178 |
| BPI fold-containing family B member 2 precursor | 15055535 | 49 | 7 | 23% | 20 | 6 | 21% | | 23 | | 6 | 21% | | 22 |
| 14-3-3 protein zeta/delta isoform X2 | 530389317 | 28 | 7 | 45% | 22 | 6 | 40% | | 22 | | 5 | 31% | | 21 |
| junction plakoglobin isoform X4 | 578830866 | 82 | 7 | 12% | 17 | 5 | 7.40% | | 11 | | 13 | 23% | | 25 |
| antileukoproteinase precursor | 4507065 | 14 | 7 | 52% | 28 | 5 | 45% | | 13 | | 5 | 33% | | 12 |
| peptidyl-prolyl cis-trans isomerase A | 10863927 | 18 | 7 | 55% | 23 | 5 | 44% | | 19 | | 4 | 25% | | 6 |
| nucleobindin-1 precursor | 20070228 | 54 | 7 | 18% | 15 | 4 | 13% | | 12 | | 5 | 16% | | 10 |
| dipeptidyl peptidase 2 preproprotein | 62420888 | 54 | 6 | 18% | 15 | 11 | 34% | | 27 | | 12 | 38% | | 36 |
| L-lactate dehydrogenase A chain isoform 1 | 5031857 | 37 | 6 | 33% | 18 | 11 | 45% | | 32 | | 11 | 43% | | 29 |
| peroxiredoxin-6 | 4758638 | 25 | 6 | 40% | 17 | 9 | 57% | | 31 | | 8 | 49% | | 18 |
| limbic system-associated membrane protein preproprotein | 45594240 | 37 | 6 | 27% | 25 | 9 | 33% | | 38 | | 6 | 26% | | 29 |
| cysteine-rich secretory protein LCCL domain-containing 2 precursor | 13899332 | 56 | 6 | 16% | 18 | 9 | 19% | | 26 | | 5 | 16% | | 15 |
| peroxiredoxin-2 | 32189392 | 22 | 6 | 36% | 19 | 9 | 34% | | 27 | | 5 | 25% | | 10 |
| glutathione S-transferase P | 4504183 | 23 | 6 | 51% | 22 | 9 | 60% | | 25 | | 4 | 30% | | 10 |
| complement C4-B-like preproprotein | 338858017 | 193 | 6 | 6.40% | 16 | 8 | 9.30% | | 20 | | 11 | 11% | | 21 |
| ras-related protein Rab-3B | 19923750 | 25 | 6 | 42% | 21 | 8 | 49% | | 23 | | 6 | 39% | | 12 |
| cysteine-rich secretory protein LCCL domain-containing 1 isoform 1 precursor | 13899303 | 57 | 6 | 23% | 16 | 8 | 29% | | 20 | | 1 | 2.20% | | 3 |
| fatty acid synthase | 41872631 | 273 | 6 | 2.00% | 14 | 7 | 3.60% | | 13 | | 11 | 4.60% | | 22 |
| L-lactate dehydrogenase B chain | 291575128 | 37 | 6 | 31% | 18 | 7 | 32% | | 17 | | 9 | 36% | | 25 |
| ruvB-like 2 | 5730023 | 51 | 6 | 24% | 18 | 7 | 27% | | 28 | | 7 | 27% | | 28 |
| ribonuclease 4 precursor | 37577172 | 17 | 6 | 35% | 22 | 7 | 35% | | 29 | | 5 | 29% | | 20 |
| beta-2-glycoprotein 1 precursor | 153266841 | 38 | 6 | 30% | 16 | 7 | 35% | | 17 | | 5 | 23% | | 15 |
| ras-related protein Rab-27A | 19923264 | 25 | 6 | 29% | 14 | 7 | 37% | | 21 | | 5 | 25% | | 13 |
| syntenin-1 isoform 1 | 55749490 | 32 | 6 | 42% | 23 | 7 | 43% | | 28 | | 4 | 27% | | 13 |
| prostate and testis expressed protein 1 precursor | 19923082 | 14 | 6 | 45% | 16 | 7 | 53% | | 21 | | 2 | 16% | | 5 |
| sperm acrosome membrane-associated protein 1 precursor | 13569934 | 32 | 6 | 28% | 44 | 6 | 21% | | 38 | | 7 | 21% | | 48 |
| protein MENT isoform X1 | 578801150 | 37 | 6 | 24% | 37 | 6 | 27% | | 29 | | 7 | 32% | | 33 |
| T-complex protein 1 subunit epsilon | 24307939 | 60 | 6 | 26% | 37 | 6 | 26% | | 29 | | 6 | 23% | | 31 |
| acylamino-acid-releasing enzyme isoform X2 | 530372382 | 70 | 6 | 13% | 14 | 6 | 13% | | 16 | | 6 | 8.10% | | 15 |
| lysosomal Pro-X carboxypeptidase isoform 1 preproprotein | 4826940 | 56 | 6 | 17% | 21 | 6 | 19% | | 17 | | 5 | 17% | | 17 |
| superoxide dismutase [Cu-Zn] | 4507149 | 16 | 6 | 60% | 27 | 6 | 64% | | 20 | | 3 | 54% | | 10 |
| NAD-dependent malic enzyme, mitochondrial isoform 1 precursor | 4505145 | 65 | 6 | 18% | 20 | 5 | 17% | | 18 | | 6 | 17% | | 21 |
| serum amyloid P-component precursor | 4502133 | 25 | 6 | 29% | 18 | 5 | 24% | | 15 | | 5 | 24% | | 11 |
| kunitz-type protease inhibitor 1 isoform 2 precursor | 4504329 | 57 | 6 | 11% | 17 | 5 | 9.40% | | 15 | | 3 | 5.50% | | 7 |
| leucine-rich repeat-containing protein 37B precursor | 53829385 | 106 | 6 | 14% | 39 | 3 | 9.60% | | 28 | | 5 | 13% | | 35 |
| cAMP-dependent protein kinase catalytic subunit alpha isoform 1 | 4506055 | 41 | 6 | 17% | 27 | 3 | 15% | | 8 | | 3 | 6.80% | | 13 |
| citrate synthase, mitochondrial precursor | 38327625 | 52 | 5 | 17% | 32 | 9 | 38% | | 31 | | 9 | 38% | | 34 |
| ezrin | 21614499 | 69 | 5 | 9.20% | 16 | 8 | 18% | | 19 | | 6 | 12% | | 20 |
| chitinase domain-containing protein 1 isoform X2 | 530395670 | 48 | 5 | 15% | 12 | 8 | 33% | | 22 | | 6 | 21% | | 15 |
| ADP-ribosyl cyclase 1 | 38454326 | 34 | 5 | 17% | 20 | 8 | 21% | | 31 | | 5 | 18% | | 16 |
| ceruloplasmin precursor | 4557485 | 122 | 5 | 4.80% | 19 | 7 | 7.20% | | 19 | | 8 | 8.70% | | 28 |
| beta-1,4-galactosyltransferase 1 | 13929462 | 44 | 5 | 20% | 14 | 7 | 34% | | 28 | | 6 | 27% | | 17 |
| aspartate aminotransferase, cytoplasmic | 4504067 | 46 | 5 | 17% | 18 | 7 | 26% | | 17 | | 5 | 23% | | 16 |
| dnaJ homolog subfamily C member 3 precursor | 5453980 | 58 | 5 | 16% | 18 | 7 | 24% | | 22 | | 5 | 15% | | 13 |
| WAP four-disulfide core domain protein 2 precursor | 56699495 | 13 | 5 | 45% | 37 | 7 | 49% | | 46 | | 4 | 45% | | 25 |
| neutrophil gelatinase-associated lipocalin precursor | 38455402 | 23 | 5 | 39% | 15 | 7 | 46% | | 23 | | 2 | 16% | | 5 |
| desmoplakin isoform I | 58530840 | 332 | 5 | 2.30% | 12 | 6 | 2.40% | | 12 | | 9 | 3.60% | | 15 |
| angiotensinogen preproprotein | 4557287 | 53 | 5 | 22% | 18 | 6 | 24% | | 18 | | 7 | 27% | | 20 |
| epididymal secretory protein E3-alpha precursor | 11386189 | 18 | 5 | 41% | 12 | 6 | 46% | | 17 | | 5 | 37% | | 11 |
| biotinidase isoform 3 | 4557373 | 61 | 5 | 12% | 22 | 6 | 16% | | 20 | | 5 | 12% | | 22 |
| carboxypeptidase Q isoform X1 | 530388680 | 52 | 5 | 18% | 12 | 6 | 23% | | 13 | | 5 | 18% | | 12 |
| cytochrome b-c1 complex subunit 1, mitochondrial precursor | 46593007 | 53 | 5 | 19% | 37 | 6 | 24% | | 28 | | 5 | 19% | | 24 |
| latent-transforming growth factor beta-binding protein 3 isoform 1 precursor | 194328809 | 139 | 5 | 4.30% | 9 | 6 | 5.00% | | 14 | | 3 | 2.20% | | 7 |
| proteasome subunit alpha type-2 | 4506181 | 26 | 5 | 38% | 19 | 6 | 44% | | 19 | | 2 | 15% | | 6 |
| hypoxia up-regulated protein 1 isoform X2 | 530397761 | 111 | 5 | 8.20% | 21 | 5 | 7.60% | | 17 | | 8 | 10% | | 22 |
| 6-phosphogluconate dehydrogenase, decarboxylating | 40068518 | 53 | 5 | 18% | 15 | 5 | 22% | | 19 | | 7 | 25% | | 18 |
| trifunctional enzyme subunit beta, mitochondrial isoform 1 precursor | 4504327 | 51 | 5 | 24% | 79 | 5 | 27% | | 42 | | 6 | 30% | | 69 |
| ceroid-lipofuscinosis neuronal protein 5 | 5729772 | 46 | 5 | 13% | 14 | 5 | 13% | | 11 | | 6 | 18% | | 15 |
| alpha-galactosidase A precursor | 4504009 | 49 | 5 | 16% | 21 | 5 | 16% | | 18 | | 5 | 16% | | 16 |
| histone H4 | 11415030 | 11 | 5 | 48% | 24 | 5 | 48% | | 23 | | 5 | 48% | | 20 |
| glycodelin precursor | 65507501 | 21 | 5 | 36% | 42 | 5 | 32% | | 46 | | 3 | 24% | | 31 |
| phosphoglycerate mutase 2 | 50593010 | 29 | 5 | 27% | 13 | 5 | 32% | | 11 | | 2 | 9.90% | | 7 |
| hemoglobin subunit beta | 4504349 | 16 | 5 | 43% | 13 | 5 | 43% | | 15 | | 2 | 16% | | 4 |
| lysosome-associated membrane glycoprotein 1 precursor | 112380628 | 45 | 5 | 9.10% | 13 | 4 | 8.60% | | 13 | | 5 | 11% | | 14 |
| cullin-3 isoform 1 | 4503165 | 89 | 5 | 7.00% | 16 | 4 | 4.70% | | 13 | | 5 | 6.00% | | 14 |
| immunoglobulin lambda-like polypeptide 5 isoform 1 | 295986608 | 23 | 5 | 36% | 24 | 4 | 27% | | 29 | | 4 | 18% | | 15 |
| delta(3,5)-Delta(2,4)-dienoyl-CoA isomerase, mitochondrial precursor | 70995211 | 36 | 5 | 32% | 48 | 4 | 28% | | 30 | | 4 | 24% | | 38 |
| ruvB-like 1 | 4506753 | 50 | 5 | 18% | 18 | 4 | 17% | | 15 | | 3 | 10% | | 14 |
| histone H2A type 1-D | 10800130 | 14 | 5 | 35% | 13 | 4 | 30% | | 12 | | 3 | 22% | | 10 |
| N(4)-(beta-N-acetylglucosaminyl)-L-asparaginase isoform 1 preproprotein | 285002251 | 37 | 5 | 22% | 12 | 4 | 20% | | 10 | | 3 | 16% | | 6 |
| vacuolar protein sorting-associated protein 28 homolog isoform 1 | 7705885 | 25 | 5 | 39% | 10 | 4 | 33% | | 7 | | ni | ni | | ni |
| cathepsin Z preproprotein | 22538442 | 34 | 5 | 26% | 13 | 3 | 14% | | 12 | | 5 | 26% | | 15 |
| alpha-centractin | 5031569 | 43 | 5 | 17% | 18 | 3 | 13% | | 15 | | 5 | 17% | | 19 |
| gamma-glutamylcyclotransferase isoform 1 | 13129018 | 21 | 5 | 33% | 11 | 3 | 18% | | 7 | | 2 | 12% | | 3 |
| histone H1.3 | 4885377 | 22 | 5 | 19% | 12 | 3 | 10.00% | | 8 | | 2 | 9.50% | | 6 |
| calpain-1 catalytic subunit isoform X1 | 578821763 | 82 | 5 | 9.20% | 9 | 2 | 5.20% | | 5 | | 5 | 11% | | 10 |
| protein disulfide-isomerase A4 precursor | 4758304 | 73 | 5 | 10% | 19 | 2 | 4.00% | | 6 | | 4 | 7.00% | | 17 |
| importin subunit alpha-1 | 4504897 | 58 | 5 | 27% | 21 | 2 | 11% | | 12 | | 3 | 19% | | 20 |
| endothelial lipase isoform X1 | 530414043 | 61 | 5 | 8.40% | 9 | 2 | 4.90% | | 6 | | 1 | 4.90% | | 1 |
| ras-related protein Rab-2A isoform a | 4506365 | 24 | 5 | 19% | 8 | 1 | 6.60% | | 5 | | 2 | 13% | | 6 |
| ADP/ATP translocase 4 | 13775208 | 35 | 5 | 30% | 18 | 1 | 4.10% | | 1 | | 2 | 13% | | 9 |
| sphingomyelin phosphodiesterase isoform 2 precursor | 300795589 | 70 | 4 | 7.50% | 8 | 9 | 17% | | 21 | | 7 | 13% | | 17 |
| lactoylglutathione lyase | 118402586 | 21 | 4 | 28% | 11 | 9 | 66% | | 24 | | 4 | 29% | | 7 |
| transthyretin precursor | 4507725 | 16 | 4 | 40% | 11 | 8 | 65% | | 26 | | 3 | 28% | | 5 |
| heat shock cognate 71 protein isoform X1 | 578822169 | 71 | 4 | 20% | 15 | 7 | 24% | | 18 | | 7 | 29% | | 18 |
| beta-2-microglobulin precursor | 4757826 | 14 | 4 | 38% | 25 | 7 | 41% | | 42 | | 4 | 38% | | 26 |
| peroxiredoxin-4 precursor | 5453549 | 31 | 4 | 25% | 14 | 7 | 45% | | 25 | | 3 | 24% | | 15 |
| haptoglobin isoform 1 preproprotein | 4826762 | 45 | 4 | 13% | 8 | 7 | 19% | | 16 | | 3 | 7.10% | | 7 |
| soluble calcium-activated nucleotidase 1 | 20270339 | 45 | 4 | 15% | 6 | 6 | 23% | | 14 | | 7 | 30% | | 17 |
| cysteine-rich secretory protein 3 isoform 1 precursor | 300244560 | 29 | 4 | 24% | 18 | 6 | 31% | | 27 | | 5 | 21% | | 21 |
| T-complex protein 1 subunit alpha isoform a | 57863257 | 60 | 4 | 20% | 36 | 5 | 20% | | 34 | | 7 | 20% | | 51 |
| L-lactate dehydrogenase A-like 6B | 15082234 | 42 | 4 | 20% | 32 | 5 | 29% | | 19 | | 6 | 34% | | 41 |
| protein disulfide-isomerase A6 isoform X3 | 530366876 | 49 | 4 | 8.70% | 10 | 5 | 13% | | 12 | | 6 | 16% | | 16 |
| T-complex protein 1 subunit zeta isoform a | 4502643 | 58 | 4 | 10.00% | 19 | 5 | 15% | | 17 | | 6 | 23% | | 21 |
| CD109 antigen isoform 1 precursor | 115529484 | 162 | 4 | 5.20% | 7 | 5 | 6.40% | | 14 | | 6 | 7.30% | | 12 |
| sperm-associated antigen 6 isoform 3 | 359718953 | 52 | 4 | 22% | 31 | 5 | 26% | | 17 | | 5 | 26% | | 31 |
| amyloid beta A4 protein isoform h precursor | 324021738 | 85 | 4 | 9.40% | 10 | 5 | 12% | | 15 | | 5 | 9.40% | | 11 |
| T-complex protein 1 subunit delta isoform b | 375477430 | 55 | 4 | 15% | 17 | 5 | 15% | | 9 | | 4 | 14% | | 15 |
| ubiquitin-like modifier-activating enzyme 1 isoform X1 | 530421539 | 123 | 4 | 4.70% | 12 | 5 | 5.50% | | 12 | | 3 | 5.00% | | 8 |
| pro-cathepsin H preproprotein | 23110955 | 37 | 4 | 19% | 15 | 5 | 19% | | 16 | | 2 | 9.60% | | 6 |
| carbonic anhydrase 2 | 4557395 | 29 | 4 | 21% | 6 | 5 | 25% | | 9 | | 1 | 3.50% | | 1 |
| calreticulin precursor | 4757900 | 48 | 4 | 16% | 19 | 4 | 19% | | 28 | | 9 | 30% | | 35 |
| T-complex protein 1 subunit theta isoform 1 | 48762932 | 60 | 4 | 8.40% | 13 | 4 | 14% | | 15 | | 6 | 11% | | 23 |
| inositol monophosphatase 1 isoform 2 | 221625487 | 37 | 4 | 14% | 11 | 4 | 17% | | 10 | | 5 | 23% | | 12 |
| disintegrin and metalloproteinase domain-containing protein 7 preproprotein | 114326453 | 86 | 4 | 7.60% | 9 | 4 | 8.80% | | 5 | | 5 | 11% | | 10 |
| purine nucleoside phosphorylase | 157168362 | 32 | 4 | 20% | 8 | 4 | 20% | | 11 | | 5 | 24% | | 14 |
| alpha-1-acid glycoprotein 2 precursor | 4505529 | 24 | 4 | 40% | 12 | 4 | 40% | | 19 | | 4 | 27% | | 14 |
| growth/differentiation factor 15 precursor | 153792495 | 34 | 4 | 23% | 18 | 4 | 23% | | 20 | | 4 | 23% | | 10 |
| serine/threonine-protein phosphatase 2A activator isoform b | 29725611 | 37 | 4 | 20% | 10 | 4 | 24% | | 6 | | 4 | 20% | | 8 |
| hydroxyacyl-coenzyme A dehydrogenase, mitochondrial isoform 1 precursor | 296179427 | 36 | 4 | 29% | 23 | 4 | 29% | | 21 | | 3 | 28% | | 33 |
| deoxyribonuclease-2-alpha precursor | 4503349 | 40 | 4 | 11% | 11 | 4 | 19% | | 9 | | 3 | 8.10% | | 10 |
| profilin-1 | 4826898 | 15 | 4 | 40% | 10 | 4 | 40% | | 12 | | 3 | 31% | | 7 |
| heat shock protein beta-1 | 4504517 | 23 | 4 | 28% | 13 | 4 | 31% | | 7 | | 3 | 8.80% | | 6 |
| tripeptidyl-peptidase 2 | 186972143 | 138 | 4 | 8.00% | 9 | 4 | 7.60% | | 11 | | 3 | 6.20% | | 10 |
| succinate-semialdehyde dehydrogenase, mitochondrial isoform 2 precursor | 4507229 | 57 | 4 | 21% | 19 | 4 | 26% | | 10 | | 3 | 21% | | 9 |
| metalloreductase STEAP4 isoform 1 | 100815815 | 52 | 4 | 15% | 8 | 4 | 15% | | 12 | | 3 | 12% | | 7 |
| dolichyl-diphosphooligosaccharide--protein glycosyltransferase subunit 1 precursor | 4506675 | 69 | 4 | 13% | 12 | 4 | 13% | | 10 | | 3 | 9.70% | | 10 |
| ganglioside GM2 activator isoform 1 precursor | 39995109 | 21 | 4 | 23% | 10 | 4 | 31% | | 15 | | 2 | 14% | | 6 |
| protein CutA isoform X1 | 578811722 | 19 | 4 | 41% | 12 | 4 | 41% | | 22 | | 2 | 23% | | 6 |
| protein S100-A9 | 4506773 | 13 | 4 | 44% | 12 | 4 | 45% | | 12 | | 2 | 25% | | 6 |
| sperm acrosome membrane-associated protein 3 | 27777653 | 23 | 4 | 24% | 7 | 4 | 24% | | 11 | | 2 | 12% | | 6 |
| cystatin-SN precursor | 19882251 | 16 | 4 | 57% | 14 | 4 | 56% | | 9 | | 2 | 21% | | 6 |
| hypoxanthine-guanine phosphoribosyltransferase | 4504483 | 25 | 4 | 30% | 10 | 4 | 22% | | 10 | | ni | ni | | ni |
| heat shock 70 protein 13 precursor | 48928056 | 52 | 4 | 8.90% | 6 | 3 | 9.80% | | 7 | | 7 | 18% | | 12 |
| complement factor I isoform X1 | 530377645 | 67 | 4 | 7.40% | 10 | 3 | 6.10% | | 5 | | 5 | 10% | | 10 |
| tubulin alpha-1B chain | 57013276 | 50 | 4 | 39% | 15 | 3 | 43% | | 6 | | 4 | 49% | | 18 |
| interleukin-6 receptor subunit beta isoform 1 precursor | 28610147 | 104 | 4 | 6.10% | 6 | 3 | 5.00% | | 6 | | 4 | 6.10% | | 10 |
| serine protease inhibitor Kazal-type 2 isoform 1 precursor | 413081531 | 14 | 4 | 51% | 33 | 3 | 51% | | 34 | | 3 | 51% | | 25 |
| ribonuclease pancreatic precursor | 38201684 | 18 | 4 | 48% | 31 | 3 | 35% | | 36 | | 3 | 35% | | 17 |
| lysosome-associated membrane glycoprotein 2 isoform C precursor | 169790833 | 45 | 4 | 12% | 16 | 3 | 7.10% | | 14 | | 3 | 7.10% | | 15 |
| histone H3.1 | 10440560 | 15 | 4 | 20% | 9 | 3 | 20% | | 9 | | 3 | 20% | | 11 |
| stress-70 protein, mitochondrial precursor | 24234688 | 74 | 4 | 12% | 9 | 3 | 8.50% | | 8 | | 3 | 8.50% | | 10 |
| calcium-binding mitochondrial carrier protein Aralar2 isoform 1 | 237649019 | 74 | 4 | 9.60% | 15 | 3 | 7.10% | | 5 | | 2 | 3.80% | | 3 |
| N-acetylgalactosamine-6-sulfatase isoform X2 | 530424709 | 59 | 4 | 13% | 8 | 3 | 8.50% | | 5 | | 1 | 4.00% | | 1 |
| programmed cell death protein 6 isoform 2 | 389565483 | 22 | 4 | 32% | 9 | 3 | 26% | | 5 | | ni | ni | | ni |
| histone H2B type 1-D isoform X1 | 530381854 | 16 | 4 | 27% | 14 | 2 | 16% | | 4 | | 6 | 35% | | 14 |
| procollagen-lysine,2-oxoglutarate 5-dioxygenase 2 isoform X1 | 530374784 | 77 | 4 | 10% | 6 | 2 | 3.90% | | 6 | | 4 | 10% | | 9 |
| follistatin-related protein 1 precursor | 5901956 | 35 | 4 | 19% | 12 | 2 | 6.20% | | 9 | | 3 | 10% | | 10 |
| adipocyte plasma membrane-associated protein | 24308201 | 46 | 4 | 21% | 15 | 2 | 8.90% | | 5 | | 2 | 9.90% | | 7 |
| proteasome subunit beta type-5 isoform 1 | 4506201 | 28 | 4 | 19% | 11 | 2 | 9.10% | | 6 | | 1 | 4.90% | | 3 |
| proteasome subunit alpha type-5 isoform 1 | 23110942 | 26 | 4 | 20% | 11 | 2 | 7.90% | | 6 | | ni | ni | | ni |
| proteasome subunit beta type-4 | 22538467 | 29 | 4 | 27% | 9 | 2 | 15% | | 5 | | ni | ni | | ni |
| guanylate cyclase soluble subunit beta-1 | 4504215 | 71 | 4 | 8.70% | 21 | 1 | 1.90% | | 1 | | ni | ni | | ni |
| probable inactive ribonuclease-like protein 13 precursor | 59276062 | 18 | 4 | 22% | 6 | 1 | 10% | | 1 | | ni | ni | | ni |
| leukemia inhibitory factor receptor precursor | 189083786 | 124 | 3 | 4.30% | 8 | 6 | 7.20% | | 16 | | 7 | 7.40% | | 17 |
| cAMP-dependent protein kinase type II-alpha regulatory subunit isoform X1 | 530372834 | 46 | 3 | 12% | 5 | 6 | 25% | | 15 | | 6 | 19% | | 13 |
| puromycin-sensitive aminopeptidase | 158937236 | 103 | 3 | 5.30% | 11 | 6 | 11% | | 12 | | 4 | 5.40% | | 10 |
| annexin A11 isoform 1 | 22165433 | 54 | 3 | 5.30% | 5 | 5 | 13% | | 10 | | 6 | 17% | | 13 |
| endoplasmic reticulum aminopeptidase 1 isoform X1 | 530380009 | 108 | 3 | 4.10% | 7 | 5 | 7.60% | | 13 | | 4 | 5.70% | | 11 |
| kallikrein-11 isoform 2 | 21618357 | 31 | 3 | 14% | 11 | 5 | 18% | | 15 | | 3 | 9.90% | | 6 |
| vitronectin precursor | 88853069 | 54 | 3 | 5.20% | 9 | 5 | 15% | | 14 | | 3 | 8.60% | | 11 |
| metalloproteinase inhibitor 3 precursor | 4507513 | 24 | 3 | 15% | 8 | 5 | 25% | | 14 | | 3 | 14% | | 6 |
| A-kinase anchor protein 4 isoform 1 | 21493037 | 94 | 3 | 6.40% | 25 | 5 | 4.90% | | 18 | | 3 | 6.40% | | 15 |
| glyceraldehyde-3-phosphate dehydrogenase, testis-specific | 7657116 | 45 | 3 | 20% | 19 | 5 | 26% | | 17 | | 2 | 15% | | 13 |
| isoaspartyl peptidase/L-asparaginase | 145275200 | 32 | 3 | 21% | 3 | 5 | 34% | | 8 | | 2 | 16% | | 2 |
| cell division control protein 42 homolog isoform 1 precursor | 4757952 | 21 | 3 | 20% | 9 | 5 | 37% | | 10 | | 2 | 11% | | 2 |
| L-xylulose reductase isoform 2 | 304571975 | 26 | 3 | 19% | 8 | 5 | 35% | | 13 | | 1 | 6.60% | | 1 |
| plasma alpha-L-fucosidase precursor | 40068512 | 54 | 3 | 7.90% | 9 | 4 | 10% | | 11 | | 5 | 12% | | 11 |
| neuroserpin precursor | 170295807 | 46 | 3 | 13% | 10 | 4 | 16% | | 13 | | 5 | 19% | | 18 |
| prenylcysteine oxidase 1 precursor | 166795301 | 57 | 3 | 15% | 19 | 4 | 16% | | 14 | | 5 | 17% | | 18 |
| mitochondrial inner membrane protein isoform 2 | 154354962 | 84 | 3 | 9.50% | 20 | 4 | 12% | | 12 | | 4 | 12% | | 17 |
| glutaminyl-peptide cyclotransferase precursor | 6912618 | 41 | 3 | 23% | 7 | 4 | 19% | | 8 | | 4 | 27% | | 7 |
| leucine-rich repeat-containing protein 15 isoform a precursor | 288541295 | 65 | 3 | 4.30% | 7 | 4 | 5.60% | | 6 | | 4 | 5.60% | | 10 |
| CD59 glycoprotein preproprotein | 10835165 | 14 | 3 | 25% | 9 | 4 | 25% | | 20 | | 3 | 25% | | 14 |
| selenoprotein P isoform 1 precursor | 148277018 | 43 | 3 | 12% | 13 | 4 | 14% | | 19 | | 3 | 12% | | 11 |
| cofilin-1 | 5031635 | 19 | 3 | 34% | 10 | 4 | 44% | | 12 | | 3 | 24% | | 7 |
| izumo sperm-egg fusion protein 4 isoform 3 precursor | 89903023 | 27 | 3 | 29% | 18 | 4 | 34% | | 17 | | 3 | 25% | | 11 |
| serine protease HTRA1 precursor | 4506141 | 51 | 3 | 13% | 9 | 4 | 17% | | 13 | | 3 | 8.50% | | 9 |
| aspartate aminotransferase, mitochondrial isoform 1 precursor | 73486658 | 48 | 3 | 13% | 15 | 4 | 17% | | 8 | | 3 | 13% | | 10 |
| cystatin-M precursor | 4503113 | 17 | 3 | 35% | 11 | 4 | 44% | | 13 | | 2 | 21% | | 6 |
| semaphorin-3F isoform X2 | 530372974 | 88 | 3 | 5.50% | 7 | 4 | 6.20% | | 10 | | 2 | 5.50% | | 6 |
| ras-related protein Rab-3D | 4759000 | 24 | 3 | 28% | 8 | 4 | 44% | | 9 | | 2 | 32% | | 2 |
| gelsolin isoform X7 | 578817383 | 90 | 3 | 5.60% | 5 | 4 | 9.40% | | 9 | | 2 | 3.80% | | 3 |
| peptidyl-prolyl cis-trans isomerase C precursor | 4505991 | 23 | 3 | 15% | 8 | 4 | 31% | | 9 | | 1 | 6.10% | | 3 |
| NAD(P)H-hydrate epimerase precursor | 91984773 | 32 | 3 | 17% | 9 | 4 | 23% | | 11 | | 1 | 4.20% | | 1 |
| D-dopachrome decarboxylase | 145386531 | 13 | 3 | 31% | 9 | 4 | 36% | | 9 | | 1 | 9.30% | | 1 |
| heat shock 70 protein 1-like isoform X1 | 530381921 | 78 | 3 | 26% | 5 | 3 | 27% | | 4 | | 5 | 29% | | 15 |
| leucine-rich repeat-containing protein 37A3 precursor | 75677612 | 181 | 3 | 2.00% | 8 | 3 | 2.00% | | 7 | | 4 | 2.60% | | 12 |
| ubiquitin-40S ribosomal protein S27a precursor | 294459921 | 18 | 3 | 22% | 20 | 3 | 22% | | 22 | | 3 | 22% | | 25 |
| CD9 antigen | 4502693 | 25 | 3 | 21% | 20 | 3 | 21% | | 26 | | 3 | 21% | | 18 |
| collagen alpha-2(VI) chain isoform 2C2 precursor | 115527062 | 109 | 3 | 2.80% | 7 | 3 | 3.00% | | 11 | | 3 | 2.80% | | 11 |
| NADH-ubiquinone oxidoreductase 75 subunit, mitochondrial isoform 5 | 316983160 | 81 | 3 | 6.70% | 34 | 3 | 6.70% | | 25 | | 3 | 6.70% | | 33 |
| cathepsin L1 isoform 1 preproprotein | 384081592 | 38 | 3 | 14% | 11 | 3 | 7.80% | | 7 | | 3 | 12% | | 6 |
| testis-expressed sequence 101 protein isoform 2 precursor | 194097358 | 27 | 3 | 16% | 18 | 3 | 16% | | 20 | | 3 | 16% | | 15 |
| cAMP-dependent protein kinase type I-alpha regulatory subunit isoform a | 47132581 | 43 | 3 | 14% | 29 | 3 | 14% | | 26 | | 3 | 14% | | 25 |
| galactoside 3(4)-L-fucosyltransferase | 148277014 | 42 | 3 | 12% | 5 | 3 | 12% | | 13 | | 3 | 10.00% | | 9 |
| NADH dehydrogenase [ubiquinone] iron-sulfur protein 2, mitochondrial isoform X1 | 530364807 | 53 | 3 | 16% | 31 | 3 | 16% | | 14 | | 3 | 16% | | 24 |
| insulin-like growth factor-binding protein 4 precursor | 62243290 | 28 | 3 | 13% | 8 | 3 | 14% | | 10 | | 3 | 14% | | 9 |
| N-acetylglucosamine-1-phosphotransferase subunit gamma precursor | 14249738 | 34 | 3 | 28% | 10 | 3 | 19% | | 6 | | 3 | 23% | | 6 |
| dehydrogenase/reductase SDR family member 7 isoform X1 | 530403978 | 52 | 3 | 14% | 16 | 3 | 14% | | 18 | | 3 | 14% | | 23 |
| prostate stem cell antigen preproprotein | 289547757 | 12 | 3 | 25% | 17 | 3 | 25% | | 9 | | 3 | 25% | | 20 |
| gamma-glutamyl hydrolase precursor | 4503987 | 36 | 3 | 15% | 8 | 3 | 15% | | 9 | | 3 | 15% | | 6 |
| heat shock protein 75 , mitochondrial isoform 1 precursor | 155722983 | 80 | 3 | 9.70% | 15 | 3 | 9.70% | | 11 | | 3 | 9.70% | | 7 |
| ropporin-1A isoform X1 | 530374814 | 24 | 3 | 20% | 15 | 3 | 20% | | 13 | | 3 | 20% | | 15 |
| proteasome subunit beta type-6 isoform 1 proprotein | 23110925 | 25 | 3 | 25% | 6 | 3 | 25% | | 8 | | 3 | 13% | | 5 |
| dolichyl-diphosphooligosaccharide--protein glycosyltransferase 48 subunit precursor | 20070197 | 51 | 3 | 14% | 16 | 3 | 14% | | 11 | | 3 | 11% | | 9 |
| 2-oxoglutarate dehydrogenase, mitochondrial isoform 3 precursor | 259013553 | 116 | 3 | 7.90% | 8 | 3 | 7.90% | | 5 | | 3 | 7.90% | | 6 |
| neuropilin-1 isoform a precursor | 182508169 | 103 | 3 | 6.10% | 9 | 3 | 6.10% | | 6 | | 2 | 2.90% | | 6 |
| Golgi-associated plant pathogenesis-related protein 1 isoform a | 11641247 | 17 | 3 | 28% | 8 | 3 | 28% | | 9 | | 2 | 17% | | 6 |
| N-acetylglucosamine-6-sulfatase precursor | 4504061 | 62 | 3 | 8.90% | 7 | 3 | 9.60% | | 6 | | 2 | 7.20% | | 5 |
| MAM domain-containing protein 2 precursor | 223278413 | 78 | 3 | 6.30% | 5 | 3 | 7.10% | | 8 | | 2 | 4.10% | | 4 |
| proteasome subunit alpha type-7 | 4506189 | 28 | 3 | 19% | 8 | 3 | 19% | | 9 | | 2 | 10% | | 5 |
| vesicular integral-membrane protein VIP36 precursor | 5803023 | 40 | 3 | 13% | 6 | 3 | 13% | | 12 | | 2 | 13% | | 10 |
| EGF-like repeat and discoidin I-like domain-containing protein 3 isoform 1 precursor | 31317224 | 54 | 3 | 10.00% | 6 | 3 | 10.00% | | 7 | | 2 | 5.00% | | 6 |
| dynein intermediate chain 2, axonemal isoform X4 | 530412670 | 69 | 3 | 12% | 10 | 3 | 9.90% | | 8 | | 2 | 6.60% | | 3 |
| anterior gradient protein 2 homolog isoform X1 | 530384410 | 20 | 3 | 30% | 11 | 3 | 26% | | 10 | | 1 | 10% | | 6 |
| regenerating islet-derived protein 3-gamma isoform 1 precursor | 38348213 | 19 | 3 | 27% | 7 | 3 | 25% | | 11 | | 1 | 10% | | 3 |
| translin isoform 1 | 4759270 | 26 | 3 | 16% | 5 | 3 | 16% | | 10 | | 1 | 9.60% | | 1 |
| proteasome subunit alpha type-4 isoform 1 | 156713442 | 29 | 3 | 21% | 15 | 3 | 15% | | 12 | | 1 | 12% | | 7 |
| zymogen granule protein 16 homolog B precursor | 94536866 | 23 | 3 | 18% | 5 | 3 | 19% | | 12 | | 1 | 4.30% | | 1 |
| thrombospondin-4 precursor | 31543806 | 106 | 3 | 5.30% | 9 | 3 | 6.60% | | 8 | | 1 | 2.60% | | 3 |
| carnitine O-palmitoyltransferase 2, mitochondrial precursor | 4503023 | 74 | 3 | 9.30% | 10 | 3 | 9.30% | | 5 | | 1 | 4.00% | | 2 |
| transforming protein RhoA precursor | 10835049 | 22 | 3 | 23% | 9 | 3 | 26% | | 5 | | 1 | 7.80% | | 2 |
| NME1-NME2 protein | 66392203 | 30 | 3 | 13% | 7 | 3 | 16% | | 7 | | ni | ni | | ni |
| protein FAM3C precursor | 91807125 | 25 | 3 | 20% | 6 | 3 | 19% | | 5 | | ni | ni | | ni |
| CD63 antigen isoform A | 383872455 | 26 | 3 | 7.60% | 11 | 2 | 5.00% | | 12 | | 3 | 7.60% | | 10 |
| selenoprotein S isoform 2 | 33285002 | 21 | 3 | 13% | 18 | 2 | 12% | | 8 | | 3 | 21% | | 9 |
| dystroglycan preproprotein | 294997302 | 98 | 3 | 7.80% | 7 | 2 | 5.50% | | 6 | | 3 | 7.80% | | 7 |
| 3-ketoacyl-CoA thiolase, mitochondrial | 167614485 | 42 | 3 | 20% | 12 | 2 | 14% | | 9 | | 3 | 22% | | 13 |
| NADPH--cytochrome P450 reductase | 127139033 | 77 | 3 | 9.70% | 6 | 2 | 7.20% | | 4 | | 3 | 10.00% | | 6 |
| tetraspanin-1 | 21264578 | 26 | 3 | 11% | 12 | 2 | 7.50% | | 17 | | 2 | 7.50% | | 16 |
| prohibitin isoform 1 | 4505773 | 30 | 3 | 24% | 12 | 2 | 16% | | 7 | | 2 | 16% | | 12 |
| dynactin subunit 1 isoform 1 | 13259510 | 142 | 3 | 5.30% | 5 | 2 | 4.00% | | 6 | | 2 | 4.00% | | 14 |
| 2-hydroxyacyl-CoA lyase 1 isoform d | 548923675 | 55 | 3 | 14% | 6 | 2 | 7.70% | | 7 | | 2 | 7.70% | | 10 |
| 3'(2'),5'-bisphosphate nucleotidase 1 isoform 1 | 116812595 | 33 | 3 | 21% | 13 | 2 | 15% | | 5 | | 2 | 16% | | 3 |
| very long-chain specific acyl-CoA dehydrogenase, mitochondrial isoform 4 | 394025725 | 63 | 3 | 10% | 8 | 2 | 5.90% | | 4 | | 2 | 5.90% | | 5 |
| arrestin domain-containing protein 1 | 22748653 | 46 | 3 | 18% | 5 | 2 | 10% | | 6 | | 2 | 10% | | 5 |
| AFG3-like protein 2 | 300192933 | 89 | 3 | 9.70% | 5 | 2 | 7.20% | | 6 | | 2 | 7.20% | | 3 |
| myosin-9 | 12667788 | 227 | 3 | 1.10% | 3 | 2 | 0.71% | | 3 | | 1 | 0.71% | | 3 |
| T-complex protein 1 subunit zeta-2 isoform 2 | 302058290 | 54 | 3 | 20% | 12 | 2 | 15% | | 9 | | 1 | 12% | | 10 |
| ras-related protein Rap-1b isoform 4 | 354459356 | 15 | 3 | 18% | 5 | 2 | 17% | | 6 | | 1 | 8.00% | | 3 |
| medium-chain specific acyl-CoA dehydrogenase, mitochondrial isoform b precursor | 187960098 | 47 | 3 | 14% | 14 | 2 | 9.90% | | 7 | | 1 | 4.20% | | 5 |
| histone H2B type 1-A | 24586679 | 14 | 3 | 37% | 8 | 1 | 19% | | 1 | | 3 | 41% | | 6 |
| G-protein coupled receptor family C group 5 member C isoform X1 | 530412533 | 60 | 3 | 6.00% | 5 | 1 | 1.60% | | 2 | | 3 | 6.70% | | 8 |
| protein NOV homolog precursor | 4505423 | 39 | 3 | 14% | 9 | 1 | 4.20% | | 3 | | 2 | 8.10% | | 7 |
| kunitz-type protease inhibitor 3 precursor | 189571689 | 10 | 3 | 29% | 10 | 1 | 19% | | 8 | | 1 | 19% | | 3 |
| carnitine O-acetyltransferase isoform 2 | 383209673 | 69 | 3 | 15% | 8 | 1 | 5.80% | | 2 | | 1 | 5.80% | | 6 |
| protein ERGIC-53 precursor | 5031873 | 58 | 3 | 8.40% | 7 | 1 | 2.50% | | 2 | | 1 | 2.50% | | 3 |
| ribonuclease inhibitor | 42822868 | 50 | 3 | 7.80% | 3 | ni | ni | | ni | | 3 | 8.00% | | 6 |
| reticulocalbin-2 isoform b precursor | 426214088 | 39 | 3 | 17% | 6 | ni | ni | | ni | | ni | ni | | ni |
| selenium-binding protein 1 isoform 3 | 385137130 | 57 | 2 | 6.80% | 6 | 7 | 19% | | 14 | | 6 | 15% | | 14 |
| apolipoprotein A-I preproprotein | 4557321 | 31 | 2 | 9.00% | 6 | 6 | 22% | | 18 | | 3 | 13% | | 10 |
| N-acetyllactosaminide beta-1,3-N-acetylglucosaminyltransferase | 5802984 | 47 | 2 | 9.90% | 6 | 5 | 24% | | 11 | | 4 | 19% | | 7 |
| isoleucine--tRNA ligase, mitochondrial precursor | 46852147 | 114 | 2 | 3.80% | 9 | 4 | 9.10% | | 12 | | 4 | 9.90% | | 16 |
| antithrombin-III isoform X1 | 530364787 | 47 | 2 | 12% | 2 | 4 | 19% | | 5 | | 3 | 12% | | 5 |
| adenylyl cyclase-associated protein 1 isoform X1 | 530361588 | 52 | 2 | 7.60% | 10 | 4 | 7.60% | | 17 | | 3 | 7.60% | | 8 |
| 45 calcium-binding protein isoform 2 precursor | 18699732 | 42 | 2 | 5.80% | 3 | 4 | 17% | | 8 | | 3 | 14% | | 9 |
| fructose-1,6-bisphosphatase 1 | 16579888 | 37 | 2 | 9.80% | 6 | 4 | 20% | | 9 | | 3 | 14% | | 7 |
| ras-related protein Rab-27B isoform X1 | 530414276 | 25 | 2 | 17% | 5 | 4 | 28% | | 8 | | 2 | 15% | | 4 |
| lysosomal acid lipase/cholesteryl ester hydrolase isoform 1 precursor | 189083851 | 45 | 2 | 12% | 5 | 4 | 20% | | 9 | | 2 | 12% | | 3 |
| peptidase inhibitor 15 preproprotein | 7705676 | 29 | 2 | 19% | 6 | 4 | 26% | | 9 | | 1 | 11% | | 2 |
| proteasome subunit beta type-2 isoform 2 | 315139006 | 20 | 2 | 15% | 5 | 4 | 43% | | 9 | | 1 | 6.20% | | 3 |
| 14-3-3 protein theta | 5803227 | 28 | 2 | 18% | 4 | 4 | 23% | | 8 | | 1 | 15% | | 1 |
| ADP-ribosylation factor 1 | 4502201 | 21 | 2 | 18% | 5 | 4 | 33% | | 10 | | ni | ni | | ni |
| cytochrome b-c1 complex subunit 2, mitochondrial precursor | 50592988 | 48 | 2 | 13% | 47 | 3 | 22% | | 29 | | 3 | 22% | | 34 |
| bile salt-activated lipase precursor | 148536848 | 80 | 2 | 3.70% | 5 | 3 | 6.70% | | 7 | | 3 | 6.70% | | 8 |
| fibronectin isoform 1 preproprotein | 47132557 | 272 | 2 | 54% | 7 | 3 | 64% | | 19 | | 3 | 59% | | 22 |
| protein lifeguard 3 isoform X1 | 530370762 | 35 | 2 | 4.80% | 7 | 3 | 4.80% | | 9 | | 3 | 4.80% | | 14 |
| ATP synthase subunit gamma, mitochondrial isoform L (liver) precursor | 50345988 | 33 | 2 | 15% | 25 | 3 | 19% | | 17 | | 3 | 19% | | 18 |
| prostate and testis expressed protein 4 precursor | 221554530 | 11 | 2 | 23% | 6 | 3 | 31% | | 10 | | 3 | 30% | | 6 |
| acid sphingomyelinase-like phosphodiesterase 3a isoform a precursor | 24307911 | 51 | 2 | 8.60% | 6 | 3 | 10% | | 7 | | 3 | 12% | | 7 |
| protein S100-A8 | 21614544 | 11 | 2 | 24% | 4 | 3 | 31% | | 7 | | 3 | 31% | | 8 |
| proteasome subunit beta type-3 | 22538465 | 23 | 2 | 16% | 12 | 3 | 19% | | 9 | | 3 | 23% | | 5 |
| apoptosis-inducing factor 1, mitochondrial isoform 1 precursor | 4757732 | 67 | 2 | 6.40% | 11 | 3 | 9.60% | | 11 | | 3 | 9.60% | | 17 |
| thioredoxin reductase 2, mitochondrial isoform 1 precursor | 22035672 | 57 | 2 | 7.60% | 6 | 3 | 13% | | 6 | | 3 | 13% | | 9 |
| acetyl-CoA acetyltransferase, cytosolic | 148539872 | 41 | 2 | 6.80% | 3 | 3 | 19% | | 3 | | 3 | 21% | | 4 |
| uncharacterized protein LOC100293211 | 578798051 | 29 | 2 | 11% | 5 | 3 | 15% | | 7 | | 2 | 8.10% | | 8 |
| 6-phosphofructokinase type C isoform X2 | 530392191 | 86 | 2 | 5.00% | 6 | 3 | 4.60% | | 4 | | 2 | 4.80% | | 2 |
| NADH dehydrogenase [ubiquinone] flavoprotein 1, mitochondrial isoform 1 precursor | 20149568 | 51 | 2 | 9.70% | 9 | 3 | 14% | | 8 | | 2 | 11% | | 12 |
| lipocalin-15 isoform X1 | 578817458 | 16 | 2 | 14% | 6 | 3 | 25% | | 6 | | 2 | 14% | | 4 |
| pyruvate dehydrogenase E1 component subunit beta, mitochondrial isoform 1 precursor | 156564403 | 39 | 2 | 11% | 7 | 3 | 19% | | 8 | | 2 | 11% | | 5 |
| platelet-activating factor acetylhydrolase isoform X1 | 530382617 | 50 | 2 | 9.80% | 2 | 3 | 15% | | 5 | | 2 | 8.40% | | 4 |
| acrosomal protein SP-10 isoform a precursor | 4501879 | 28 | 2 | 15% | 3 | 3 | 18% | | 5 | | 1 | 8.70% | | 3 |
| nephronectin isoform D precursor | 296011071 | 59 | 2 | 7.60% | 3 | 3 | 10% | | 6 | | 1 | 2.80% | | 1 |
| glutathione peroxidase 3 precursor | 6006001 | 26 | 2 | 14% | 6 | 3 | 19% | | 8 | | 1 | 7.50% | | 2 |
| C3 and PZP-like alpha-2-macroglobulin domain-containing protein 8 | 118600977 | 211 | 2 | 2.30% | 3 | 3 | 5.00% | | 5 | | 1 | 1.60% | | 4 |
| mesencephalic astrocyte-derived neurotrophic factor precursor | 299523086 | 21 | 2 | 15% | 2 | 3 | 15% | | 3 | | ni | ni | | ni |
| putative phospholipase B-like 2 isoform 1 precursor | 229093316 | 65 | 2 | 4.20% | 4 | 2 | 5.10% | | 4 | | 4 | 12% | | 5 |
| glutathione synthetase isoform X2 | 530418095 | 52 | 2 | 5.90% | 3 | 2 | 4.20% | | 10 | | 3 | 10% | | 10 |
| carbonic anhydrase 4 precursor | 4502519 | 35 | 2 | 9.00% | 7 | 2 | 9.00% | | 10 | | 3 | 6.40% | | 5 |
| calnexin precursor | 66933005 | 68 | 2 | 9.50% | 6 | 2 | 12% | | 10 | | 3 | 15% | | 9 |
| disintegrin and metalloproteinase domain-containing protein 10 precursor | 4557251 | 84 | 2 | 2.00% | 4 | 2 | 2.90% | | 3 | | 3 | 4.90% | | 8 |
| alpha-actinin-1 isoform a | 194097350 | 106 | 2 | 13% | 6 | 2 | 12% | | 3 | | 3 | 15% | | 3 |
| twisted gastrulation protein homolog 1 precursor | 10190664 | 25 | 2 | 16% | 7 | 2 | 16% | | 14 | | 2 | 16% | | 9 |
| complement C1q tumor necrosis factor-related protein 1 isoform X1 | 578830330 | 42 | 2 | 9.80% | 4 | 2 | 6.10% | | 6 | | 2 | 9.80% | | 4 |
| short-chain specific acyl-CoA dehydrogenase, mitochondrial precursor | 4557233 | 44 | 2 | 10% | 20 | 2 | 10% | | 14 | | 2 | 11% | | 13 |
| superoxide dismutase [Mn], mitochondrial isoform A precursor | 67782305 | 25 | 2 | 26% | 12 | 2 | 26% | | 7 | | 2 | 26% | | 12 |
| sperm acrosome membrane-associated protein 4 precursor | 19424138 | 13 | 2 | 22% | 9 | 2 | 22% | | 9 | | 2 | 22% | | 4 |
| phosphoglucomutase-1 isoform 1 | 21361621 | 61 | 2 | 9.80% | 3 | 2 | 9.80% | | 5 | | 2 | 9.80% | | 3 |
| complement component C9 precursor | 4502511 | 63 | 2 | 6.80% | 5 | 2 | 6.80% | | 4 | | 2 | 6.80% | | 5 |
| vasorin precursor | 88702793 | 72 | 2 | 6.50% | 4 | 2 | 6.50% | | 5 | | 2 | 6.50% | | 5 |
| electron transfer flavoprotein subunit alpha, mitochondrial isoform b | 189181759 | 30 | 2 | 9.20% | 2 | 2 | 15% | | 3 | | 2 | 9.20% | | 2 |
| actin-related protein T2 | 29893808 | 42 | 2 | 12% | 8 | 2 | 12% | | 4 | | 2 | 12% | | 7 |
| isochorismatase domain-containing protein 2, mitochondrial isoform 1 | 209969695 | 22 | 2 | 24% | 4 | 2 | 24% | | 8 | | 2 | 24% | | 6 |
| 2-oxoisovalerate dehydrogenase subunit beta, mitochondrial precursor | 34101272 | 43 | 2 | 19% | 5 | 2 | 19% | | 5 | | 2 | 19% | | 6 |
| phosphoglycolate phosphatase | 108796653 | 34 | 2 | 23% | 5 | 2 | 23% | | 5 | | 2 | 23% | | 3 |
| methylmalonyl-CoA mutase, mitochondrial isoform X1 | 530382073 | 83 | 2 | 8.70% | 5 | 2 | 7.20% | | 4 | | 2 | 8.70% | | 4 |
| V-type proton ATPase catalytic subunit A | 19913424 | 68 | 2 | 11% | 3 | 2 | 8.60% | | 2 | | 2 | 11% | | 4 |
| aspartyl aminopeptidase | 156416028 | 53 | 2 | 12% | 2 | 2 | 12% | | 2 | | 2 | 12% | | 2 |
| insulin-like growth factor-binding protein 5 precursor | 10834982 | 31 | 2 | 11% | 12 | 2 | 11% | | 4 | | 1 | 5.90% | | 2 |
| cytochrome c | 11128019 | 12 | 2 | 24% | 4 | 2 | 18% | | 5 | | 1 | 7.60% | | 1 |
| amyloid-like protein 2 isoform 2 precursor | 214010181 | 85 | 2 | 3.20% | 2 | 2 | 4.10% | | 2 | | 1 | 1.10% | | 1 |
| hsc70-interacting protein isoform 1 | 19923193 | 41 | 2 | 6.50% | 4 | 2 | 7.30% | | 5 | | 1 | 3.80% | | 3 |
| protein S100-A11 | 5032057 | 12 | 2 | 24% | 6 | 2 | 24% | | 5 | | 1 | 8.60% | | 1 |
| thioredoxin isoform 1 | 50592994 | 12 | 2 | 10% | 5 | 2 | 23% | | 4 | | 1 | 12% | | 1 |
| cysteine-rich secretory protein 2 isoform X10 | 530382514 | 27 | 2 | 21% | 11 | 2 | 21% | | 10 | | 1 | 14% | | 5 |
| lysozyme-like protein 4 precursor | 21389465 | 16 | 2 | 15% | 7 | 2 | 15% | | 4 | | 1 | 6.80% | | 1 |
| thioredoxin domain-containing protein 3 | 148839372 | 67 | 2 | 6.00% | 4 | 2 | 3.60% | | 5 | | 1 | 3.60% | | 2 |
| V-type proton ATPase subunit B, brain isoform | 19913428 | 57 | 2 | 6.80% | 13 | 2 | 6.80% | | 6 | | 1 | 4.10% | | 7 |
| junctional adhesion molecule A precursor | 8393638 | 33 | 2 | 7.70% | 4 | 2 | 7.70% | | 6 | | 1 | 4.00% | | 2 |
| dihydrolipoyllysine-residue acetyltransferase component of pyruvate dehydrogenase complex, mitochondrial precursor | 31711992 | 69 | 2 | 5.70% | 6 | 2 | 5.70% | | 6 | | 1 | 2.30% | | 5 |
| mammalian ependymin-related protein 1 isoform 1 precursor | 345110632 | 25 | 2 | 8.90% | 2 | 2 | 8.90% | | 5 | | 1 | 4.90% | | 1 |
| acid sphingomyelinase-like phosphodiesterase 3b isoform 1 precursor | 57242798 | 51 | 2 | 6.20% | 4 | 2 | 7.70% | | 5 | | 1 | 2.60% | | 3 |
| N(G),N(G)-dimethylarginine dimethylaminohydrolase 1 isoform 1 | 6912328 | 31 | 2 | 18% | 2 | 2 | 13% | | 5 | | 1 | 9.10% | | 2 |
| collectin-12 | 18641360 | 82 | 2 | 3.50% | 4 | 2 | 3.50% | | 4 | | 1 | 2.20% | | 3 |
| dynein light chain 2, cytoplasmic | 18087855 | 10 | 2 | 20% | 6 | 2 | 26% | | 5 | | ni | ni | | ni |
| plasma membrane calcium-transporting ATPase 4 isoform 4b | 48255957 | 134 | 2 | 4.20% | 8 | 2 | 2.50% | | 3 | | ni | ni | | ni |
| proteasome subunit alpha type-3 isoform 2 | 23110939 | 28 | 2 | 8.90% | 6 | 2 | 8.90% | | 6 | | ni | ni | | ni |
| prostate and testis expressed protein 2 precursor | 47086459 | 13 | 2 | 27% | 4 | 2 | 27% | | 5 | | ni | ni | | ni |
| nucleoside diphosphate kinase 3 precursor | 37693993 | 19 | 2 | 20% | 4 | 2 | 20% | | 5 | | ni | ni | | ni |
| semaphorin-3C precursor | 5454048 | 85 | 2 | 6.10% | 3 | 2 | 6.10% | | 4 | | ni | ni | | ni |
| cysteine desulfurase, mitochondrial isoform b precursor | 312222769 | 44 | 2 | 12% | 5 | 2 | 9.40% | | 2 | | ni | ni | | ni |
| pigment epithelium-derived factor precursor | 39725934 | 46 | 2 | 12% | 4 | 1 | 7.20% | | 3 | | 4 | 17% | | 8 |
| lipase member I isoform X1 | 578836443 | 56 | 2 | 4.50% | 5 | 1 | 2.00% | | 3 | | 3 | 6.70% | | 5 |
| UTP--glucose-1-phosphate uridylyltransferase isoform a | 48255966 | 57 | 2 | 11% | 5 | 1 | 3.90% | | 3 | | 2 | 9.80% | | 4 |
| cytochrome b-c1 complex subunit Rieske, mitochondrial | 163644321 | 30 | 2 | 15% | 22 | 1 | 7.70% | | 1 | | 2 | 7.70% | | 4 |
| epoxide hydrolase 1 | 209862837 | 53 | 2 | 11% | 11 | 1 | 5.10% | | 3 | | 2 | 11% | | 11 |
| isovaleryl-CoA dehydrogenase, mitochondrial isoform 1 precursor | 226958412 | 47 | 2 | 9.20% | 7 | 1 | 5.20% | | 3 | | 2 | 9.20% | | 8 |
| dual specificity protein phosphatase 14 isoform X1 | 530411418 | 22 | 2 | 14% | 4 | 1 | 6.10% | | 3 | | 2 | 14% | | 3 |
| glyoxalase domain-containing protein 4 isoform X1 | 578829940 | 32 | 2 | 8.30% | 5 | 1 | 4.50% | | 1 | | 2 | 13% | | 4 |
| acyl-CoA dehydrogenase family member 9, mitochondrial | 21361497 | 69 | 2 | 6.80% | 2 | 1 | 2.10% | | 1 | | 2 | 10% | | 2 |
| macrophage migration inhibitory factor | 4505185 | 12 | 2 | 28% | 6 | 1 | 9.60% | | 8 | | 1 | 9.60% | | 6 |
| CD81 antigen | 4757944 | 26 | 2 | 15% | 10 | 1 | 9.70% | | 2 | | 1 | 9.70% | | 2 |
| HD domain-containing protein 2 | 116875826 | 23 | 2 | 17% | 5 | 1 | 8.30% | | 3 | | 1 | 8.30% | | 3 |
| chloride intracellular channel protein 1 | 14251209 | 27 | 2 | 12% | 5 | 1 | 7.50% | | 5 | | 1 | 7.50% | | 3 |
| transmembrane emp24 domain-containing protein 4 precursor | 33457308 | 26 | 2 | 8.80% | 5 | 1 | 4.00% | | 3 | | 1 | 4.00% | | 2 |
| plakophilin-1 isoform 1a | 53729344 | 80 | 2 | 3.60% | 3 | 1 | 1.50% | | 3 | | 1 | 1.50% | | 3 |
| nidogen-1 precursor | 115298674 | 136 | 2 | 3.40% | 3 | 1 | 2.60% | | 3 | | 1 | 2.60% | | 3 |
| 3-hydroxyacyl-CoA dehydrogenase type-2 isoform 1 | 4758504 | 27 | 2 | 17% | 7 | 1 | 9.60% | | 3 | | 1 | 9.60% | | 3 |
| endoplasmic reticulum resident protein 44 precursor | 52487191 | 47 | 2 | 9.60% | 3 | 1 | 9.60% | | 1 | | 1 | 5.70% | | 1 |
| 2',3'-cyclic-nucleotide 3'-phosphodiesterase isoform X1 | 578830418 | 45 | 2 | 12% | 3 | 1 | 6.20% | | 3 | | 1 | 6.20% | | 3 |
| mammaglobin-B precursor | 4505171 | 11 | 2 | 32% | 5 | 1 | 13% | | 3 | | 1 | 13% | | 2 |
| uncharacterized protein KIAA2013 precursor | 25286703 | 69 | 2 | 7.90% | 3 | 1 | 3.60% | | 3 | | 1 | 3.60% | | 3 |
| nicastrin isoform 1 precursor | 24638433 | 78 | 2 | 6.30% | 6 | 1 | 3.20% | | 1 | | 1 | 3.10% | | 2 |
| calcium-binding mitochondrial carrier protein Aralar1 | 21361103 | 75 | 2 | 9.60% | 4 | 1 | 3.20% | | 3 | | 1 | 3.20% | | 3 |
| membrane metallo-endopeptidase-like 1 | 239049391 | 89 | 2 | 3.00% | 3 | 1 | 3.00% | | 1 | | 1 | 3.00% | | 3 |
| interferon-induced transmembrane protein 1 | 150010589 | 14 | 2 | 27% | 4 | 1 | 13% | | 3 | | ni | ni | | ni |
| immunoglobulin J chain precursor | 21489959 | 18 | 2 | 17% | 4 | 1 | 7.50% | | 3 | | ni | ni | | ni |
| thrombospondin-1 precursor | 40317626 | 129 | 2 | 3.00% | 4 | 1 | 2.00% | | 3 | | ni | ni | | ni |
| retinol-binding protein 4 precursor | 55743122 | 23 | 2 | 16% | 2 | 1 | 5.00% | | 1 | | ni | ni | | ni |
| ras-related protein Rab-14 | 19923483 | 24 | 2 | 21% | 2 | 1 | 12% | | 3 | | ni | ni | | ni |
| transmembrane emp24 domain-containing protein 10 precursor | 98986464 | 25 | 2 | 12% | 5 | ni | ni | | ni | | 2 | 9.60% | | 3 |
| bifunctional glutamate/proline--tRNA ligase | 62241042 | 171 | 2 | 2.20% | 4 | ni | ni | | ni | | 2 | 1.70% | | 2 |
| epididymis-specific alpha-mannosidase precursor | 50659093 | 114 | 2 | 2.80% | 4 | ni | ni | | ni | | 1 | 1.40% | | 2 |
| tetranectin precursor | 156627579 | 23 | 2 | 12% | 2 | ni | ni | | ni | | ni | ni | | ni |
| thioredoxin domain-containing protein 16 isoform 1 precursor | 237648959 | 94 | 1 | 2.90% | 1 | 6 | 11% | | 13 | | 1 | 1.50% | | 3 |
| alpha-N-acetylgalactosaminidase isoform X1 | 530420112 | 47 | 1 | 2.90% | 3 | 5 | 17% | | 13 | | 5 | 19% | | 7 |
| phosphoglycerate mutase 1 | 4505753 | 29 | 1 | 13% | 2 | 5 | 41% | | 13 | | 2 | 22% | | 2 |
| brain-specific serine protease 4 isoform X1 | 530408666 | 34 | 1 | 7.60% | 3 | 4 | 20% | | 9 | | 3 | 16% | | 5 |
| glutathione S-transferase Mu 4 isoform 2 | 23065557 | 23 | 1 | 6.70% | 3 | 4 | 29% | | 10 | | 2 | 15% | | 4 |
| angiogenin precursor | 4557313 | 17 | 1 | 6.10% | 1 | 4 | 37% | | 15 | | 1 | 14% | | 3 |
| CDGSH iron-sulfur domain-containing protein 1 | 8923930 | 12 | 1 | 21% | 2 | 3 | 30% | | 15 | | 3 | 30% | | 10 |
| granulins isoform X1 | 530411988 | 64 | 1 | 2.40% | 3 | 3 | 4.90% | | 7 | | 2 | 4.40% | | 4 |
| alcohol dehydrogenase [NADP(+)] | 320202986 | 37 | 1 | 3.10% | 3 | 3 | 12% | | 4 | | 2 | 5.50% | | 4 |
| glypican-1 precursor | 167001141 | 62 | 1 | 2.50% | 3 | 3 | 8.80% | | 6 | | 2 | 5.40% | | 6 |
| EGF-containing fibulin-like extracellular matrix protein 1 precursor | 86788132 | 55 | 1 | 4.70% | 4 | 3 | 7.50% | | 14 | | 1 | 4.70% | | 5 |
| contactin-associated protein-like 2 precursor | 7662350 | 148 | 1 | 0.83% | 1 | 3 | 3.40% | | 7 | | 1 | 0.83% | | 3 |
| beta-galactoside alpha-2,6-sialyltransferase 1 isoform X5 | 578807681 | 47 | 1 | 3.90% | 2 | 3 | 9.40% | | 6 | | 1 | 3.90% | | 2 |
| transketolase isoform 1 | 205277463 | 68 | 1 | 3.50% | 2 | 3 | 9.50% | | 5 | | 1 | 3.00% | | 3 |
| ras-related protein Rab-11A isoform 1 | 4758984 | 24 | 1 | 5.10% | 2 | 3 | 13% | | 5 | | 1 | 13% | | 1 |
| receptor-type tyrosine-protein phosphatase F isoform X12 | 578799535 | 193 | 1 | 0.87% | 1 | 3 | 2.20% | | 6 | | ni | ni | | ni |
| N-sulphoglucosamine sulphohydrolase isoform X2 | 530412680 | 37 | 1 | 7.90% | 2 | 2 | 11% | | 5 | | 3 | 15% | | 5 |
| pancreatic secretory granule membrane major glycoprotein GP2 isoform X2 | 530407931 | 73 | 1 | 1.80% | 3 | 2 | 4.10% | | 5 | | 3 | 5.60% | | 7 |
| iduronate 2-sulfatase isoform c | 262118210 | 52 | 1 | 4.10% | 1 | 2 | 8.30% | | 6 | | 3 | 10% | | 6 |
| huntingtin-interacting protein 1 isoform 1 | 38045919 | 116 | 1 | 2.90% | 3 | 2 | 2.90% | | 3 | | 3 | 4.80% | | 5 |
| mesothelin isoform 1 preproprotein | 293651530 | 68 | 1 | 2.30% | 3 | 2 | 6.30% | | 5 | | 2 | 4.70% | | 6 |
| beta-glucuronidase isoform 1 precursor | 268834192 | 75 | 1 | 3.10% | 2 | 2 | 7.40% | | 4 | | 2 | 4.50% | | 4 |
| choline transporter-like protein 4 isoform 1 | 148612887 | 79 | 1 | 2.00% | 7 | 2 | 3.10% | | 10 | | 2 | 3.10% | | 12 |
| collagen alpha-1(VI) chain precursor | 87196339 | 109 | 1 | 1.80% | 2 | 2 | 4.00% | | 8 | | 2 | 4.00% | | 6 |
| peroxiredoxin-5, mitochondrial isoform a precursor | 6912238 | 22 | 1 | 7.90% | 3 | 2 | 16% | | 4 | | 2 | 7.90% | | 2 |
| plastin-3 isoform 2 | 288915539 | 68 | 1 | 13% | 1 | 2 | 19% | | 4 | | 2 | 18% | | 4 |
| ester hydrolase C11orf54 isoform c | 554506535 | 33 | 1 | 5.70% | 1 | 2 | 9.80% | | 6 | | 2 | 11% | | 4 |
| EGF-containing fibulin-like extracellular matrix protein 2 precursor | 320118911 | 49 | 1 | 2.70% | 3 | 2 | 6.10% | | 4 | | 2 | 6.10% | | 4 |
| xaa-Pro dipeptidase isoform 1 | 149589008 | 55 | 1 | 5.70% | 3 | 2 | 7.70% | | 4 | | 2 | 7.70% | | 5 |
| 40S ribosomal protein SA | 59859885 | 33 | 1 | 2.70% | 1 | 2 | 7.10% | | 4 | | 2 | 8.10% | | 2 |
| lipocalin-1 isoform 1 precursor | 357933617 | 19 | 1 | 6.20% | 3 | 2 | 13% | | 5 | | 1 | 6.20% | | 2 |
| lysozyme C precursor | 4557894 | 17 | 1 | 19% | 3 | 2 | 27% | | 6 | | 1 | 8.10% | | 3 |
| Golgi membrane protein 1 | 29550838 | 45 | 1 | 2.50% | 3 | 2 | 6.00% | | 6 | | 1 | 2.50% | | 3 |
| solute carrier family 2, facilitated glucose transporter member 3 | 5902090 | 54 | 1 | 1.40% | 1 | 2 | 2.60% | | 2 | | 1 | 1.20% | | 1 |
| ras-related C3 botulinum toxin substrate 1 isoform Rac1b | 9845509 | 23 | 1 | 12% | 1 | 2 | 12% | | 5 | | 1 | 4.70% | | 3 |
| lipid phosphate phosphohydrolase 1 isoform 2 | 29171738 | 32 | 1 | 7.00% | 2 | 2 | 10% | | 9 | | 1 | 7.00% | | 5 |
| torsin-1B precursor | 14149653 | 38 | 1 | 4.50% | 1 | 2 | 8.30% | | 3 | | 1 | 4.50% | | 3 |
| calmodulin | 4502549 | 17 | 1 | 11% | 3 | 2 | 22% | | 5 | | 1 | 11% | | 1 |
| ovochymase-2 precursor | 373838920 | 63 | 1 | 3.00% | 2 | 2 | 5.00% | | 6 | | 1 | 3.00% | | 1 |
| homogentisate 1,2-dioxygenase isoform X2 | 530374530 | 38 | 1 | 7.10% | 1 | 2 | 11% | | 2 | | 1 | 7.10% | | 2 |
| protein-L-isoaspartate(D-aspartate) O-methyltransferase isoform 1 | 226530908 | 30 | 1 | 6.00% | 3 | 2 | 18% | | 3 | | ni | ni | | ni |
| glutathione S-transferase theta-1 | 167466164 | 27 | 1 | 7.90% | 1 | 2 | 12% | | 3 | | ni | ni | | ni |
| out at first protein homolog precursor | 30425438 | 31 | 1 | 6.20% | 1 | 2 | 10% | | 3 | | ni | ni | | ni |
| V-set and transmembrane domain-containing protein 2-like protein precursor | 18079321 | 22 | 1 | 5.90% | 2 | 2 | 18% | | 2 | | ni | ni | | ni |
| protein DPCD | 39930355 | 23 | 1 | 4.90% | 1 | 2 | 10% | | 3 | | ni | ni | | ni |
| ras-related protein Rab-7a | 34147513 | 23 | 1 | 6.30% | 1 | 2 | 13% | | 5 | | ni | ni | | ni |
| tumor necrosis factor receptor superfamily member 19 isoform X2 | 530402486 | 36 | 1 | 4.90% | 2 | 2 | 7.60% | | 8 | | ni | ni | | ni |
| calcium and integrin-binding protein 1 isoform b | 163644313 | 22 | 1 | 7.90% | 3 | 2 | 15% | | 4 | | ni | ni | | ni |
| lysozyme-like protein 6 precursor | 317008577 | 17 | 1 | 6.10% | 1 | 2 | 24% | | 2 | | ni | ni | | ni |
| deoxyribonuclease-1 isoform X7 | 578827998 | 31 | 1 | 13% | 1 | 2 | 18% | | 2 | | ni | ni | | ni |
| protein phosphatase 1 regulatory subunit 7 isoform 1 | 4506013 | 42 | 1 | 5.00% | 3 | 1 | 5.00% | | 1 | | 3 | 10% | | 7 |
| CD44 antigen isoform 1 precursor | 48255935 | 82 | 1 | 1.30% | 3 | 1 | 1.60% | | 2 | | 2 | 3.00% | | 4 |
| seizure 6-like protein 2 isoform 2 precursor | 166235136 | 98 | 1 | 2.30% | 3 | 1 | 0.88% | | 2 | | 2 | 3.20% | | 3 |
| proteasome subunit beta type-7 proprotein | 4506203 | 30 | 1 | 10% | 6 | 1 | 10% | | 5 | | 2 | 14% | | 12 |
| desmoglein-4 isoform 1 preproprotein | 197313787 | 115 | 1 | 0.76% | 3 | 1 | 0.76% | | 3 | | 2 | 1.80% | | 5 |
| F-actin-capping protein subunit alpha-1 | 5453597 | 33 | 1 | 3.50% | 2 | 1 | 3.50% | | 2 | | 2 | 8.70% | | 5 |
| synaptic vesicle membrane protein VAT-1 homolog | 18379349 | 42 | 1 | 5.90% | 3 | 1 | 4.60% | | 2 | | 2 | 8.10% | | 3 |
| NADP-dependent malic enzyme | 4505143 | 64 | 1 | 4.50% | 3 | 1 | 4.50% | | 3 | | 2 | 9.80% | | 4 |
| single-stranded DNA-binding protein, mitochondrial precursor | 374671775 | 17 | 1 | 12% | 4 | 1 | 10% | | 1 | | 2 | 22% | | 4 |
| protein CREG1 precursor | 4503037 | 24 | 1 | 9.50% | 3 | 1 | 9.50% | | 6 | | 1 | 9.50% | | 3 |
| L-seryl-tRNA(Sec) kinase isoform X1 | 530392997 | 41 | 1 | 3.40% | 4 | 1 | 3.40% | | 2 | | 1 | 3.40% | | 2 |
| heat shock 70 protein 4L | 31541941 | 95 | 1 | 2.50% | 3 | 1 | 2.00% | | 3 | | 1 | 2.50% | | 1 |
| 4F2 cell-surface antigen heavy chain isoform f | 61744483 | 58 | 1 | 2.30% | 3 | 1 | 2.30% | | 3 | | 1 | 2.30% | | 3 |
| beta-galactosidase-1-like protein isoform X1 | 530370954 | 74 | 1 | 3.10% | 1 | 1 | 3.10% | | 1 | | 1 | 4.90% | | 1 |
| N-acetylated-alpha-linked acidic dipeptidase 2 | 4885505 | 84 | 1 | 1.50% | 1 | 1 | 1.50% | | 3 | | ni | ni | | ni |
| spermine synthase isoform 2 | 386643030 | 35 | 1 | 13% | 1 | 1 | 8.90% | | 3 | | ni | ni | | ni |
| GTP-binding nuclear protein Ran | 5453555 | 24 | 1 | 4.60% | 1 | 1 | 6.50% | | 3 | | ni | ni | | ni |
| tumor necrosis factor ligand superfamily member 10 isoform 1 | 4507593 | 33 | 1 | 4.30% | 1 | 1 | 4.30% | | 3 | | ni | ni | | ni |
| ubiquitin-conjugating enzyme E2 L3 isoform 3 | 373432682 | 14 | 1 | 18% | 2 | 1 | 12% | | 1 | | ni | ni | | ni |
| ATP-citrate synthase isoform X2 | 530412284 | 125 | 1 | 1.40% | 1 | ni | ni | | ni | | 3 | 3.20% | | 8 |
| myeloperoxidase precursor | 4557759 | 84 | 1 | 1.90% | 1 | ni | ni | | ni | | 2 | 3.20% | | 4 |
| ly6/PLAUR domain-containing protein 3 precursor | 93004088 | 36 | 1 | 5.80% | 1 | ni | ni | | ni | | 2 | 8.70% | | 5 |
| mannosyl-oligosaccharide glucosidase isoform 1 | 149999606 | 92 | 1 | 2.60% | 4 | ni | ni | | ni | | 2 | 7.60% | | 3 |
| alpha-1,3-mannosyl-glycoprotein 2-beta-N-acetylglucosaminyltransferase isoform X1 | 530381056 | 51 | 1 | 4.00% | 3 | ni | ni | | ni | | 1 | 4.00% | | 1 |
| stress-induced-phosphoprotein 1 isoform a | 544063423 | 68 | 1 | 3.90% | 1 | ni | ni | | ni | | 1 | 3.90% | | 2 |
| biglycan preproprotein | 4502403 | 42 | 1 | 3.00% | 2 | ni | ni | | ni | | 1 | 3.00% | | 3 |
| nicotinate-nucleotide pyrophosphorylase [carboxylating] isoform X1 | 530407851 | 31 | 1 | 13% | 1 | ni | ni | | ni | | 1 | 13% | | 2 |
| lipolysis-stimulated lipoprotein receptor isoform 4 | 386781455 | 69 | 1 | 2.20% | 3 | ni | ni | | ni | | ni | ni | | ni |
| ferritin heavy chain | 56682959 | 21 | 1 | 6.00% | 2 | ni | ni | | ni | | ni | ni | | ni |
| rab GDP dissociation inhibitor alpha | 4503971 | 51 | 1 | 13% | 2 | ni | ni | | ni | | ni | ni | | ni |
| vesicle-associated membrane protein 2 | 172072620 | 13 | 1 | 21% | 1 | ni | ni | | ni | | ni | ni | | ni |
| sperm acrosome-associated protein 5 precursor | 120952755 | 18 | ni | ni | ni | 4 | 31% | | 10 | | 2 | 13% | | 3 |
| tumor susceptibility gene 101 protein isoform X1 | 530395742 | 43 | ni | ni | ni | 3 | 10% | | 4 | | 2 | 5.20% | | 2 |
| probable serine carboxypeptidase CPVL isoform X1 | 530384848 | 54 | ni | ni | ni | 3 | 13% | | 6 | | 1 | 2.90% | | 3 |
| 14-3-3 protein sigma | 5454052 | 28 | ni | ni | ni | 3 | 17% | | 6 | | ni | ni | | ni |
| polypeptide N-acetylgalactosaminyltransferase 6 isoform X1 | 530399679 | 71 | ni | ni | ni | 2 | 7.20% | | 4 | | 4 | 14% | | 6 |
| trypsin-1 preproprotein | 4506145 | 27 | ni | ni | ni | 2 | 8.10% | | 6 | | 3 | 8.10% | | 9 |
| transforming growth factor beta-1 precursor | 63025222 | 44 | ni | ni | ni | 2 | 8.70% | | 2 | | 2 | 10.00% | | 2 |
| delta-aminolevulinic acid dehydratase | 189083849 | 36 | ni | ni | ni | 2 | 12% | | 2 | | 2 | 8.80% | | 2 |
| fructose-bisphosphate aldolase C isoform X1 | 530410965 | 48 | ni | ni | ni | 2 | 5.80% | | 8 | | 2 | 12% | | 7 |
| bifunctional ATP-dependent dihydroxyacetone kinase/FAD-AMP lyase (cyclizing) isoform X2 | 530396578 | 55 | ni | ni | ni | 2 | 4.70% | | 2 | | 2 | 7.90% | | 2 |
| glucosylceramidase isoform 1 precursor | 54607043 | 60 | ni | ni | ni | 2 | 7.80% | | 5 | | 2 | 9.70% | | 4 |
| tartrate-resistant acid phosphatase type 5 isoform X1 | 530414914 | 37 | ni | ni | ni | 2 | 9.50% | | 3 | | 1 | 5.80% | | 1 |
| epithelial discoidin domain-containing receptor 1 isoform 5 precursor | 321400061 | 85 | ni | ni | ni | 2 | 3.50% | | 4 | | 1 | 1.40% | | 1 |
| dyslexia-associated protein KIAA0319-like protein isoform X3 | 578799789 | 116 | ni | ni | ni | 2 | 2.40% | | 4 | | ni | ni | | ni |
| F-actin-capping protein subunit beta isoform 2 | 330864679 | 31 | ni | ni | ni | 2 | 8.70% | | 2 | | ni | ni | | ni |
| multifunctional protein ADE2 isoform X1 | 578808622 | 94 | ni | ni | ni | 2 | 4.10% | | 4 | | ni | ni | | ni |
| fructose-1,6-bisphosphatase isozyme 2 | 22907028 | 37 | ni | ni | ni | 2 | 8.60% | | 2 | | ni | ni | | ni |
| prostate and testis expressed protein 3 precursor | 222136622 | 12 | ni | ni | ni | 2 | 22% | | 4 | | ni | ni | | ni |
| desmocollin-2 isoform Dsc2b preproprotein | 13435366 | 94 | ni | ni | ni | 2 | 2.80% | | 2 | | ni | ni | | ni |
| palmitoyl-protein thioesterase 1 isoform 1 precursor | 4506031 | 34 | ni | ni | ni | 2 | 10% | | 4 | | ni | ni | | ni |
| integral membrane protein 2B | 11527402 | 30 | ni | ni | ni | 2 | 8.60% | | 5 | | ni | ni | | ni |
| reticulocalbin-1 precursor | 4506455 | 39 | ni | ni | ni | 2 | 6.60% | | 3 | | ni | ni | | ni |
| importin-5 isoform X2 | 530423350 | 126 | ni | ni | ni | 1 | 1.80% | | 3 | | 2 | 3.50% | | 4 |
| dolichyl-diphosphooligosaccharide--protein glycosyltransferase subunit 2 isoform 2 precursor | 209413738 | 68 | ni | ni | ni | 1 | 6.80% | | 1 | | 2 | 12% | | 5 |
| vitamin K-dependent protein S preproprotein | 192447438 | 75 | ni | ni | ni | 1 | 1.90% | | 2 | | 2 | 7.20% | | 4 |
| ribose-phosphate pyrophosphokinase 2 isoform 2 | 4506129 | 35 | ni | ni | ni | 1 | 5.30% | | 3 | | 2 | 10% | | 4 |
| LETM1 and EF-hand domain-containing protein 1, mitochondrial isoform X3 | 578808155 | 83 | ni | ni | ni | 1 | 3.50% | | 3 | | 2 | 3.90% | | 10 |
| mitochondrial-processing peptidase subunit beta isoform X1 | 530386674 | 57 | ni | ni | ni | 1 | 4.10% | | 3 | | 2 | 7.90% | | 4 |
| actin-like protein 7A | 5729720 | 49 | ni | ni | ni | 1 | 5.50% | | 2 | | 2 | 12% | | 2 |
| UDP-glucose:glycoprotein glucosyltransferase 1 isoform X1 | 578804601 | 177 | ni | ni | ni | 1 | 1.70% | | 1 | | 1 | 1.70% | | 1 |
| alpha-2-HS-glycoprotein preproprotein | 156523970 | 39 | ni | ni | ni | 1 | 8.70% | | 1 | | 1 | 1.90% | | 1 |
| transcobalamin-2 isoform 1 precursor | 21071010 | 48 | ni | ni | ni | 1 | 6.60% | | 2 | | 1 | 6.60% | | 2 |
| plasminogen isoform 1 precursor | 4505881 | 91 | ni | ni | ni | 1 | 1.20% | | 1 | | ni | ni | | ni |
| fibulin-2 isoform a precursor | 51873053 | 132 | ni | ni | ni | 1 | 0.81% | | 1 | | ni | ni | | ni |
| GDNF family receptor alpha-2 isoform X1 | 530387711 | 53 | ni | ni | ni | 1 | 1.90% | | 1 | | ni | ni | | ni |
| delta and Notch-like epidermal growth factor-related receptor precursor | 116235485 | 78 | ni | ni | ni | 1 | 2.00% | | 1 | | ni | ni | | ni |
| reticulon-4 receptor-like 1 precursor | 30425553 | 49 | ni | ni | ni | 1 | 3.20% | | 2 | | ni | ni | | ni |
| sortilin isoform 1 preproprotein | 17149834 | 92 | ni | ni | ni | 1 | 1.80% | | 2 | | ni | ni | | ni |
| acyl-protein thioesterase 1 isoform 6 | 525342616 | 18 | ni | ni | ni | 1 | 7.80% | | 1 | | ni | ni | | ni |
| beta-1,4-galactosyltransferase 4 | 47078258 | 40 | ni | ni | ni | ni | ni | | ni | | 2 | 9.00% | | 3 |
| basal cell adhesion molecule isoform 1 precursor | 31543106 | 67 | ni | ni | ni | ni | ni | | ni | | 2 | 4.30% | | 4 |
| receptor-type tyrosine-protein phosphatase eta isoform 1 precursor | 148728162 | 146 | ni | ni | ni | ni | ni | | ni | | 2 | 3.00% | | 2 |
| phospholipase B-like 1 precursor | 110227598 | 63 | ni | ni | ni | ni | ni | | ni | | 1 | 2.20% | | 1 |
| interleukin-1 receptor-like 1 isoform X1 | 578805137 | 63 | ni | ni | ni | ni | ni | | ni | | 1 | 1.80% | | 1 |
| fibrinogen gamma chain isoform gamma-A precursor | 70906437 | 49 | ni | ni | ni | ni | ni | | ni | | 1 | 4.60% | | 1 |
|  |  |  |  |  |  |  |  | |  | |  |  | |  |
